# Supplementary material for: Industrialization as a source of heavy metals and antibiotics which can enhance the antibiotic resistance in wastewater, sewage sludge and river water
Source: PLoS One. 2021 Jun 4;16(6):e0252691. doi: 10.1371/journal.pone.0252691 (PMC8177550; doi:10.1371/journal.pone.0252691)
Supplement: S1 File — (DOCX) [file pone.0252691.s001.docx]

Title:

Industrialization as a source of heavy metals and antibiotics which can enhance the antibiotic resistance in wastewater, sewage sludge and river water

Authors:

Jakub Hubeny ^[1]^, Monika Harnisz ^[1]*^, Ewa Korzeniewska ^[1]^, Martyna Buta ^[1]^, Wiktor Zieliński ^[1]^, Damian Rolbiecki ^[1]^, Joanna Giebułtowicz ^[2]^, Grzegorz Nałęcz-Jawecki ^[3]^, Grażyna Płaza ^[4]^

Author affiliations:

^[1]^ Department of Water Protection Engineering and Environmental Microbiology, Faculty of Geoengineering, University of Warmia and Mazury in Olsztyn, Prawocheńskiego 1, 10-720 Olsztyn, Poland; monikah@uwm.edu.pl (M.H.)

^[2]^ Department of Bioanalysis and Drug Analysis, Faculty of Pharmacy, Medical University of Warsaw, Banacha 1, 02-097 Warszawa, Poland

^[3]^ Department of Environmental Health Sciences, Faculty of Pharmacy, Medical University of Warsaw, Banacha 1, 02-097 Warszawa, Poland

^[4]^ Faculty of Organization and Management, Silesian University of Technology, Roosevelt 1, 41-800 Zabrze, Poland


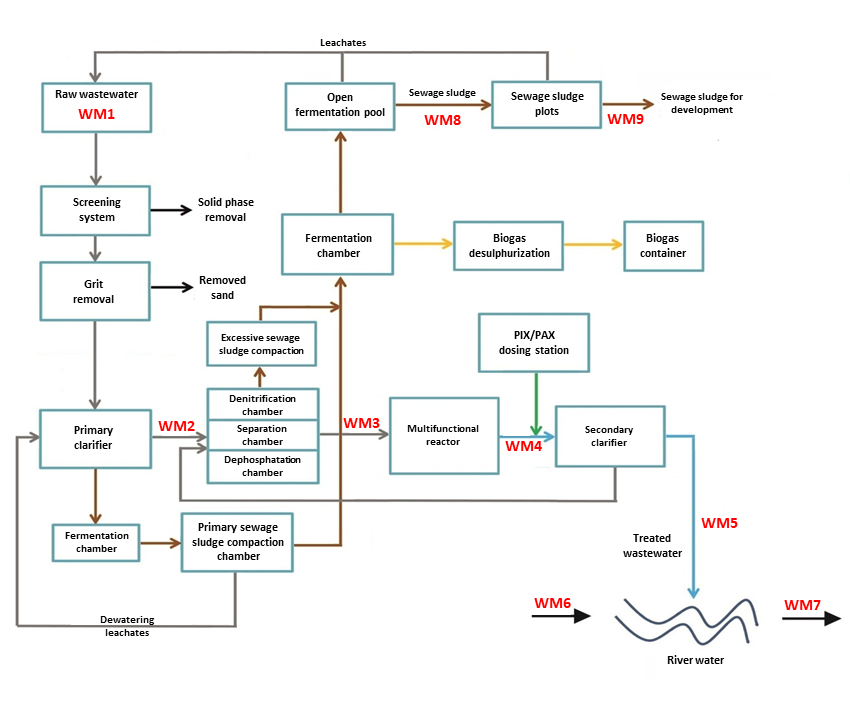


S1 Fig. Diagram of WM-WWTP with marked collection samples sites (red).


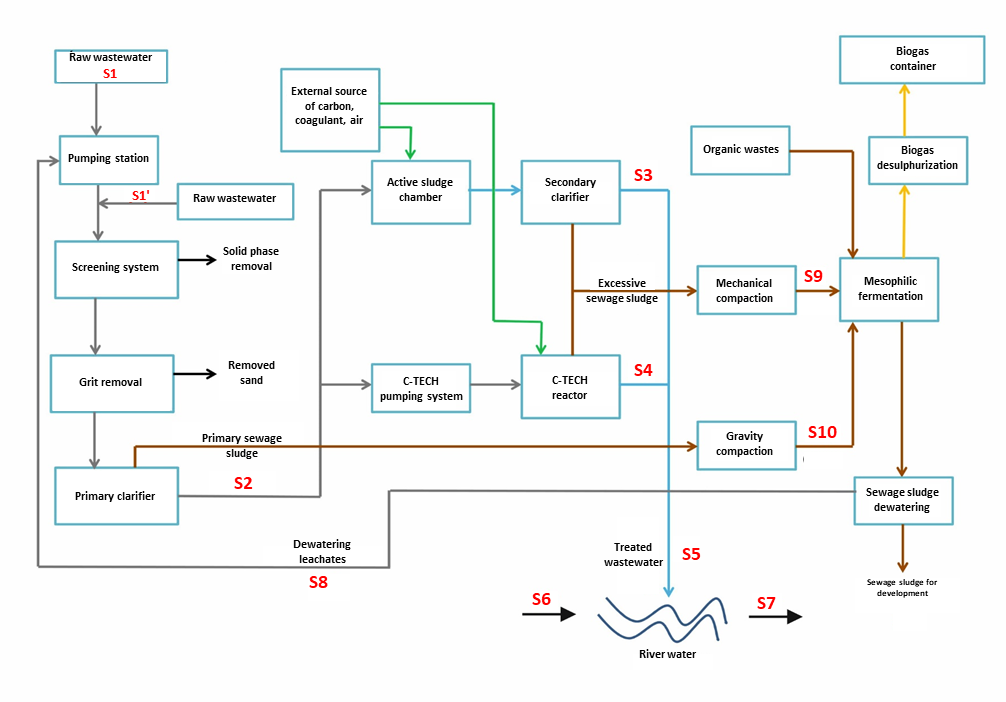


S2 Fig. Diagram of S-WWTP with marked collection samples sites (red)

S1 Table Primers used in qPCR reaction.

| Primer | | 3’🡪 5’primer sequence | Amplification product size (bp) | References | Annealing temperature (℃) | PCR Amplification Efficiency (%) |
| --- | --- | --- | --- | --- | --- | --- |
| *bla*_TEM_ | F | AGTGCTGCCATAACCATGAGTG | 431 | [1] | 61 | 84 |
|  | R | CTGACTCCCCGTCGTGTAGATA |  |  |  |  |
| *bla*_OXA_ | F | ATTATCTACAGCAGCGCCAGTG | 296 | [1] | 61 | 88 |
|  | R | TGCATCCACGTCTTTGGTG |  |  |  |  |
| *bla*_SHV_ | F | GATGAACGCTTTCCCATGATG | 214 | [1] | 61 | 68 |
|  | R | CGCTGTTATCGCTCATGGTAA |  |  |  |  |
| *tet*(A) | F | GCTACATCCTGCTTGCCTTC | 211 | [2] | 53 | 72 |
|  | R | GCATAGATCGCCGTGAAGAG |  |  |  |  |
| *tet*(M) | F | GTGGACAAAGGTACAACGAG | 406 | [3] | 55 | 82 |
|  | R | CGGTAAAGTTCGTCACACAC |  |  |  |  |
| *int*I1 | F | CCTCCCGCACGATGATC | 280 | [4] | 55 | 90 |
|  | R | TCCACGCATCGTCAGGC |  |  |  |  |
| *int*I2 | F | TTATTGCTGGGATTAGGC | 233 | [4] | 50 | 78 |
|  | R | ACGGCTACCCTCTGTTATC |  |  |  |  |
| *qep*A | F | CCAGCTCGGCAACTTGATAC | 570 | [5] | 60 | 62 |
|  | R | ATGCTCGCCTTCCAGAAAA |  |  |  |  |
| *aac*(6`)-*Ib*-*cr* | F | TTGCGATGCTCTATGAGTGGCTA | 482 | [6] | 55 | 78 |
|  | R | CTCGAATGCCTGGCGTGTTT |  |  |  |  |
| *sul*1 | F | CGCACCGGAAACATCGCTGCAC | 163 | [7] | 65 | 95 |
|  | R | TGAAGTTCCGCCGCAAGGCTCG |  |  |  |  |
| *sul*2 | F | TCCGGTGGAGGCCGGTATCTGG | 191 | [7] | 57.5 | 91 |
|  | R | CGGGAATGCCATCTGCCTTGAG |  |  |  |  |
| 16S rRNA | F | TCCTACGGGAGGCAGCAGT | 466 | [8] | 60 | 94 |
|  | R | GGACTACCAGGGTATCTAATCCTGTT |  |  |  |  |

**Instrumental analysis of antibiotic determination by HPLC-MS/MS**

Instrumental analyses were performed by HPLC–MS/MS according to a previously described procedure [9]. The analyses were conducted with the use of the Agilent 1260 Infinity (Agilent Technologies, Santa Clara, CA, USA) liquid chromatograph coupled to the Hybrid Triple Quadrupole/Linear Ion trap mass spectrometer (QTRAP®4000, AB SCIEX, Framingham, MA, USA). Chromatographic separation was achieved with a Kinetex RP-18 column (100 mm × 4.6 mm, 2.6 µm) supplied by Phenomenex (Torrance, CA, USA). The mobile phase was 0.2% formic acid (eluent A) and acetonitrile with 0.2% formic acid (eluent B). The flow rate was 0.5 mL min^-1^, and the gradient (%B) was: 0 min. 10%; 1 min. 10%; 25 min. 90%, 35 min. 90%. The target compounds were analyzed in positive ionization mode (ESI +), and two transitions between the precursor ion and selected fragment ions were monitored for each compound. The monitored transition values for the analyzed antibiotics are presented in Table A.4.

References:

[1] J. Kim, S. Jeon, H. Rhie, B. Lee, M. Park, H. Lee, J. Lee, S. Kim, Rapid Detection of Extended Spectrum β-Lactamase (ESBL) for Enterobacteriaceae by use of a Multiplex PCR-based Method, Infect. Chemother. 41 (2009) 181. https://doi.org/10.3947/ic.2009.41.3.181.

[2] M. Nawaz, K. Sung, S.A. Khan, A.A. Khan, R. Steele, Biochemical and molecular characterization of tetracycline-resistant Aeromonas veronii isolates from catfish, Appl. Environ. Microbiol. 72 (2006) 6461–6466. https://doi.org/10.1128/AEM.00271-06.

[3] L.K. Ng, I. Martin, M. Alfa, M. Mulvey, Multiplex PCR for the detection of tetracycline resistant genes, Mol. Cell. Probes. 15 (2001) 209–215. https://doi.org/10.1006/mcpr.2001.0363.

[4] C. Goldstein, M.D. Lee, S. Sanchez, C. Hudson, B. Phillips, B. Register, M. Grady, C. Liebert, A.O. Summers, D.G. White, J.J. Maurer, Incidence of class 1 and 2 integrases in clinical and commensal bacteria from livestock, companion animals, and exotics, Antimicrob. Agents Chemother. 45 (2001) 723–726. https://doi.org/10.1128/AAC.45.3.723-726.2001.

[5] J. Li, T. Wang, B. Shao, J. Shen, S. Wang, Y. Wu, Plasmid-mediated quinolone resistance genes and antibiotic residues in wastewater and soil adjacent to swine feedlots: Potential transfer to agricultural lands, Environ. Health Perspect. 120 (2012) 1144–1149. https://doi.org/10.1289/ehp.1104776.

[6] C.H. Park, A. Robicsek, G.A. Jacoby, D. Sahm, D.C. Hooper, Prevalence in the United States of aac(6′)-Ib-cr encoding a ciprofloxacin-modifying enzyme, Antimicrob. Agents Chemother. 50 (2006) 3953–3955. https://doi.org/10.1128/AAC.00915-06.

[7] R. Pei, S.C. Kim, K.H. Carlson, A. Pruden, Effect of River Landscape on the sediment concentrations of antibiotics and corresponding antibiotic resistance genes (ARG), Water Res. 40 (2006) 2427–2435. https://doi.org/10.1016/j.watres.2006.04.017.

[8] M.A. Nadkarni, F.E. Martin, N.A. Jacques, N. Hunter, Determination of bacterial load by real-time PCR using a broad-range (universal) probe and primers set, Microbiology. 148 (2002) 257–266. https://doi.org/10.1099/00221287-148-1-257.

[9] J. Giebułtowicz, S. Tyski, R. Wolinowska, W. Grzybowska, T. Zaręba, A. Drobniewska, P. Wroczyński, G. Nałęcz-Jawecki, Occurrence of antimicrobial agents, drug-resistant bacteria, and genes in the sewage-impacted Vistula River (Poland), Environ. Sci. Pollut. Res. 25 (2018) 5788–5807. https://doi.org/10.1007/s11356-017-0861-x.

S2 Table Average concentration of antibiotics (ng/g) in samples from both WWTPs.

| Sample sites | | CIP | NOR | OFX | PEF | SDM | SXT | ST | TET | OTC |
| --- | --- | --- | --- | --- | --- | --- | --- | --- | --- | --- |
| WM-WWTP | WM1 | 5.8494 | <0.0063 | 0.2089 | <0.012 | 0.0124 | 2.9861 | 0.2335 | 0.1221 | <0.0001 |
|  | WM2 | 4.7842 | <0.0063 | 0.1488 | <0.012 | 0.0135 | 0.8803 | 0.1143 | 0.0642 | <0.0001 |
|  | WM3 | 0.8216 | <0.0063 | 0.0629 | <0.012 | 0.0093 | 0.7706 | 0.0915 | 0.0986 | <0.0001 |
|  | WM4 | 0.5571 | <0.0063 | 0.0664 | <0.012 | 0.0056 | 1.2207 | 0.0350 | 0.1306 | <0.0001 |
|  | WM5 | 0.3279 | <0.0063 | 0.0333 | <0.012 | 0.0068 | 0.9286 | 0.0465 | 0.0619 | <0.0001 |
|  | WM6 | 0.0128 | <0.0063 | 0.0084 | <0.012 | <0.0018 | <0.0059 | <0.0024 | <0.0002 | <0.0001 |
|  | WM7 | 0.1827 | <0.0063 | 0.0313 | <0.012 | <0.0018 | 0.0761 | <0.0024 | 0.0072 | <0.0001 |
|  | WM8 | 343.3900 | 53.4925 | 9.7150 | 4.7350 | <0.0600 | <2.28 | <3.34 | 3.2125 | 1.9225 |
|  | WM9 | 1179.9200 | 319.7420 | 66.1140 | 29.0140 | 1.5920 | <2.28 | <3.34 | 11.0100 | 7.2120 |
| S-WWTP | S1 | 1.3833 | 0.3030 | 0.1384 | <0.012 | <0.0018 | 1.1803 | 0.0633 | 0.0619 | 0.0010 |
|  | S2 | 1.1935 | 0.2242 | 0.0934 | <0.012 | 0.0083 | 0.8838 | 0.0498 | 0.0478 | 0.0009 |
|  | S3 | 0.1348 | 0.0032 | 0.1179 | <0.012 | <0.0018 | 0.5793 | 0.0155 | 0.0006 | <0.0001 |
|  | S4 | 0.0885 | 0.1702 | 0.0129 | <0.012 | <0.0018 | 0.4872 | 0.0105 | 0.0003 | 0.0004 |
|  | S5 | 0.1352 | 0.0356 | 0.0171 | <0.012 | <0.0018 | 0.4869 | 0.0121 | 0.0003 | <0.0001 |
|  | S6 | 0.1078 | 0.0954 | 0.0316 | <0.012 | <0.0018 | 0.6444 | 0.0073 | <0.0002 | <0.0001 |
|  | S7 | 0.0951 | <0.0063 | 0.0047 | <0.012 | <0.0018 | 0.4514 | 0.0291 | 0.0004 | 0.0004 |
|  | S8 | 1.0435 | 0.1650 | 0.1377 | <0.012 | <0.0018 | 0.6916 | 0.0987 | 0.0654 | 0.0016 |
|  | S9 | 252.7083 | 137.8067 | 26.7433 | 7.2000 | 0.3450 | 2.6750 | <3.34 | 1.7367 | 10.2417 |
|  | S10 | 412.2600 | 182.8067 | 23.0783 | 8.0567 | 0.3483 | <2.28 | <3.34 | 2.6800 | 17.9233 |
| Mean | | 116.0524 | 36.5721 | 6.6718 | 2.5871 | 0.1252 | 0.9604 | 0.3942 | 1.0158 | 1.9634 |


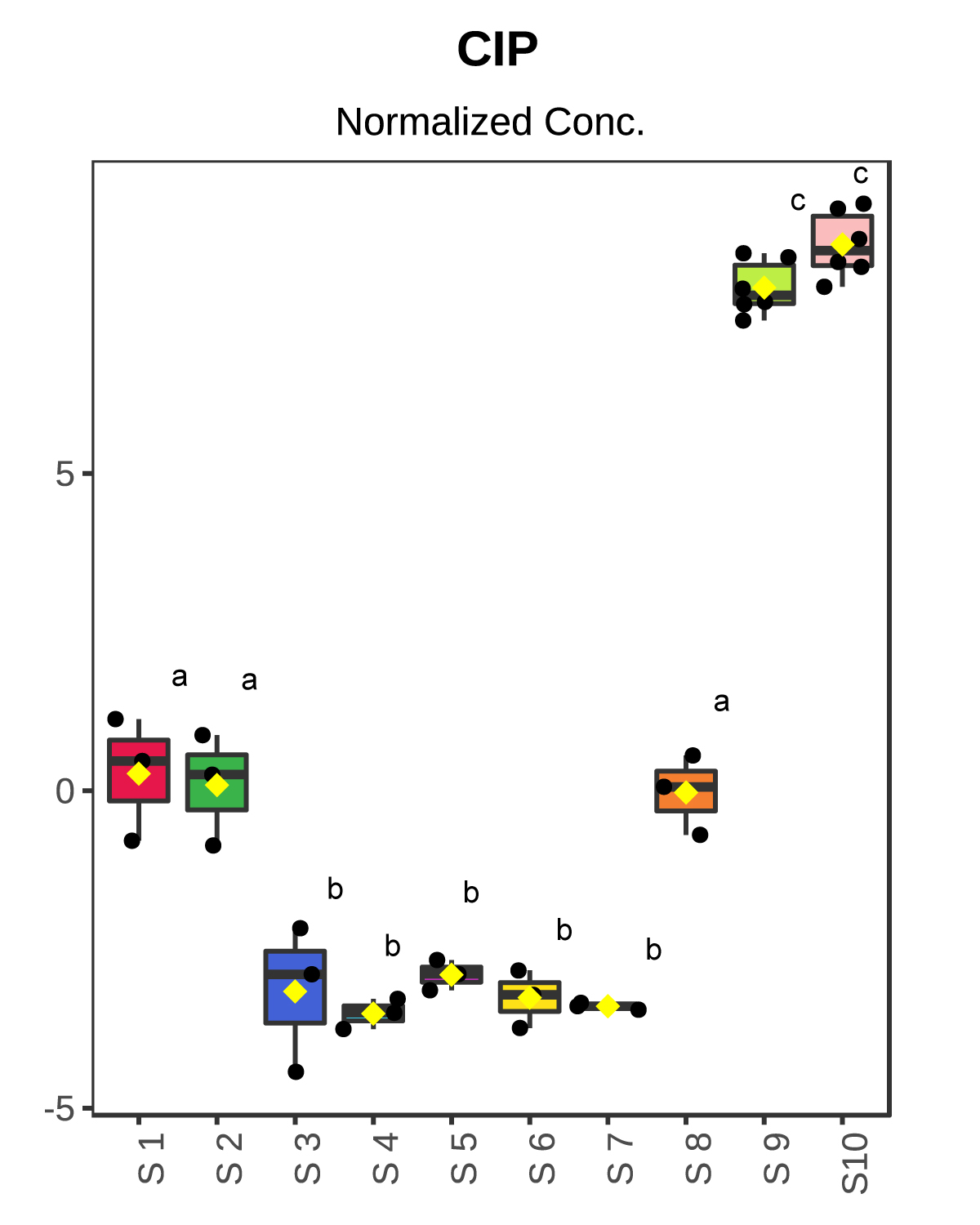


**B**


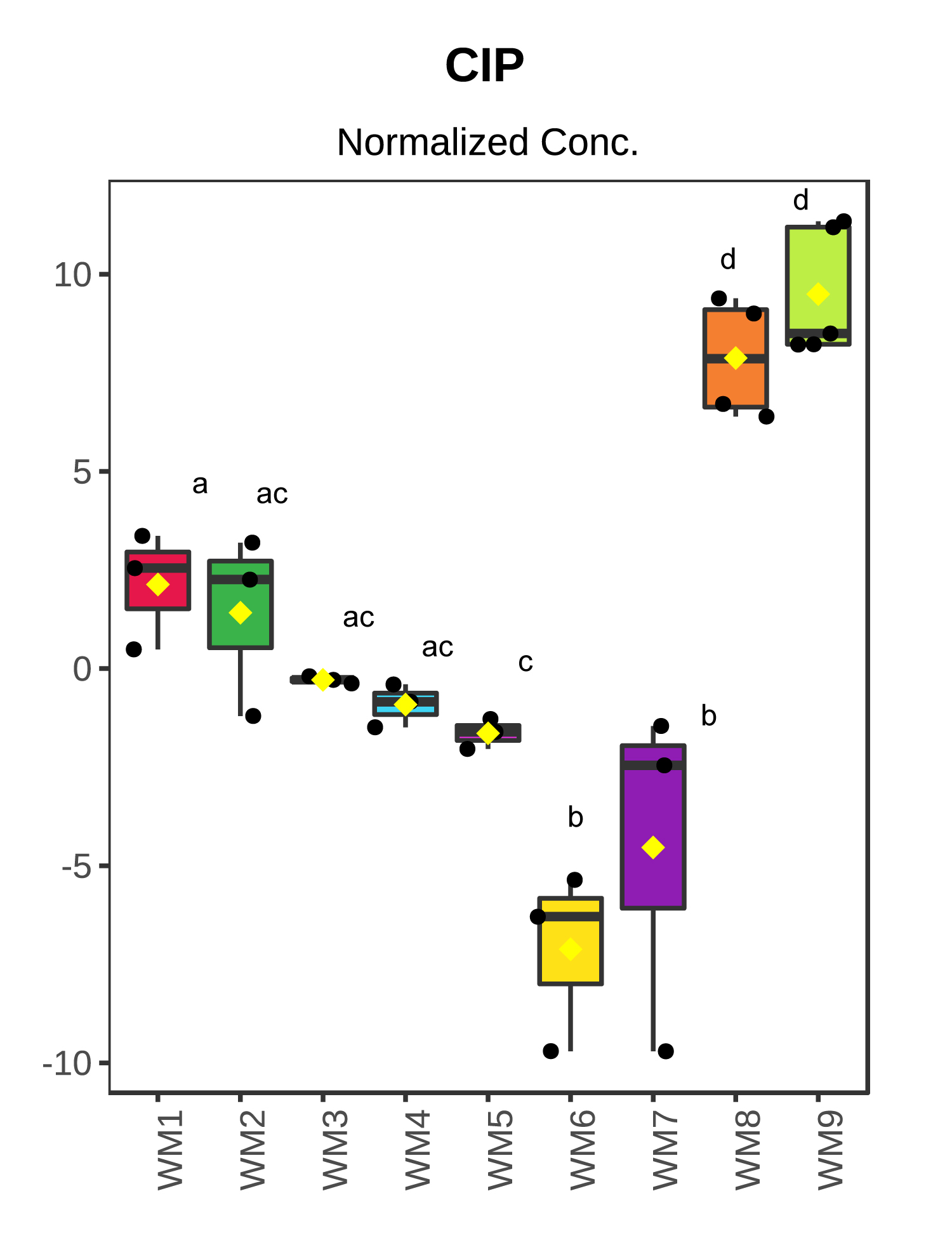


**A**

S3 Fig. Normalized concentration of ciprofloxacin in samples from WM-WWTP (A) and S-WWTP (B). The graph shows the deviation from the average concentration of CIP. The data were normalized by dividing them by the mean concentration of all variables and cases (separately for S-WWTP and WM-WWTP). The letters stands for homogeneous groups, sites with same symbols indicate no statistical differences.


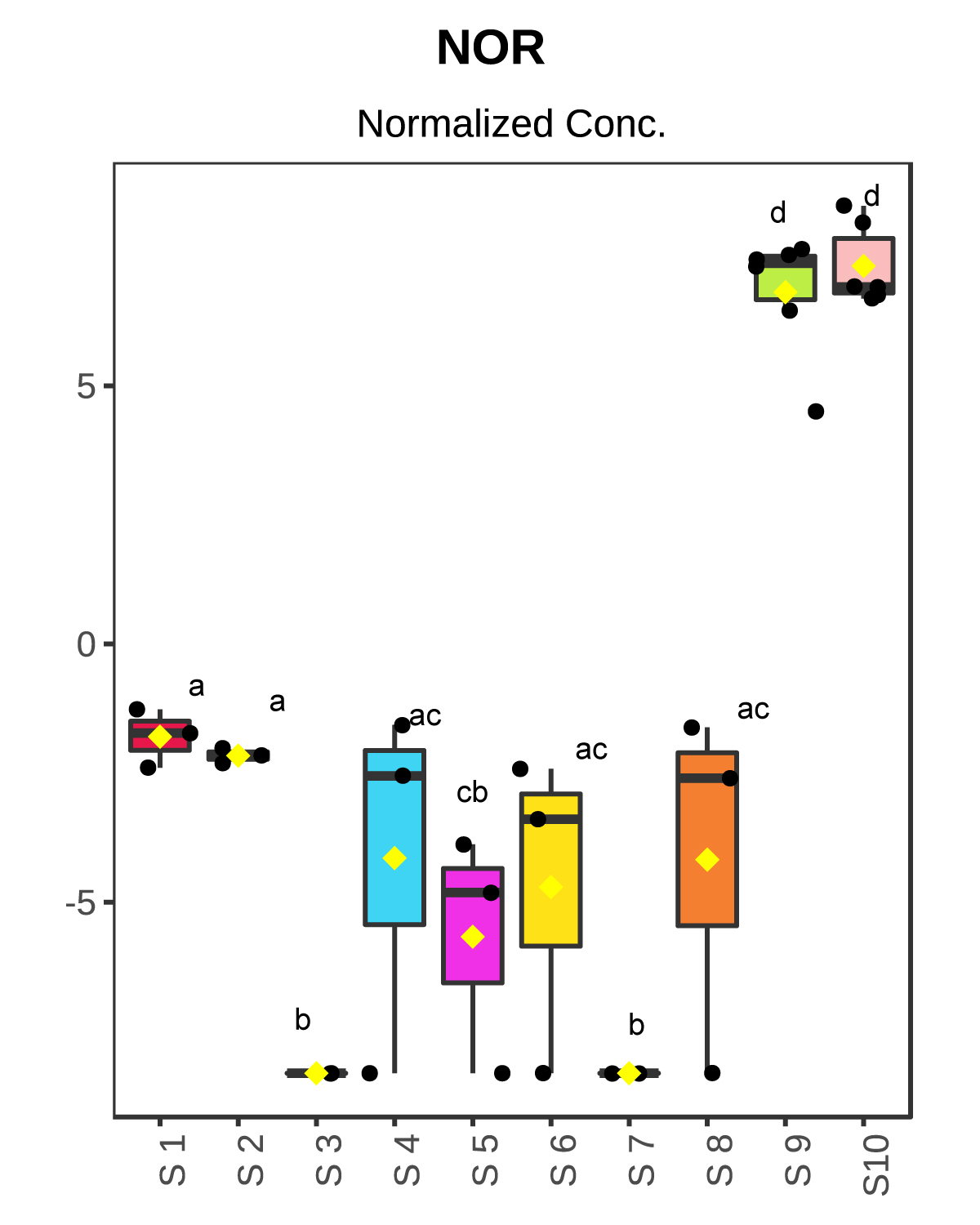


**B**


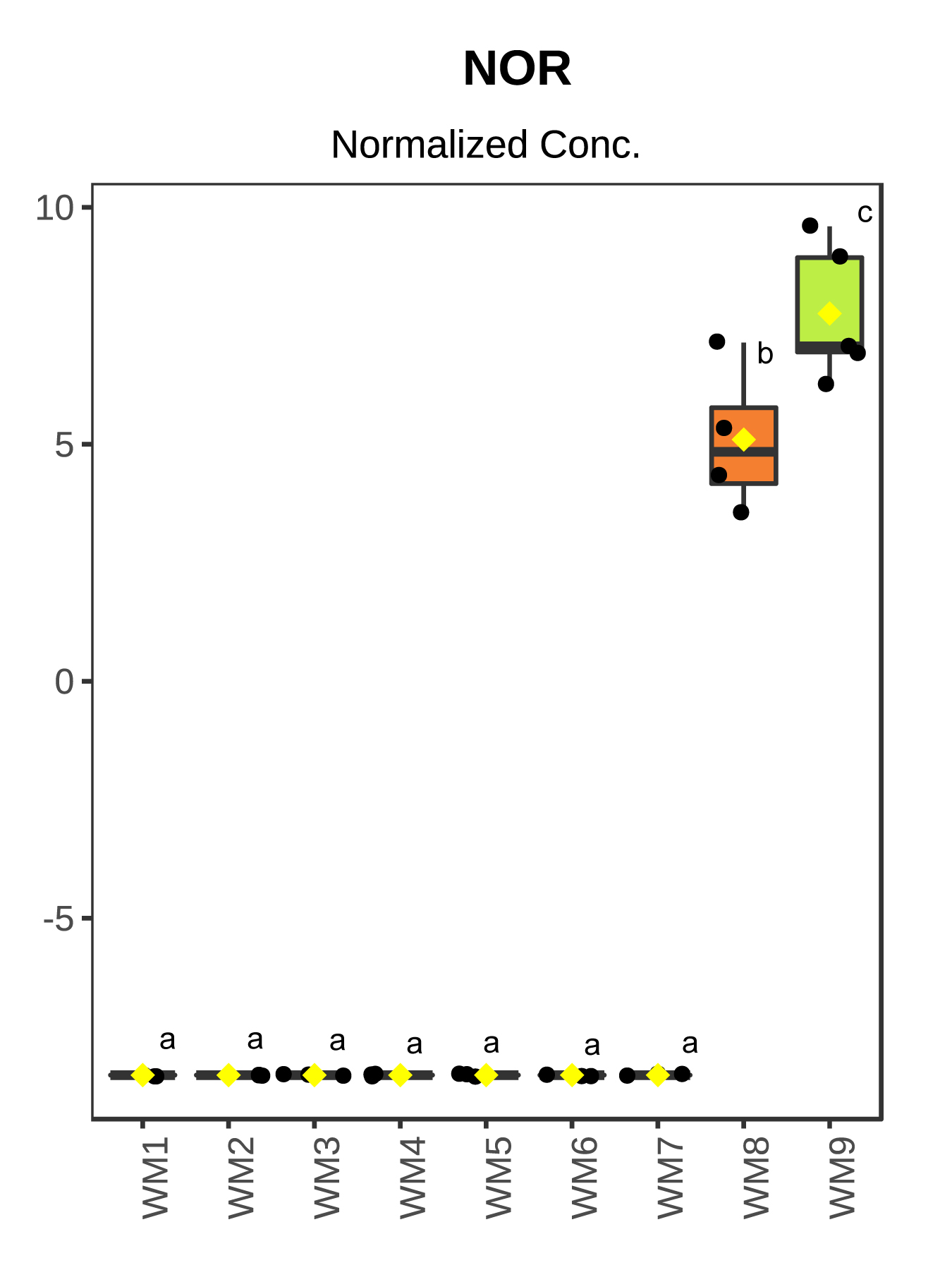


**A**

**S4 Fig. Normalized concentration of norfloxacin in samples from WM-WWTP (A) and S-WWTP (B).** The graph shows the deviation from the average concentration of NOR. The data were normalized by dividing them by the mean concentration of all variables and cases (separately for S-WWTP and WM-WWTP). The letters stands for homogeneous groups, sites with same symbols indicate no statistical differences.


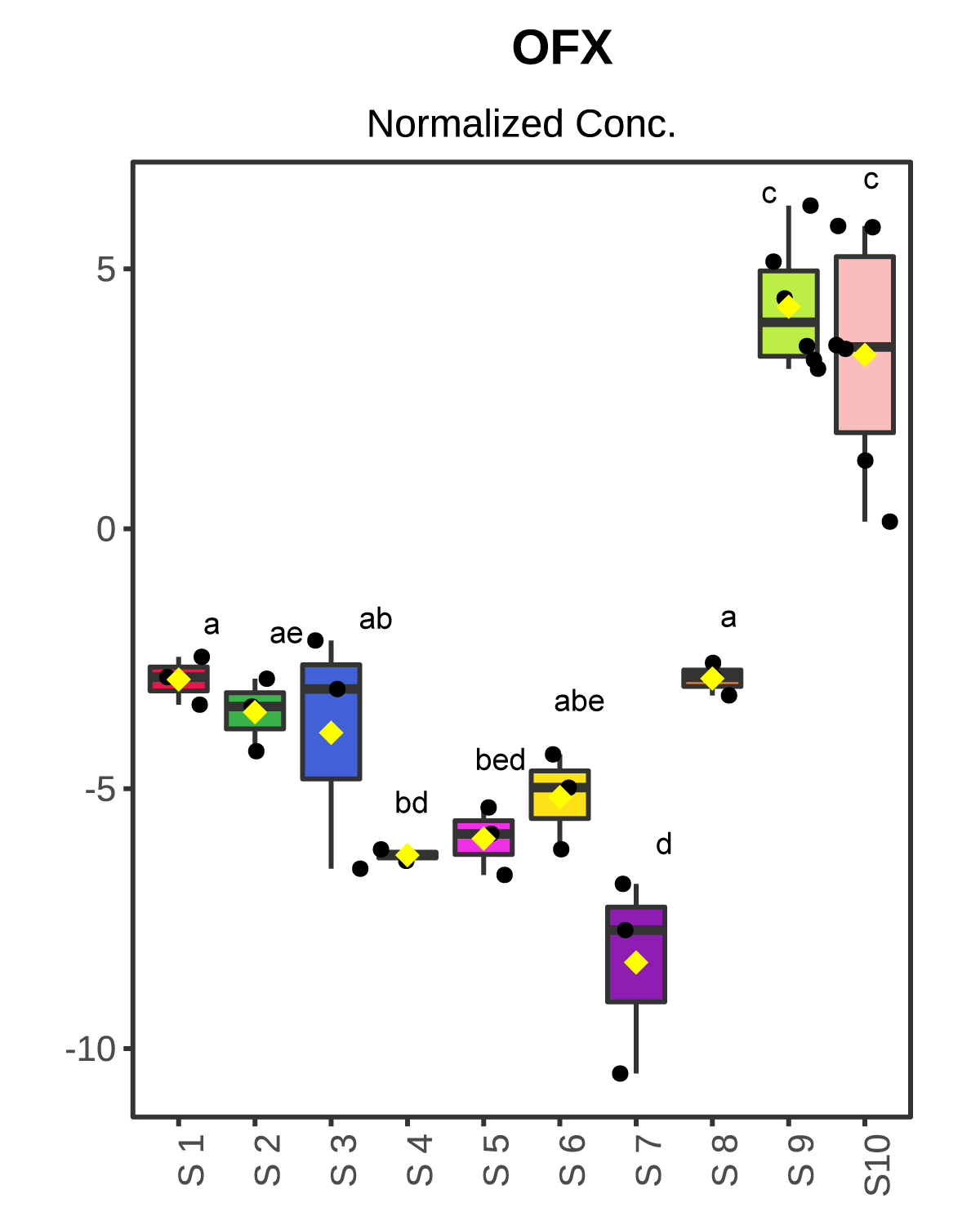


**B**


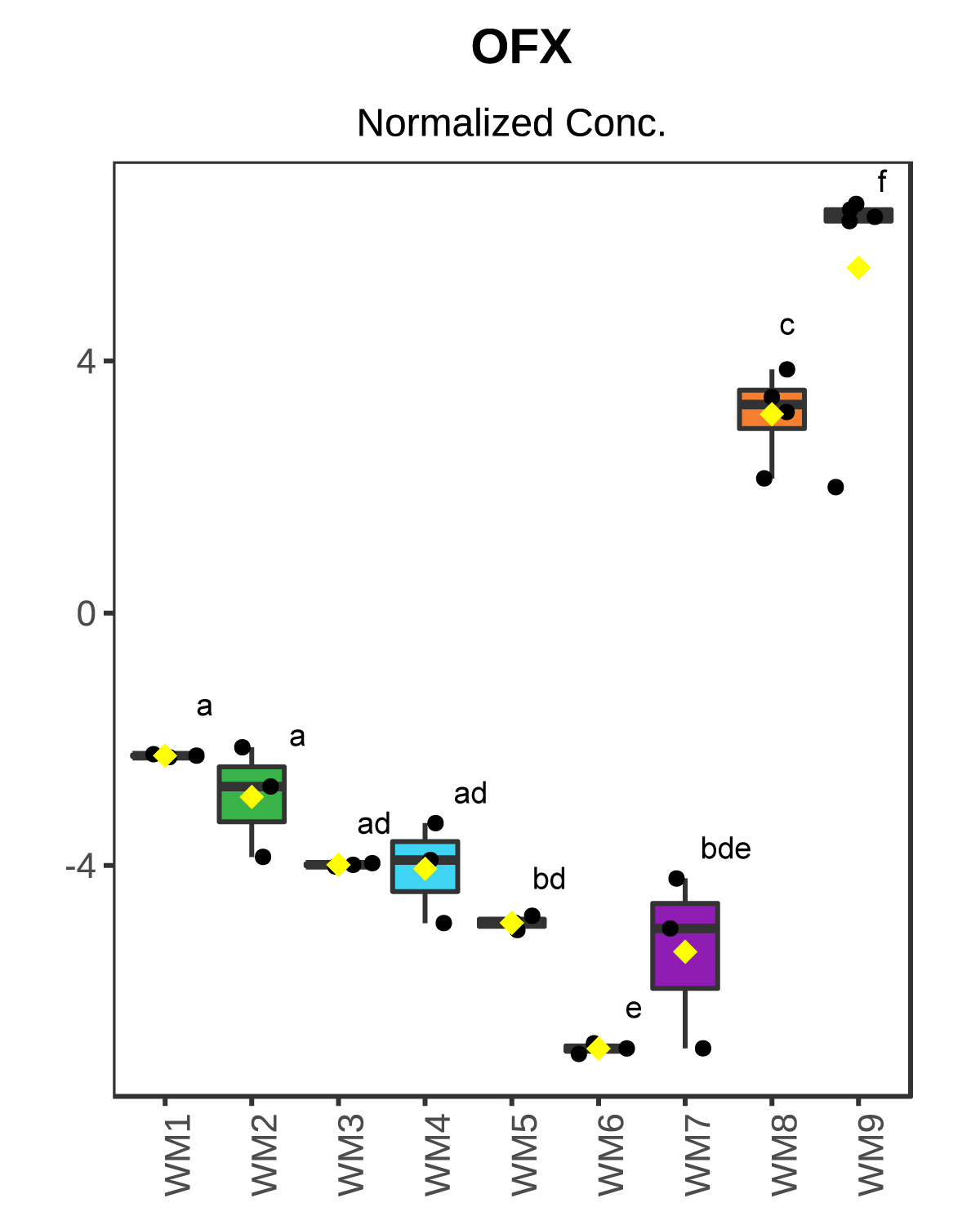


**A**

S5 Fig. Normalized concentration of ofloxacin in samples from WM-WWTP (A) and S-WWTP (B). The graph shows the deviation from the average concentration of OFX. The data were normalized by dividing them by the mean concentration of all variables and cases (separately for S-WWTP and WM-WWTP). The letters stands for homogeneous groups, sites with same symbols indicate no statistical differences.


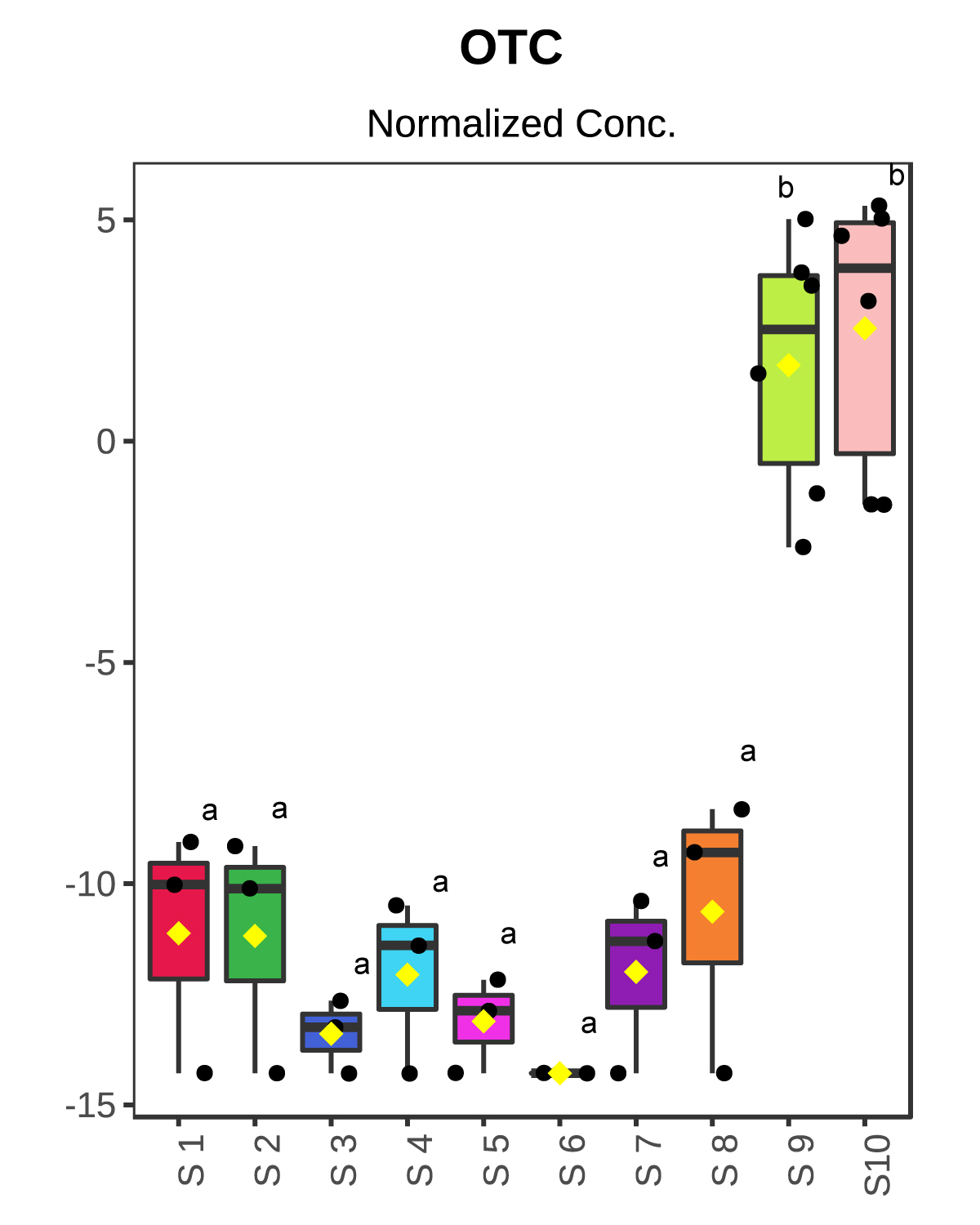


**B**


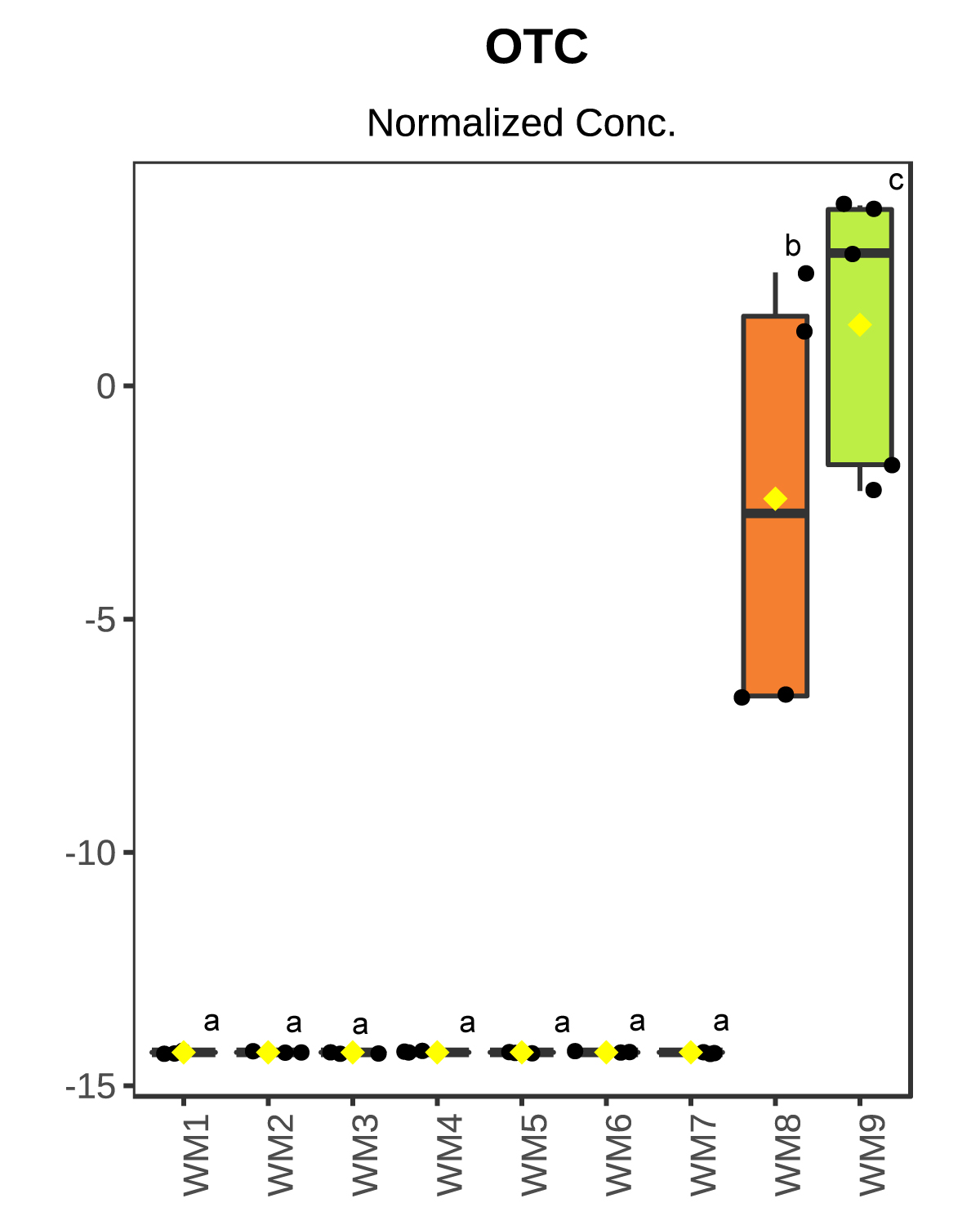


**A**

S6 Fig. Normalized concentration of oxytetracycline in samples from WM-WWTP (A) and S-WWTP (B). The graph shows the deviation from the average concentration of OTC. The data were normalized by dividing them by the mean concentration of all variables and cases (separately for S-WWTP and WM-WWTP). The letters stands for homogeneous groups, sites with same symbols indicate no statistical differences.


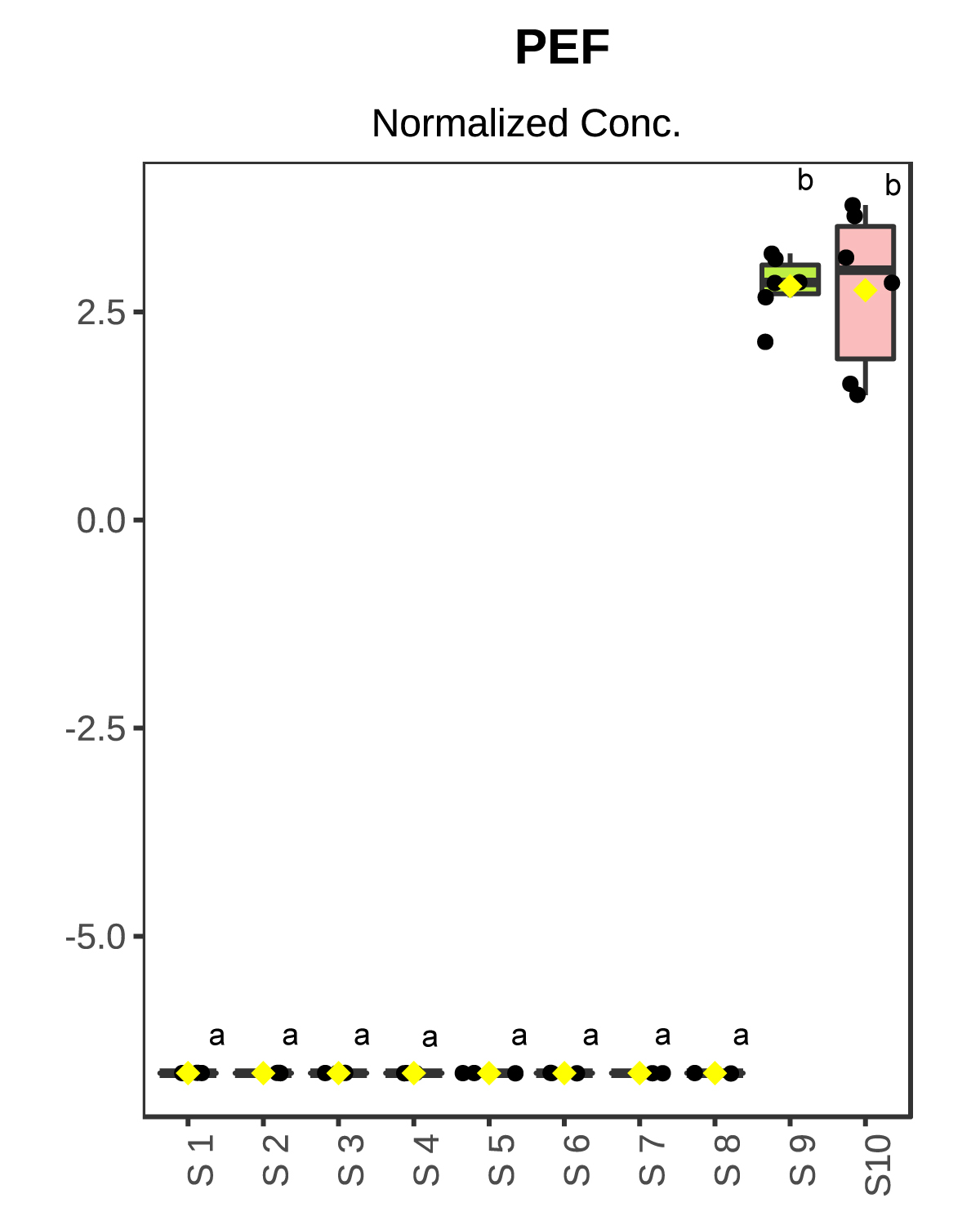


**B**


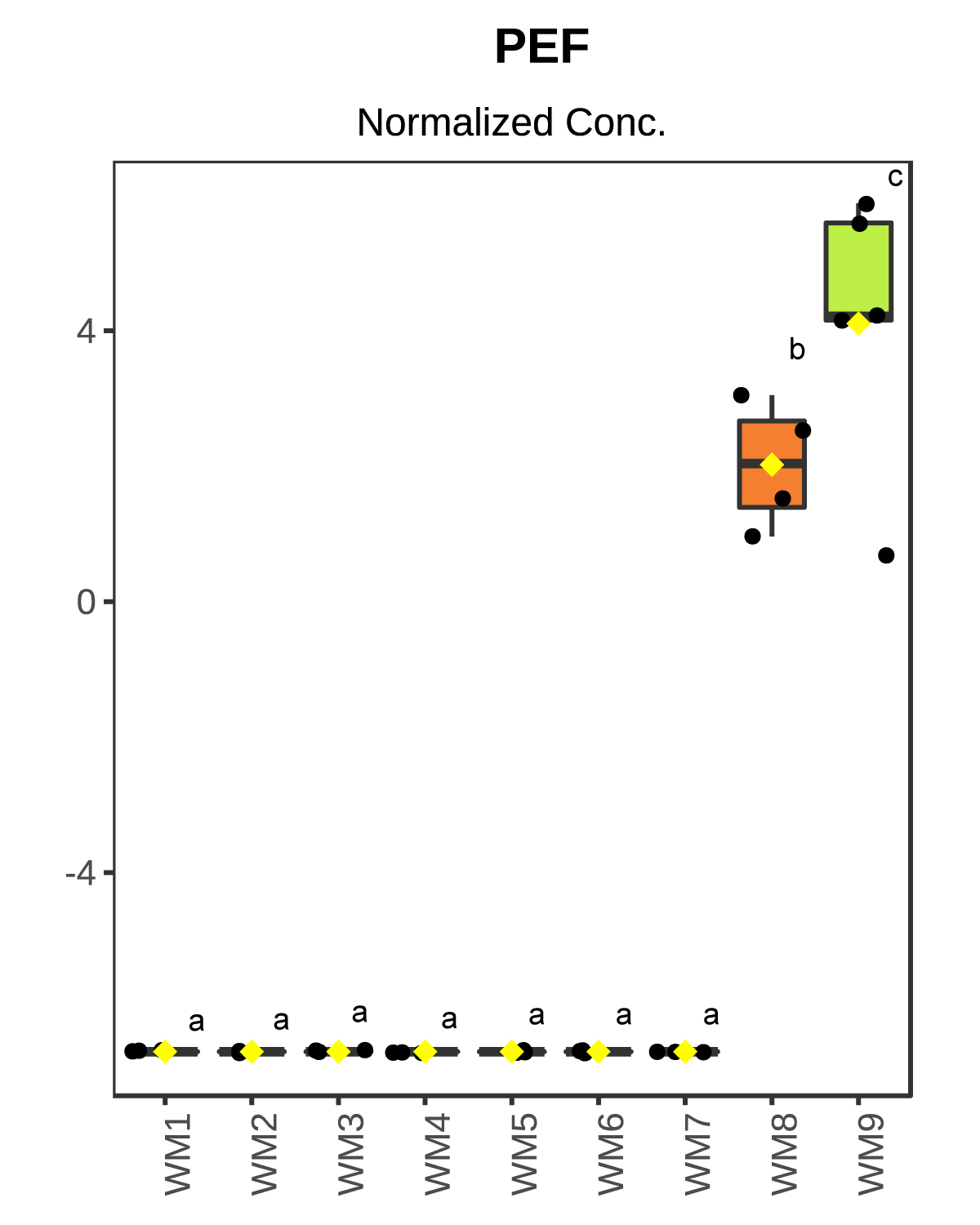


**A**

S7 Fig. Normalized concentration of perfloxacin in samples from WM-WWTP (A) and S-WWTP (B). The graph shows the deviation from the average concentration of PEF. The data were normalized by dividing them by the mean concentration of all variables and cases (separately for S-WWTP and WM-WWTP). The letters stands for homogeneous groups, sites with same symbols indicate no statistical differences.


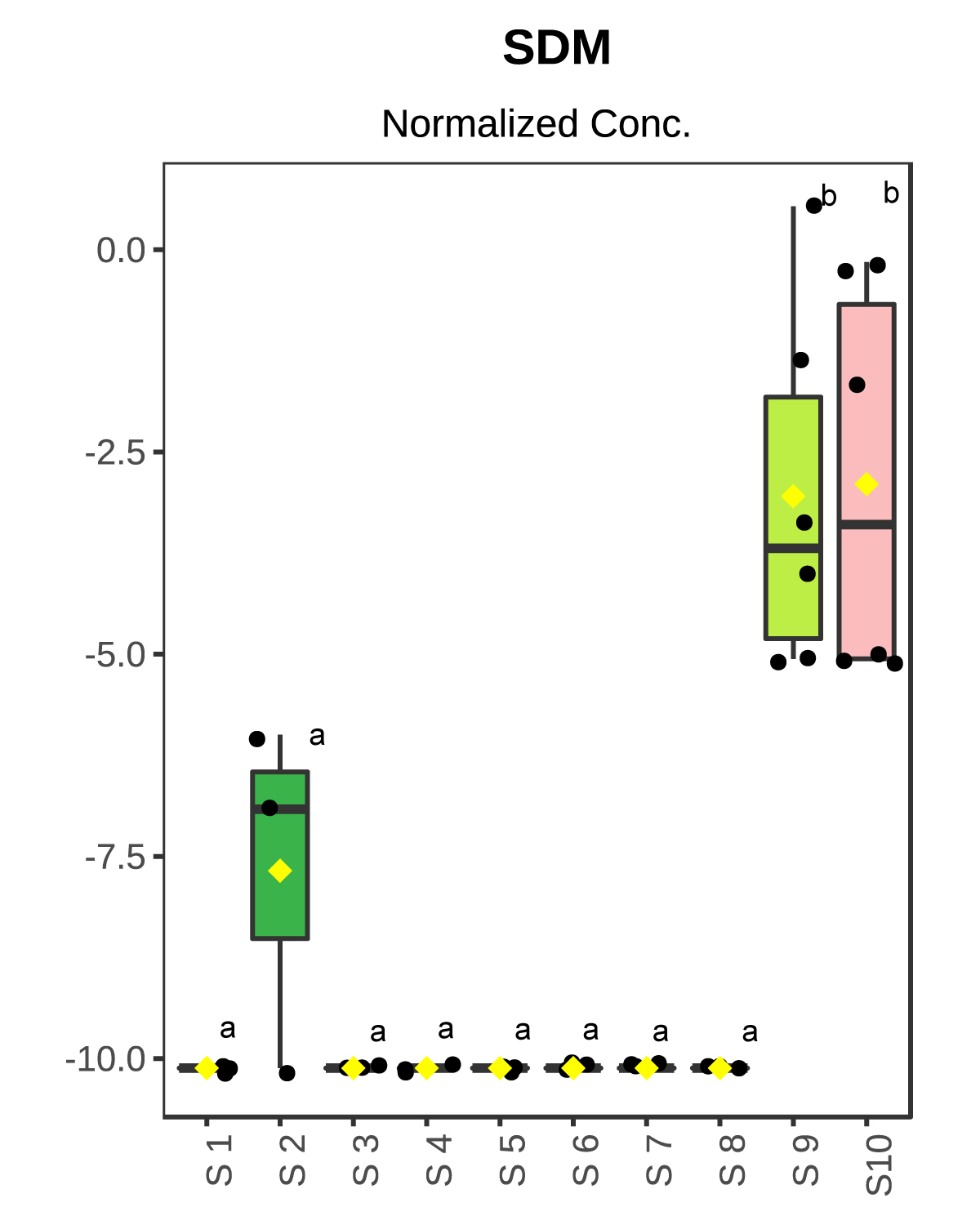


**B**


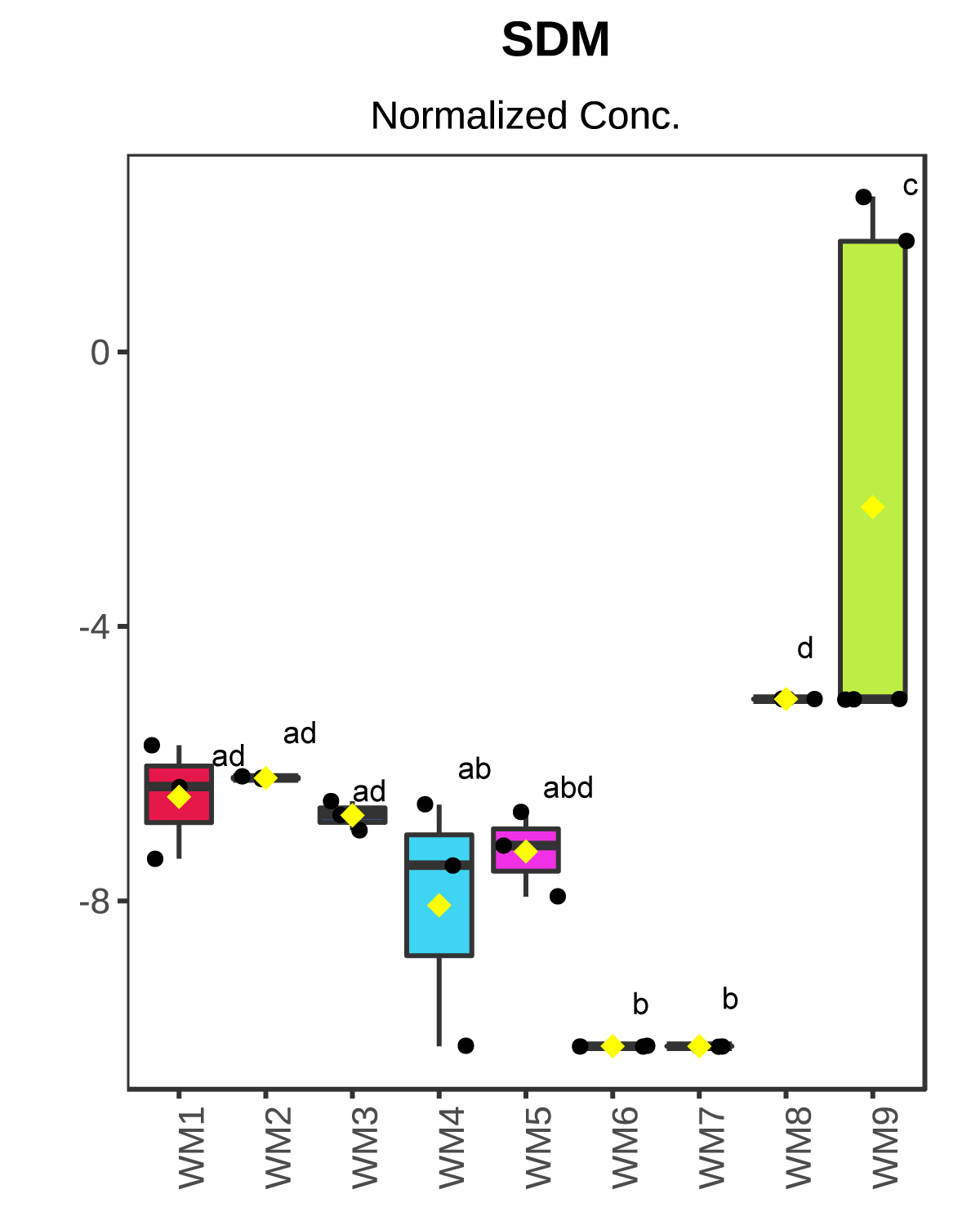


**A**

S8 Fig. Normalized concentration of sulfadimethoxine in samples from WM-WWTP (A) and S-WWTP (B). The graph shows the deviation from the average concentration of SDM. The data were normalized by dividing them by the mean concentration of all variables and cases (separately for S-WWTP and WM-WWTP). The letters stands for homogeneous groups, sites with same symbols indicate no statistical differences.


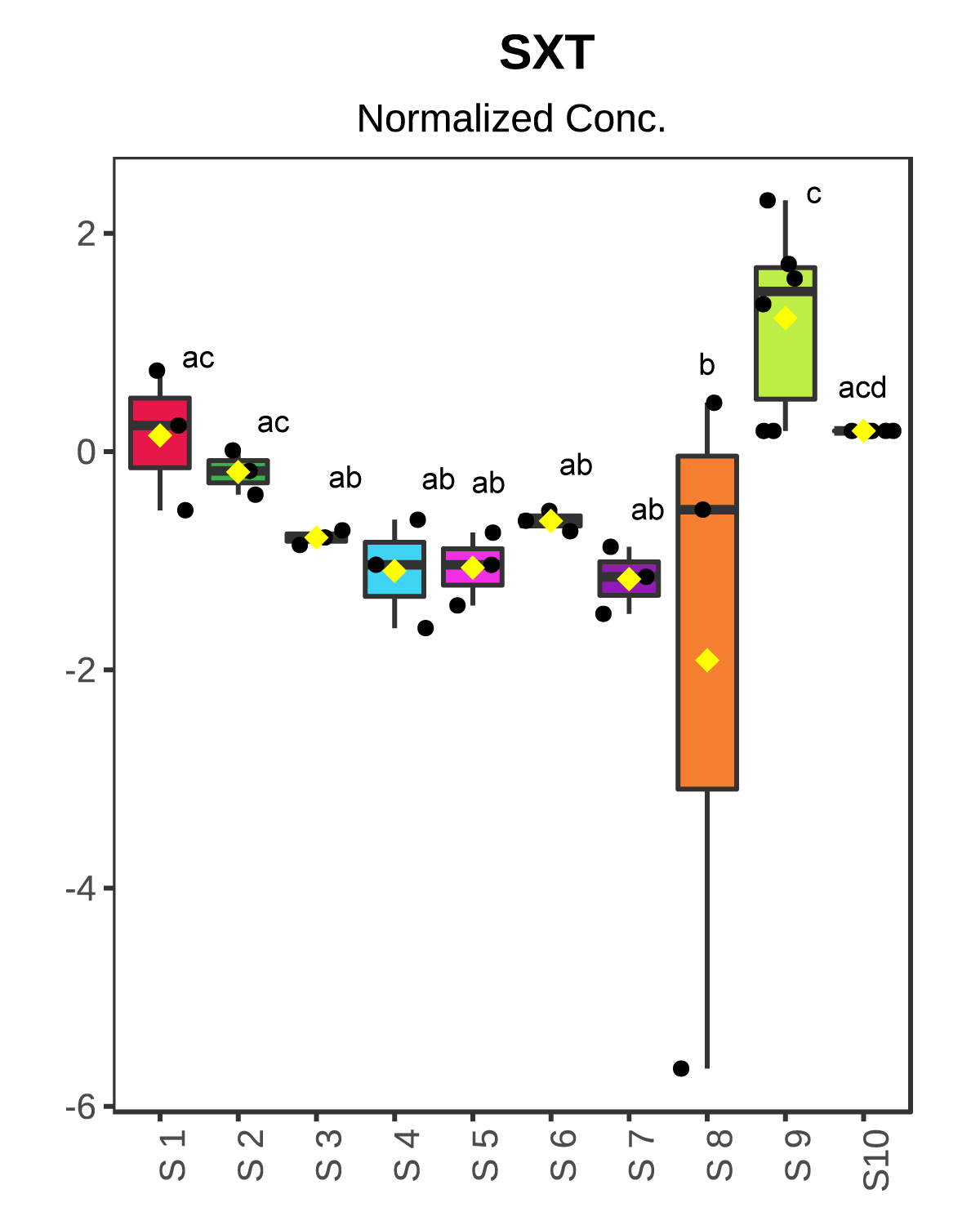


**B**


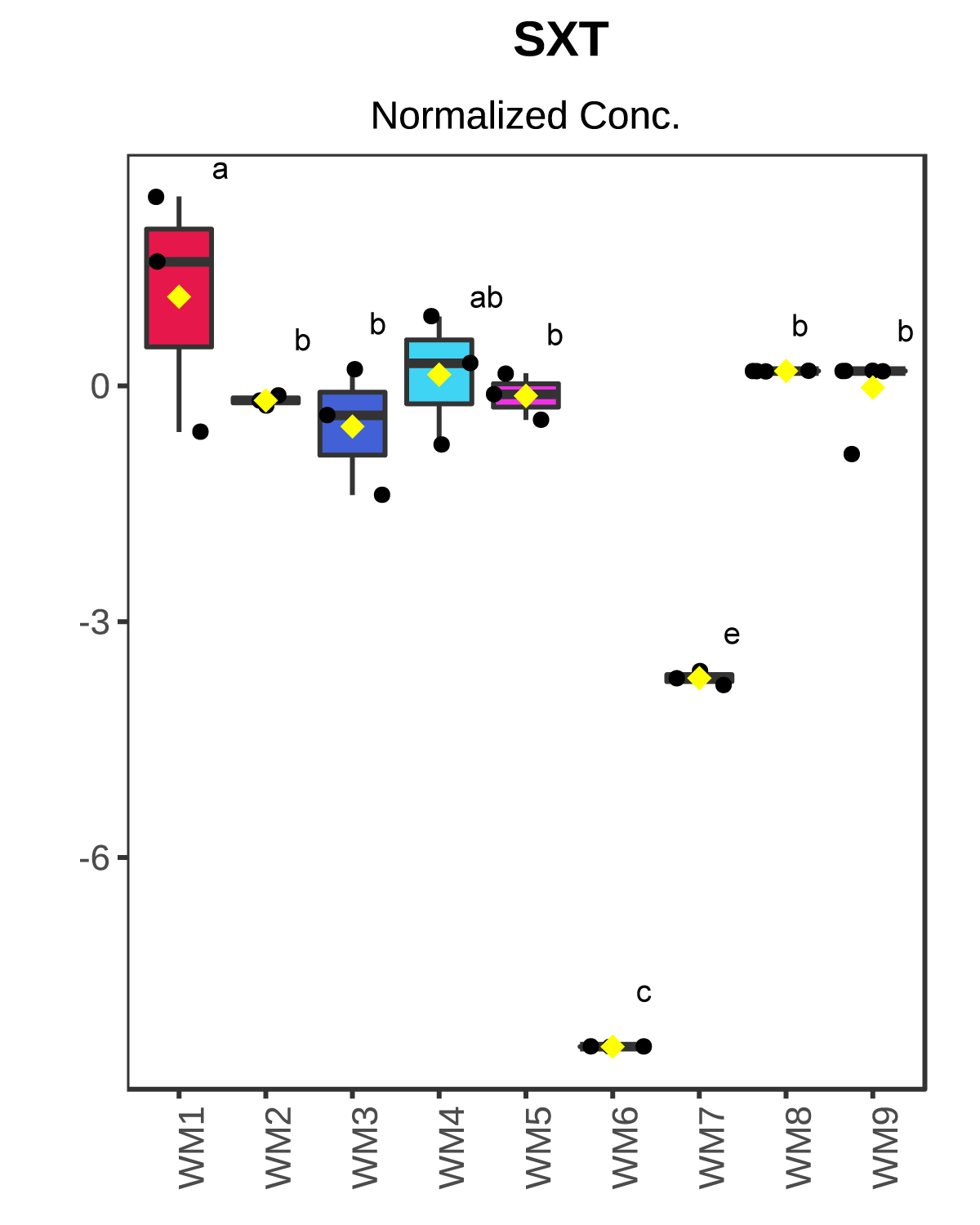


**A**

S9 Fig. Normalized concentration of sulfamethoxazole in samples from WM-WWTP (A) and S-WWTP (B). The graph shows the deviation from the average concentration of SXT. The data were normalized by dividing them by the mean concentration of all variables and cases (separately for S-WWTP and WM-WWTP). The letters stands for homogeneous groups, sites with same symbols indicate no statistical differences.


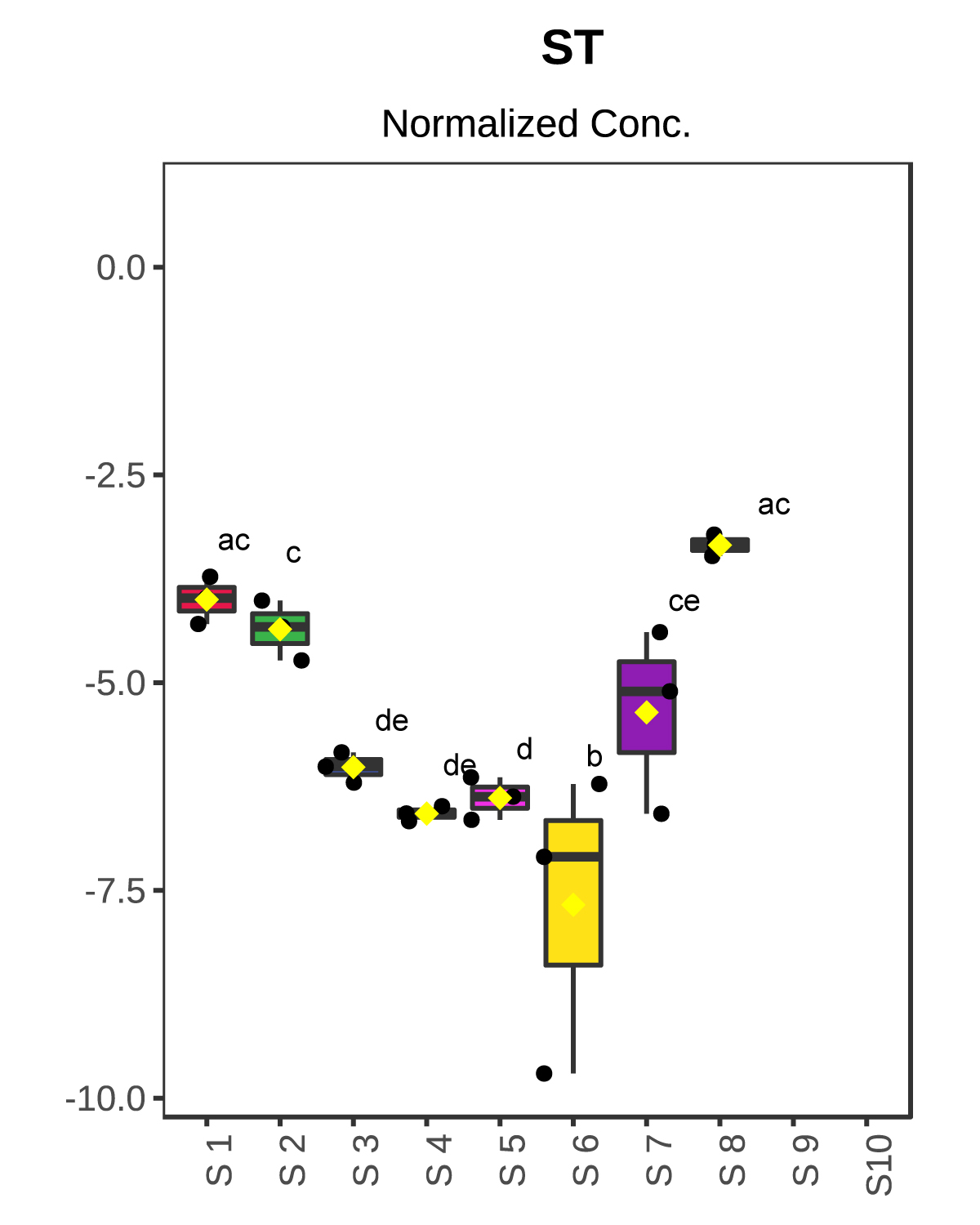


**B**


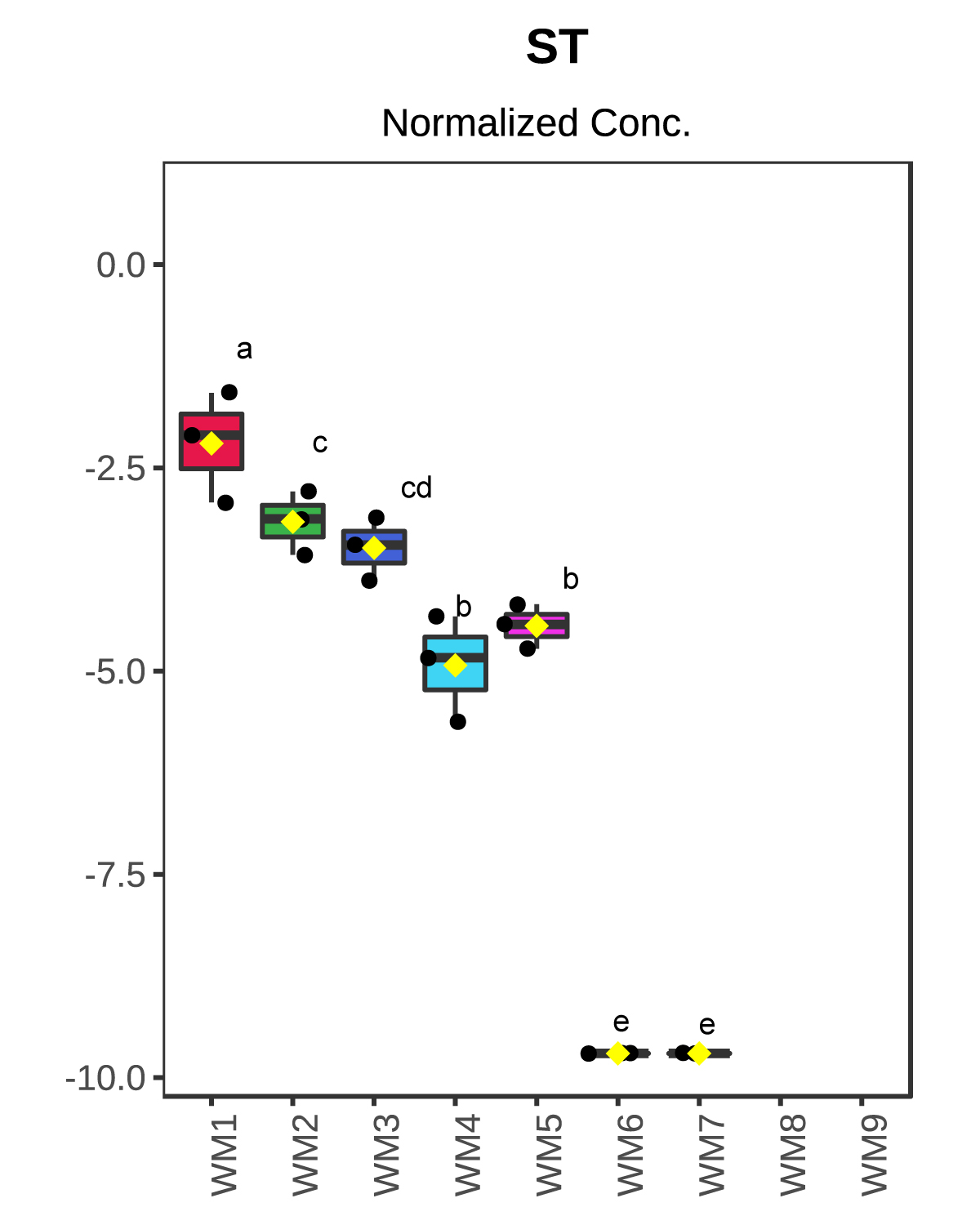


**A**

S10 Fig. Normalized concentration of sulfathiazole in samples from WM-WWTP (A) and S-WWTP (B). The graph shows the deviation from the average concentration of ST. The data were normalized by dividing them by the mean concentration of all variables and cases (separately for S-WWTP and WM-WWTP). The letters stands for homogeneous groups, sites with same symbols indicate no statistical differences.


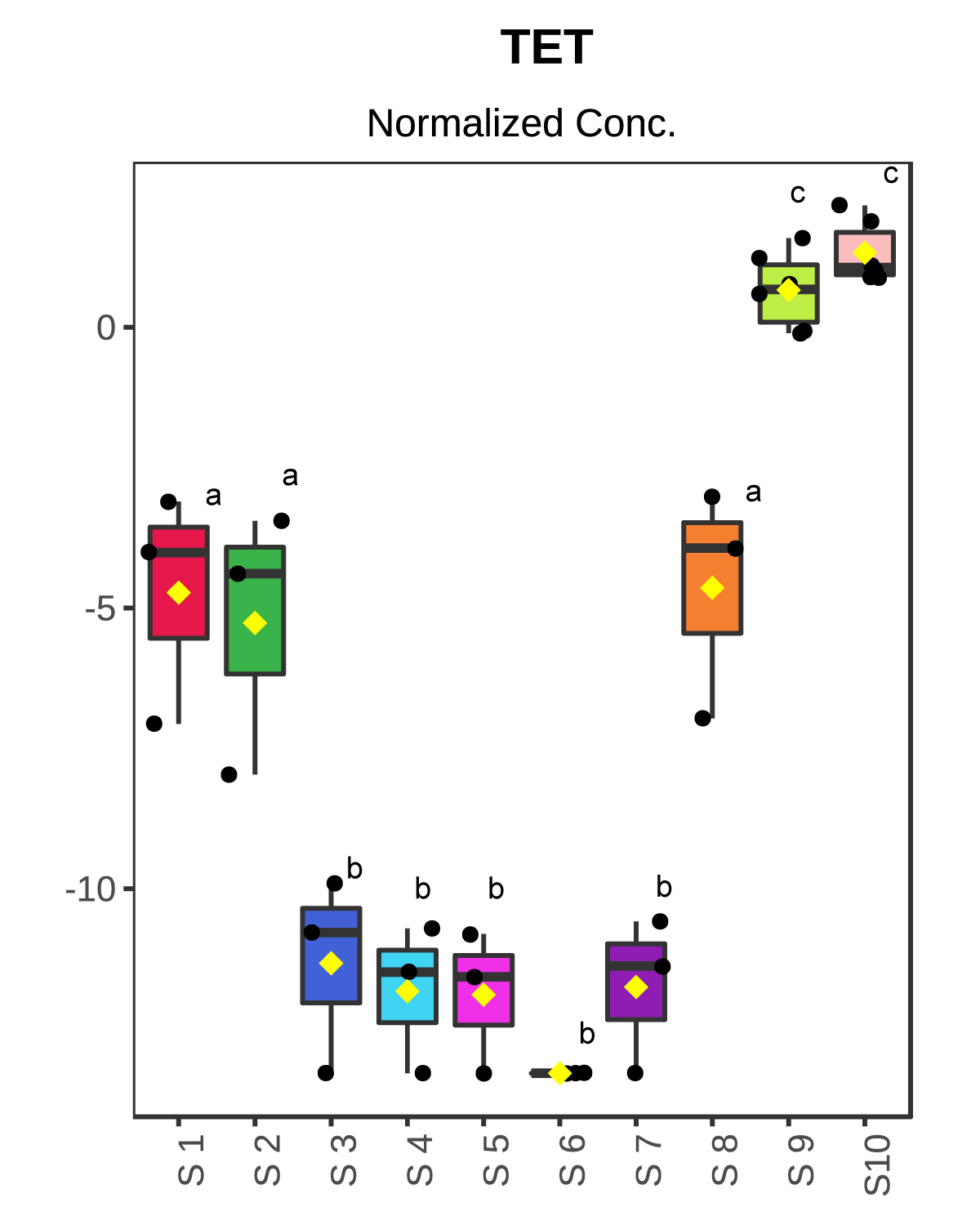


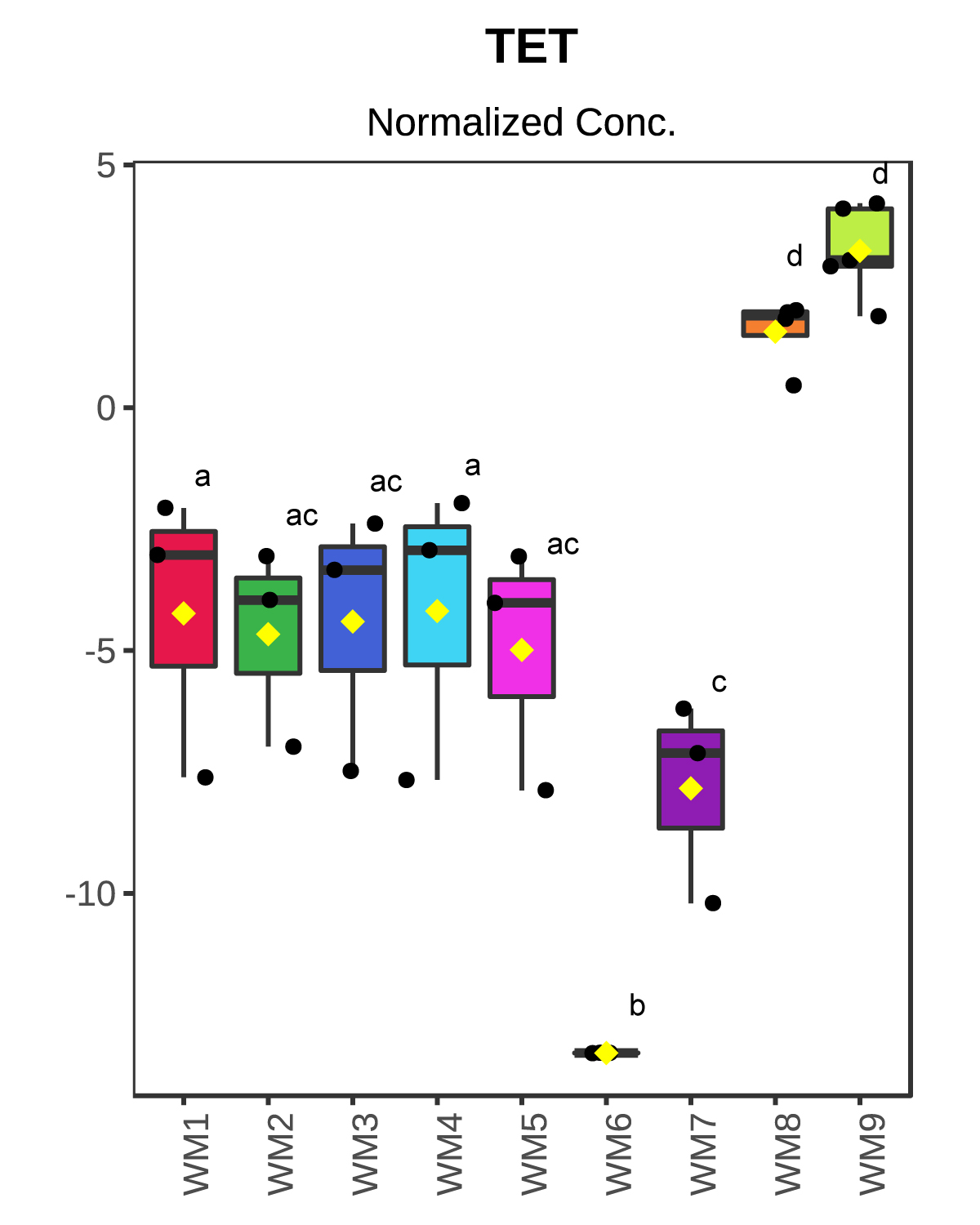


**A**

**B**

S11 Fig. Normalized concentration of tetracycline in samples from WM-WWTP (A) and S-WWTP (B). The graph shows the deviation from the average concentration of TET. The data were normalized by dividing them by the mean concentration of all variables and cases (separately for S-WWTP and WM-WWTP). The letters stands for homogeneous groups, sites with same symbols indicate no statistical differences.

**S3 Table Results of Mann-Whitney statistical test for significant differences of antibiotics concentrations between studied WWTPs.** Table showed values of significance (p) between WM-WWTP and S-WWTP, each antibiotic. Red marked samples are significant with p-value <0.05.

| Mann-Whitney U Test | | | | | |
| --- | --- | --- | --- | --- | --- |
| Antibiotics | Total ranks WM-WWTP | Total ranks S-WWTP | U | Z | p |
| ST | 94.0000 | 96.0000 | 41.00000 | 0.28577 | 0.775052 |
| SXT | 95.0000 | 95.0000 | 40.00000 | 0.36742 | 0.713303 |
| SDM | 104.0000 | 86.0000 | 31.00000 | 1.10227 | 0.270345 |
| TET | 113.5000 | 76.5000 | 21.50000 | 1.87794 | 0.060390 |
| OTC | 56.5000 | 133.5000 | 11.50000 | -2.69444 | 0.007051 |
| CIP | 102.0000 | 88.0000 | 33.00000 | 0.93897 | 0.347746 |
| NOR | 58.0000 | 132.0000 | 13.00000 | -2.57196 | 0.010113 |
| OFX | 94.0000 | 96.0000 | 41.00000 | 0.28577 | 0.775052 |
| PEF | 91.0000 | 99.0000 | 44.00000 | 0.04082 | 0.967436 |

S4 Table Values of transition for analyzed antibiotics. [m/z] – mass-to-charge ratio; DP – declustering potenctial; CE – collision energy.

| Antibiotic | [m/z] | DP [V] | CE [V] |
| --- | --- | --- | --- |
| CIP | 332>231 | 81 | 51 |
| CIP-D8 | 340>235 | 91 | 55 |
| NOR | 320>276 | 81 | 27 |
| OFX | 362>318 | 96 | 29 |
| OFX-D8 | 370>326 | 106 | 29 |
| OTC | 461>426 | 66 | 29 |
| PEF | 334>316 | 71 | 31 |
| SDM | 311>156 | 91 | 29 |
| SXT | 254>156 | 71 | 23 |
| SXT-D4 | 258>160 | 76 | 25 |
| ST | 256>156 | 71 | 23 |
| TET | 445>410 | 81 | 29 |
| TET-D6 | 451>416 | 86 | 27 |

**S5 Table Results of Mann-Whitney statistical test for significant differences of heavy metals concentrations between samples from WM-WWTP.** Table showed values of significance (p) between two chosen samples. Red marked samples are significant with p-value <0.05.

| WM-WWTP | Mann-Whitney U Test | | | | | | | | | |
| --- | --- | --- | --- | --- | --- | --- | --- | --- | --- | --- |
|  |  | WM1 | WM2 | WM3 | WM4 | WM5 | WM6 | WM7 | WM8 | WM9 |
|  | WM1 |  | 0.810 | 0.200 | 0.262 | 0.522 | 0.936 | 0.320 | 0.005 | 0.005 |
|  | WM2 | 0.810 |  | 0.200 | 0.200 | 0.689 | 0.749 | 1.000 | 0.005 | 0.005 |
|  | WM3 | 0.200 | 0.200 |  | 1.000 | 0.109 | 0.093 | 0.055 | 0.008 | 0.008 |
|  | WM4 | 0.262 | 0.200 | 1.000 |  | 0.109 | 0.093 | 0.055 | 0.008 | 0.008 |
|  | WM5 | 0.522 | 0.689 | 0.109 | 0.109 |  | 0.378 | 0.631 | 0.005 | 0.005 |
|  | WM6 | 0.936 | 0.749 | 0.093 | 0.093 | 0.378 |  | 0.631 | 0.005 | 0.005 |
|  | WM7 | 0.320 | 1.000 | 0.055 | 0.055 | 0.631 | 0.631 |  | 0.005 | 0.005 |
|  | WM8 | 0.005 | 0.005 | 0.008 | 0.008 | 0.005 | 0.005 | 0.005 |  | 0.575 |
|  | WM9 | 0.005 | 0.005 | 0.008 | 0.008 | 0.005 | 0.005 | 0.005 | 0.575 |  |

S6 Table Results of Mann-Whitney statistical test for significant differences of heavy metals concentrations between samples from S-WWTP. Table showed values of significance (p) between two chosen samples. Red marked samples are significant with p-value <0.05.

| S-WWTP | Mann-Whitney U Test | | | | | | | | | | |
| --- | --- | --- | --- | --- | --- | --- | --- | --- | --- | --- | --- |
|  |  | S1 | S2 | S3 | S4 | S5 | S6 | S7 | S8 | S9 | S10 |
|  | S1 |  | 0.631 | 0.423 | 0.423 | 0.631 | 0.927 | 0.584 | 0.055 | 0.008 | 0.008 |
|  | S2 | 0.631 |  | 0.689 | 0.575 | 0.936 | 0.810 | 0.471 | 0.045 | 0.005 | 0.005 |
|  | S3 | 0.423 | 0.689 |  | 0.936 | 0.810 | 0.575 | 0.810 | 0.008 | 0.005 | 0.005 |
|  | S4 | 0.423 | 0.575 | 0.936 |  | 0.749 | 0.749 | 0.873 | 0.020 | 0.005 | 0.005 |
|  | S5 | 0.631 | 0.936 | 0.810 | 0.749 |  | 0.810 | 0.522 | 0.008 | 0.005 | 0.005 |
|  | S6 | 0.927 | 0.810 | 0.575 | 0.749 | 0.810 |  | 0.378 | 0.008 | 0.005 | 0.005 |
|  | S7 | 0.584 | 0.471 | 0.810 | 0.873 | 0.522 | 0.378 |  | 0.008 | 0.005 | 0.005 |
|  | S8 | 0.055 | 0.045 | 0.008 | 0.020 | 0.008 | 0.008 | 0.008 |  | 0.008 | 0.008 |
|  | S9 | 0.008 | 0.005 | 0.005 | 0.005 | 0.005 | 0.005 | 0.005 | 0.008 |  | 0.810 |
|  | S10 | 0.008 | 0.005 | 0.005 | 0.005 | 0.005 | 0.005 | 0.005 | 0.008 | 0.810 |  |

**S7 Table Results of Mann-Whitney statistical test for significant differences of heavy metals between studied WWTPs.** Table showed values of significance (p) between WM-WWTP and S-WWTP, each gene. Red marked samples are significant with p-value <0.05.

| Mann-Whitney U Test | | | | | |
| --- | --- | --- | --- | --- | --- |
| Antibiotics | Total ranks WM-WWTP | Total ranks S-WWTP | U | Z | p |
| Zn | 96.0000 | 94.0000 | 39.00000 | 0.44907 | 0.653379 |
| Pb | 93.0000 | 97.0000 | 42.00000 | 0.20412 | 0.838257 |
| Ni | 75.0000 | 115.0000 | 30.00000 | -1.18392 | 0.236446 |
| Cr | 96.0000 | 94.0000 | 39.00000 | 0.44907 | 0.653379 |
| Co | 89.0000 | 101.0000 | 44.00000 | -0.04082 | 0.967436 |
| As | 104.0000 | 86.0000 | 31.00000 | 1.10227 | 0.270345 |

**S8 Table Average concentration of analyzed genes (copies/ml of wastewater or copies/g sewage sludge) and percentage reduction of genes after treatment process in samples from WM-WWTP, both seasons.**

| WM-WWTP | | | | | | | | | | | | | | | | | | | | | | | | | |  | | |  |
| --- | --- | --- | --- | --- | --- | --- | --- | --- | --- | --- | --- | --- | --- | --- | --- | --- | --- | --- | --- | --- | --- | --- | --- | --- | --- | --- | --- | --- | --- |
| Gene  Sampling site | | *bla*_TEM_ | | *bla*_OXA_ | | *bla*_SHV_ | | *tet*(A) | | *tet*(M) | | *qep*A | | aac(6’)-Ib-cr | | *sul*1 | | *sul*2 | | *int*I1 | | *int*I2 | | 16S rRNA | | | Mean | | |
| June | WM1 | | 1.08E+08 | | 1.07E+09 | | 1.07E+08 | | 1.04E+08 | | 1.31E+07 | | 1.66E+06 | | 7.70E+07 | | 4.13E+09 | | 9.01E+08 | | 1.70E+08 | | 4.98E+07 | | 4.90E+11 | | | 4.14E+10 |  |
|  | WM2 | | 2.77E+07 | | 3.64E+08 | | 4.43E+07 | | 1.69E+07 | | 2.47E+06 | | 1.38E+05 | | 1.20E+07 | | 8.55E+08 | | 1.76E+08 | | 3.77E+07 | | 6.56E+06 | | 8.45E+10 | | | 7.17E+09 |  |
|  | WM3 | | 4.54E+03 | | 3.08E+05 | | 3.99E+04 | | 1.13E+04 | | 1.53E+03 | | <LOD | | 1.26E+04 | | 1.31E+05 | | 6.88E+04 | | 6.88E+06 | | 1.71E+04 | | 7.04E+07 | | | 6.49E+06 |  |
|  | WM4 | | 4.98E+06 | | 2.01E+08 | | 8.65E+05 | | 8.03E+06 | | 1.76E+05 | | 1.23E+03 | | 6.09E+05 | | 2.35E+09 | | 9.48E+08 | | 6.13E+07 | | 8.89E+05 | | 1.26E+11 | | | 1.08E+10 |  |
|  | WM5 | | 5.50E+05 | | 7.03E+06 | | 5.10E+05 | | 4.96E+05 | | 1.45E+04 | | 3.01E+02 | | 1.63E+05 | | 1.27E+07 | | 3.29E+06 | | 2.12E+06 | | 5.72E+04 | | 2.11E+09 | | | 1.78E+08 |  |
|  | WM6 | | 5.55E+03 | | <LOD | | 4.06E+04 | | 2.93E+05 | | <LOD | | <LOD | | 9.38E+03 | | 8.64E+05 | | <LOD | | 1.38E+06 | | <LOD | | 1.94E+09 | | | 1.62E+08 |  |
|  | WM7 | | 6.90E+04 | | 1.42E+06 | | <LOD | | 4.95E+05 | | <LOD | | 5.30E+00 | | 4.52E+04 | | 3.96E+06 | | 2.19E+06 | | 2.77E+06 | | 1.99E+04 | | 3.41E+09 | | | 2.85E+08 |  |
|  | WM8 | | 9.34E+07 | | 1.10E+09 | | 1.15E+07 | | 2.24E+09 | | 2.55E+07 | | 0.00E+00 | | 3.04E+07 | | 4.80E+10 | | 1.22E+10 | | 2.56E+09 | | 1.24E+07 | | 9.88E+12 | | | 8.29E+11 |  |
|  | WM9 | | 1.69E+08 | | 1.41E+09 | | 2.53E+07 | | 3.62E+09 | | 1.04E+08 | | 1.26E+06 | | 8.66E+07 | | 6.39E+10 | | 3.16E+10 | | 2.45E+09 | | 2.80E+07 | | 1.91E+13 | | | 1.60E+12 |  |
| Reduction [%] | | 99.5 | | 99.3 | | 99.5 | | 99.5 | | 99.9 | | 100.0 | | 99.8 | | 99.7 | | 99.6 | | 98.8 | | 99.9 | | 99.6 | | | 99.6 | | |
| November | WM1 | | 2.03E+07 | | 1.24E+08 | | 1.57E+07 | | 8.20E+06 | | 1.86E+06 | | 4.89E+03 | | 1.18E+07 | | 2.05E+08 | | 8.69E+07 | | 2.69E+07 | | 1.17E+07 | | 1.13E+11 | | | 9.46E+09 |  |
|  | WM2 | | 1.68E+07 | | 1.26E+08 | | 1.29E+07 | | 6.61E+06 | | 1.08E+06 | | 2.10E+04 | | 5.51E+06 | | 1.31E+08 | | 6.68E+07 | | 1.80E+07 | | 5.58E+06 | | 5.48E+10 | | | 4.60E+09 |  |
|  | WM3 | | 4.43E+06 | | 1.59E+08 | | 2.15E+06 | | 8.02E+06 | | 6.66E+05 | | 3.20E+02 | | 2.38E+06 | | 2.77E+09 | | 7.89E+08 | | 5.04E+07 | | 1.59E+06 | | 1.95E+11 | | | 1.66E+10 |  |
|  | WM4 | | 1.12E+06 | | 4.17E+07 | | 2.94E+05 | | 2.85E+06 | | 3.88E+04 | | <LOD | | 2.04E+05 | | 3.50E+08 | | 1.28E+08 | | 1.03E+07 | | 1.64E+05 | | 4.73E+10 | | | 3.99E+09 |  |
|  | WM5 | | 2.19E+05 | | 5.31E+06 | | 6.88E+04 | | 1.91E+06 | | <LOD | | 4.64E+02 | | 1.00E+05 | | 1.39E+07 | | <LOD | | 1.61E+06 | | 2.38E+04 | | 1.97E+09 | | | 1.66E+08 |  |
|  | WM6 | | 5.77E+02 | | <LOD | | 7.98E+03 | | 8.71E+04 | | <LOD | | 0.00E+00 | | 4.15E+03 | | 1.43E+05 | | <LOD | | 1.33E+06 | | <LOD | | 4.29E+08 | | | 3.59E+07 |  |
|  | WM7 | | 2.40E+04 | | 6.39E+05 | | 1.72E+04 | | 1.81E+05 | | <LOD | | 4.55E+01 | | 1.58E+04 | | 1.51E+06 | | <LOD | | 6.95E+05 | | 7.40E+03 | | 8.01E+08 | | | 6.70E+07 |  |
|  | WM8 | | 8.71E+07 | | 1.41E+09 | | <LOD | | 1.20E+09 | | <LOD | | <LOD | | 2.79E+07 | | 3.57E+10 | | 1.40E+10 | | 1.99E+09 | | 1.49E+07 | | 5.42E+12 | | | 4.56E+11 |  |
|  | WM9 | | 9.87E+08 | | 2.53E+09 | | 6.55E+07 | | 8.86E+09 | | <LOD | | <LOD | | 4.99E+07 | | 5.20E+10 | | 5.23E+11 | | 3.25E+09 | | 5.51E+08 | | 2.25E+13 | | | 1.92E+12 |  |
| Reduction [%] | | 98.9 | | 95.7 | | 99.6 | | 76.7 | | 100.0 | | 90.5 | | 99.2 | | 93.2 | | 100.0 | | 94.0 | | 99.8 | | 98.3 | | | 95.5 | | |

S9 Table Average concentration of analyzed genes (copies/ml of wastewater or copies/g sewage sludge) and percentage reduction of genes after treatment process in samples from S-WWTP, both seasons.

| S-WWTP | | | | | | | | | | | | | | | | | | |  |
| --- | --- | --- | --- | --- | --- | --- | --- | --- | --- | --- | --- | --- | --- | --- | --- | --- | --- | --- | --- |
| Gene  Sampling site | | *bla*_TEM_ | *bla*_OXA_ | *bla*_SHV_ | *tet*(A) | *tet*(M) | *qep*A | aac(6’)-Ib-cr | | *sul*1 | | *sul*2 | | *int*I1 | | *int*I2 | | 16S rRNA | Mean |
| June | S1 | 3.09E+07 | 8.88E+07 | 3.66E+07 | 2.69E+08 | <LOD | 9.77E+05 | 1.86E+07 | 1.12E+09 | | 1.83E+09 | | 1.41E+08 | | 3.24E+07 | | 9.25E+11 | | 7.74E+10 |
|  | S2 | 2.94E+06 | 7.74E+06 | 5.24E+06 | 4.60E+07 | <LOD | <LOD | 8.11E+05 | 5.01E+07 | | 1.68E+08 | | 5.42E+06 | | 2.49E+06 | | 4.94E+10 | | 4.14E+09 |
|  | S3 | 7.13E+04 | 2.59E+05 | 5.81E+05 | <LOD | <LOD | <LOD | 6.12E+03 | 1.74E+06 | | 2.17E+06 | | 1.98E+05 | | 1.47E+04 | | 5.16E+08 | | 4.34E+07 |
|  | S4 | 1.54E+05 | 6.40E+05 | 7.69E+05 | 2.33E+06 | <LOD | <LOD | 4.39E+04 | 6.28E+06 | | 6.55E+06 | | 1.01E+06 | | 3.44E+04 | | 2.59E+09 | | 2.17E+08 |
|  | S5 | 8.47E+05 | 1.87E+06 | 4.99E+05 | 3.50E+06 | <LOD | <LOD | 1.69E+05 | 4.78E+07 | | 1.43E+07 | | 4.20E+06 | | 3.13E+04 | | 4.31E+09 | | 3.65E+08 |
|  | S6 | 9.71E+04 | 3.64E+05 | 9.75E+05 | <LOD | <LOD | <LOD | 1.74E+04 | 2.24E+06 | | <LOD | | 2.31E+05 | | 4.99E+04 | | 1.15E+09 | | 9.62E+07 |
|  | S7 | 1.04E+05 | 5.64E+05 | 4.64E+05 | <LOD | <LOD | <LOD | 3.06E+04 | 7.83E+06 | | <LOD | | 3.52E+05 | | 1.40E+04 | | 5.03E+09 | | 4.20E+08 |
|  | S8 | 2.39E+06 | 1.64E+07 | 1.49E+07 | <LOD | <LOD | <LOD | 1.08E+06 | 2.99E+08 | | <LOD | | 8.73E+06 | | 8.00E+05 | | 3.27E+11 | | 2.73E+10 |
|  | S9 | 3.24E+09 | 5.30E+09 | 2.01E+09 | 1.32E+10 | <LOD | 3.21E+07 | 1.76E+08 | 1.24E+11 | | 1.16E+11 | | 7.63E+09 | | 1.46E+08 | | 2.17E+13 | | 1.83E+12 |
|  | S10 | 5.09E+09 | 3.60E+10 | 2.13E+09 | 1.43E+10 | <LOD | 4.68E+08 | 2.70E+03 | 1.61E+11 | | 1.38E+11 | | 1.89E+10 | | 7.24E+09 | | 3.02E+13 | | 2.55E+12 |
| Reduction [%] | | 97.3 | 97.9 | 98.6 | 98.7 | - | 100.0 | 99.1 | | 95.7 | | 99.2 | | 97.0 | | 99.9 | | 99.5 | 98.5 |
| November | S1 | 3.36E+07 | 4.31E+08 | 1.31E+07 | 3.30E+08 | <LOD | 1.78E+07 | 3.58E+08 | 1.06E+09 | | 1.15E+09 | | 9.66E+07 | | 9.36E+06 | | 5.04E+11 | | 4.23E+10 |
|  | S2 | 1.97E+07 | 2.55E+08 | 2.06E+07 | 2.15E+08 | <LOD | 1.49E+06 | 1.09E+08 | 3.45E+08 | | 2.01E+09 | | 9.93E+07 | | 7.59E+06 | | 7.69E+11 | | 6.43E+10 |
|  | S3 | 1.14E+06 | 6.17E+06 | 4.52E+05 | 4.98E+06 | <LOD | 5.17E+04 | 1.43E+06 | 8.91E+06 | | <LOD | | 2.00E+06 | | 5.46E+04 | | 8.79E+09 | | 7.35E+08 |
|  | S4 | 7.40E+05 | 5.59E+06 | 2.72E+05 | 1.17E+07 | <LOD | <LOD | 1.60E+06 | 6.00E+06 | | <LOD | | 1.74E+06 | | 2.82E+04 | | 8.06E+09 | | 6.74E+08 |
|  | S5 | 1.58E+06 | 9.06E+06 | 5.90E+05 | 9.41E+06 | <LOD | 2.35E+04 | 1.45E+06 | 1.12E+07 | | <LOD | | 2.42E+06 | | 5.82E+04 | | 1.01E+10 | | 8.45E+08 |
|  | S6 | 3.00E+04 | 3.57E+05 | 1.89E+05 | 1.39E+06 | <LOD | <LOD | 8.56E+04 | 1.59E+06 | | 2.01E+07 | | 2.04E+05 | | 3.23E+04 | | 4.11E+09 | | 3.44E+08 |
|  | S7 | 4.99E+05 | 2.97E+06 | 9.52E+05 | 3.76E+06 | <LOD | <LOD | 9.64E+05 | 5.11E+06 | | 4.07E+07 | | 1.32E+06 | | 4.27E+04 | | 7.13E+09 | | 5.99E+08 |
|  | S8 | 1.52E+06 | 7.86E+06 | 4.73E+06 | <LOD | <LOD | <LOD | 1.34E+07 | 1.22E+08 | | 1.35E+09 | | 1.40E+07 | | 1.59E+07 | | 2.63E+11 | | 2.20E+10 |
|  | S9 | 1.80E+10 | 6.23E+10 | 4.63E+08 | 3.34E+10 | <LOD | 7.30E+06 | 2.52E+09 | 1.68E+11 | | 5.02E+11 | | 3.81E+10 | | 1.67E+08 | | 9.02E+13 | | 7.59E+12 |
|  | S10 | 1.37E+09 | 1.82E+10 | 7.73E+08 | 1.29E+10 | <LOD | 2.17E+08 | 1.65E+10 | 3.05E+10 | | 8.57E+10 | | 9.13E+09 | | 1.15E+09 | | 1.14E+13 | | 9.65E+11 |
| Reduction [%] | | 95.3 | 97.9 | 95.5 | 97.1 | - | 99.9 | 99.6 | | 98.9 | | 100.0 | | 97.5 | | 99.4 | | 98.0 | 98.1 |

S10 Table Average relative concentration of analyzed genes (copies/ml of wastewater or copies/g sewage sludge)/16S rRNA in samples from WM-WWTP, both seasons.

| WM-WWTP | | | | | | | | | | | | |  | |  |  |
| --- | --- | --- | --- | --- | --- | --- | --- | --- | --- | --- | --- | --- | --- | --- | --- | --- |
| Gene  Sampling site | | *bla*_TEM_ | *bla*_OXA_ | *bla*_SHV_ | *tet*(A) | *tet*(M) | *qep*A | aac(6’)-Ib-cr | *sul*1 | *sul*2 | *int*I1 | *int*I2 | | Mean | |  |
| June | P1 | 2.20E-04 | 2.20E-03 | 2.20E-04 | 2.13E-04 | 2.73E-05 | 3.39E-06 | 1.57E-04 | 8.64E-03 | 1.90E-03 | 3.51E-04 | 1.02E-04 | | 1.28E-03 | | |
|  | P2 | 3.30E-04 | 4.36E-03 | 5.30E-04 | 2.00E-04 | 2.91E-05 | 1.63E-06 | 1.44E-04 | 1.01E-02 | 2.06E-03 | 4.43E-04 | 7.81E-05 | | 1.66E-03 | | |
|  | P3 | 6.49E-05 | 4.44E-03 | 5.96E-04 | 1.62E-04 | 1.98E-05 | <LOD | 1.83E-04 | 1.85E-03 | 9.82E-04 | 9.84E-02 | 2.37E-04 | | 9.72E-03 | | |
|  | P4 | 4.09E-05 | 1.63E-03 | 7.08E-06 | 6.57E-05 | 1.40E-06 | 9.80E-09 | 5.49E-06 | 1.95E-02 | 7.34E-03 | 5.26E-04 | 6.45E-06 | | 2.64E-03 | | |
|  | P5 | 2.60E-04 | 3.26E-03 | 2.41E-04 | 2.36E-04 | 6.89E-06 | 1.44E-07 | 7.75E-05 | 6.04E-03 | 1.57E-03 | 1.01E-03 | 2.72E-05 | | 1.16E-03 | | |
|  | P6 | 2.87E-06 | <LOD | 2.12E-05 | 1.51E-04 | <LOD | <LOD | 4.84E-06 | 4.37E-04 | <LOD | 7.14E-04 | <LOD | | 1.21E-04 | | |
|  | P7 | 2.04E-05 | 4.20E-04 | <LOD | 1.47E-04 | <LOD | 1.80E-09 | 1.34E-05 | 1.18E-03 | 7.42E-04 | 8.21E-04 | 5.45E-06 | | 3.05E-04 | | |
|  | P8 | 9.76E-06 | 1.28E-04 | 1.20E-06 | 2.36E-04 | 2.67E-06 | <LOD | 3.30E-06 | 4.84E-03 | 1.20E-03 | 2.66E-04 | 1.29E-06 | | 6.08E-04 | | |
|  | P9 | 9.14E-06 | <LOD | 2.19E-06 | 1.95E-04 | 5.56E-06 | 7.09E-08 | 4.60E-06 | 3.38E-03 | 1.70E-03 | 1.32E-04 | 1.08E-06 | | 4.94E-04 | | |
| November | P1 | 1.88E-04 | 1.11E-03 | 1.41E-04 | 7.25E-05 | 1.67E-05 | 3.93E-08 | 1.07E-04 | 1.85E-03 | 7.88E-04 | 2.47E-04 | 1.10E-04 | | 4.21E-04 | | |
|  | P2 | 3.13E-04 | 2.34E-03 | 2.36E-04 | 1.24E-04 | 1.95E-05 | 3.83E-07 | 1.02E-04 | 2.40E-03 | 1.28E-03 | 3.32E-04 | 9.98E-05 | | 6.59E-04 | | |
|  | P3 | 2.29E-05 | 8.22E-04 | 1.10E-05 | 4.12E-05 | 3.52E-06 | 1.78E-09 | 1.22E-05 | 1.42E-02 | 4.17E-03 | 2.60E-04 | 8.11E-06 | | 1.78E-03 | | |
|  | P4 | 2.34E-05 | 8.93E-04 | 5.79E-06 | 6.14E-05 | 8.33E-07 | <LOD | 4.02E-06 | 7.50E-03 | 2.81E-03 | 2.20E-04 | 3.47E-06 | | 1.05E-03 | | |
|  | P5 | 1.19E-04 | 2.83E-03 | 3.63E-05 | 9.72E-04 | <LOD | 2.15E-07 | 5.48E-05 | 7.28E-03 | <LOD | 8.64E-04 | 1.32E-05 | | 1.11E-03 | | |
|  | P6 | 1.61E-06 | <LOD | 2.38E-05 | 2.32E-04 | <LOD | <LOD | 1.36E-05 | 3.62E-04 | <LOD | 3.55E-03 | <LOD | | 3.80E-04 | | |
|  | P7 | 3.02E-05 | 7.26E-04 | 2.20E-05 | 2.35E-04 | <LOD | 5.28E-08 | 2.04E-05 | 1.92E-03 | <LOD | 8.85E-04 | 9.50E-06 | | 3.50E-04 | | |
|  | P8 | 1.63E-05 | 2.62E-04 | <LOD | 2.21E-04 | <LOD | <LOD | 5.14E-06 | 6.45E-03 | 2.58E-03 | 3.70E-04 | 2.63E-06 | | 9.01E-04 | | |
|  | P9 | 4.38E-05 | 1.12E-04 | 2.94E-06 | 3.93E-04 | <LOD | <LOD | 2.22E-06 | 2.32E-03 | 2.32E-02 | 1.44E-04 | 2.43E-05 | | 2.38E-03 | | |

S11 Table Average relative concentration of analyzed genes (copies/ml of wastewater or copies/g sewage sludge)/16S rRNA in samples from S-WWTP, both seasons.

| S-WWTP | | | | | | | | | | | | |  |
| --- | --- | --- | --- | --- | --- | --- | --- | --- | --- | --- | --- | --- | --- |
| Gene  Sampling site | | *bla*_TEM_ | *bla*_OXA_ | *bla*_SHV_ | *tet*(A) | *tet*(M) | *qep*A | aac(6’)-Ib-cr | *sul*1 | *sul*2 | *int*I1 | *int*I2 | Mean |
| June | P1 | 3.36E-05 | 9.59E-05 | 3.97E-05 | 2.91E-04 | <LOD | 2.02E-05 | 1.22E-03 | 1.97E-03 | 1.52E-04 | 5.27E-05 | 3.36E-05 | 3.87E-04 |
|  | P2 | 5.92E-05 | 1.57E-04 | 1.06E-04 | 9.34E-04 | <LOD | 1.65E-05 | 1.02E-03 | 3.38E-03 | 1.09E-04 | 5.09E-05 | 5.92E-05 | 5.83E-04 |
|  | P3 | 1.40E-04 | 5.04E-04 | 1.12E-03 | 0.00E+00 | <LOD | 1.18E-05 | 3.37E-03 | 4.12E-03 | 3.90E-04 | 2.91E-05 | 1.40E-04 | 9.69E-04 |
|  | P4 | 6.00E-05 | 2.49E-04 | 2.99E-04 | 9.05E-04 | <LOD | 1.68E-05 | 2.46E-03 | 2.50E-03 | 3.94E-04 | 1.39E-05 | 6.00E-05 | 6.90E-04 |
|  | P5 | 1.97E-04 | 4.36E-04 | 1.16E-04 | 8.16E-04 | <LOD | 3.91E-05 | 1.11E-02 | 3.33E-03 | 6.35E-04 | 7.30E-06 | 1.97E-04 | 1.67E-03 |
|  | P6 | 8.53E-05 | 3.17E-04 | 8.52E-04 | <LOD | <LOD | 1.51E-05 | 1.96E-03 | <LOD | 2.03E-04 | 4.34E-05 | 8.53E-05 | 3.47E-04 |
|  | P7 | 2.08E-05 | 3.17E-04 | 9.25E-05 | <LOD | <LOD | 6.07E-06 | 1.57E-03 | <LOD | 7.07E-05 | 2.75E-06 | 2.08E-05 | 2.08E-04 |
|  | P8 | 7.26E-06 | 5.19E-05 | 4.87E-05 | <LOD | <LOD | 3.32E-06 | 9.63E-04 | <LOD | 2.72E-05 | 2.56E-06 | 7.26E-06 | 1.10E-04 |
|  | P9 | 1.49E-04 | 2.44E-04 | 9.24E-05 | 6.02E-04 | <LOD | 8.10E-06 | 5.73E-03 | 5.29E-03 | 3.51E-04 | 6.71E-06 | 1.49E-04 | 1.25E-03 |
|  | P10 | 1.70E-04 | 1.23E-03 | 7.13E-05 | 4.76E-04 | <LOD | 9.00E-11 | 5.38E-03 | 4.60E-03 | 6.29E-04 | 2.42E-04 | 1.70E-04 | 1.28E-03 |
| November | P1 | 6.64E-05 | 8.55E-04 | 2.61E-05 | 6.54E-04 | <LOD | 7.11E-04 | 2.09E-03 | 2.30E-03 | 2.86E-04 | 1.87E-05 | 6.64E-05 | 7.05E-04 |
|  | P2 | 2.57E-05 | 3.32E-04 | 2.72E-05 | 2.83E-04 | <LOD | 1.44E-04 | 4.53E-04 | 2.61E-03 | 1.32E-04 | 9.92E-06 | 2.57E-05 | 4.02E-04 |
|  | P3 | 1.31E-04 | 7.12E-04 | 5.07E-05 | 8.20E-04 | <LOD | 1.66E-04 | 1.00E-03 | <LOD | 2.28E-04 | 6.23E-06 | 1.31E-04 | 3.12E-04 |
|  | P4 | 9.19E-05 | 6.95E-04 | 3.37E-05 | 1.45E-03 | <LOD | 1.98E-04 | 7.53E-04 | <LOD | 2.19E-04 | 3.40E-06 | 9.19E-05 | 3.45E-04 |
|  | P5 | 1.56E-04 | 9.07E-04 | 5.81E-05 | 9.39E-04 | <LOD | 1.45E-04 | 1.11E-03 | <LOD | 2.40E-04 | 5.81E-06 | 1.56E-04 | 3.57E-04 |
|  | P6 | 1.06E-05 | 1.26E-04 | 6.65E-05 | 4.89E-04 | <LOD | 3.02E-05 | 5.20E-04 | 4.45E-03 | 6.55E-05 | 5.98E-06 | 1.06E-05 | 5.76E-04 |
|  | P7 | 7.04E-05 | 4.19E-04 | 1.35E-04 | 5.30E-04 | <LOD | 1.35E-04 | 7.21E-04 | 5.76E-03 | 1.84E-04 | 6.01E-06 | 7.04E-05 | 7.96E-04 |
|  | P8 | 5.78E-06 | 3.07E-05 | 2.01E-05 | <LOD | <LOD | 5.20E-05 | 4.66E-04 | 5.17E-03 | 5.35E-05 | 5.65E-05 | 5.78E-06 | 5.86E-04 |
|  | P9 | 2.00E-04 | 6.91E-04 | 5.14E-06 | 3.71E-04 | <LOD | 2.79E-05 | 1.87E-03 | 5.52E-03 | 4.24E-04 | 1.84E-06 | 2.00E-04 | 9.11E-04 |
|  | P10 | 1.20E-04 | 1.60E-03 | 6.81E-05 | 1.13E-03 | <LOD | 1.45E-03 | 2.69E-03 | 7.53E-03 | 8.03E-04 | 1.01E-04 | 1.20E-04 | 1.55E-03 |

**S12 Table Results of Kruskal-Wallis statistical test for significant differences of genes concentrations between samples in WM-WWTP.** Red marked samples are significant with p-value <0.05.

| WM-WWTP | Kruskal-Wallis test: H ( 8, N= 324) =130.5235 p =0.000 | | | | | | | | | |
| --- | --- | --- | --- | --- | --- | --- | --- | --- | --- | --- |
|  |  | WM1 | WM2 | WM3 | WM4 | WM5 | WM6 | WM7 | WM8 | WM9 |
|  | WM1 |  | 1.000 | 0.324 | 0.325 | 0.000 | 0.000 | 0.000 | 1.000 | 1.000 |
|  | WM2 | 1.000 |  | 1.000 | 1.000 | 0.028 | 0.000 | 0.000 | 1.000 | 0.257 |
|  | WM3 | 0.324 | 1.000 |  | 1.000 | 1.000 | 0.001 | 0.042 | 0.296 | 0.002 |
|  | WM4 | 0.325 | 1.000 | 1.000 |  | 1.000 | 0.000 | 0.041 | 0.297 | 0.002 |
|  | WM5 | 0.000 | 0.028 | 1.000 | 1.000 |  | 0.725 | 1.000 | 0.000 | 0.000 |
|  | WM6 | 0.000 | 0.000 | 0.001 | 0.000 | 0.725 |  | 1.000 | 0.000 | 0.000 |
|  | WM7 | 0.000 | 0.000 | 0.042 | 0.041 | 1.000 | 1.000 |  | 0.000 | 0.000 |
|  | WM8 | 1.000 | 1.000 | 0.296 | 0.297 | 0.000 | 0.000 | 0.000 |  | 1.000 |
|  | WM9 | 1.000 | 0.257 | 0.002 | 0.002 | 0.000 | 0.000 | 0.000 | 1.000 |  |

**S13 Table Results of Kruskal-Wallis statistical test for significant differences of genes concentrations between samples in S-WWTP.** Red marked samples are significant with p-value <0.05.

| S-WWTP | Kruskal-Wallis test: H ( 9, N= 330) =177.4590 p =0.000 | | | | | | | | | | | |
| --- | --- | --- | --- | --- | --- | --- | --- | --- | --- | --- | --- | --- |
|  |  | S1 | S2 | S3 | S4 | S5 | S6 | S7 | S8 | S9 | S10 |  |
|  | S1 |  | 1.000 | 0.000 | 0.000 | 0.002 | 0.000 | 0.000 | 0.343 | 1.000 | 0.362 |  |
|  | S2 | 1.000 |  | 0.001 | 0.009 | 0.098 | 0.000 | 0.004 | 1.000 | 0.072 | 0.013 |  |
|  | S3 | 0.000 | 0.001 |  | 1.000 | 1.000 | 1.000 | 1.000 | 0.547 | 0.000 | 0.000 |  |
|  | S4 | 0.000 | 0.009 | 1.000 |  | 1.000 | 1.000 | 1.000 | 1.000 | 0.000 | 0.000 |  |
|  | S5 | 0.002 | 0.098 | 1.000 | 1.000 |  | 1.000 | 1.000 | 1.000 | 0.000 | 0.000 |  |
|  | S6 | 0.000 | 0.000 | 1.000 | 1.000 | 1.000 |  | 1.000 | 0.045 | 0.000 | 0.000 |  |
|  | S7 | 0.000 | 0.004 | 1.000 | 1.000 | 1.000 | 1.000 |  | 1.000 | 0.000 | 0.000 |  |
|  | S8 | 0.343 | 1.000 | 0.547 | 1.000 | 1.000 | 0.045 | 1.000 |  | 0.000 | 0.000 |  |
|  | S9 | 1.000 | 0.072 | 0.000 | 0.000 | 0.000 | 0.000 | 0.000 | 0.000 |  | 1.000 |  |
|  | S10 | 0.362 | 0.013 | 0.000 | 0.000 | 0.000 | 0.000 | 0.000 | 0.000 | 1.000 |  |  |

**S14 Table Results of Mann-Whitney statistical test for significant differences of genes concentrations between analyzed WWTPs.** Table showed values of significance (p) between WM-WWTP and S-WWTP, each gene. Red marked samples are significant with p-value <0.05.

| Mann-Whitney U Test | | | | | |
| --- | --- | --- | --- | --- | --- |
| Antibiotics | Total ranks WM-WWTP | Total ranks S-WWTP | U | Z | p |
| *bla*TEM | 84.00000 | 87.00000 | 39.00000 | -0.08830 | 0.929637 |
| *bla*OXA | 88.00000 | 83.00000 | 38.00000 | 0.17660 | 0.859819 |
| *bla*SHV | 79.00000 | 92.00000 | 34.00000 | -0.52981 | 0.596242 |
| *tet*(A) | 76.00000 | 95.00000 | 31.00000 | -0.79472 | 0.426777 |
| *qep*A | 76.00000 | 95.00000 | 31.00000 | -0.79472 | 0.426777 |
| *aac*(6`)-*Ib*-*cr* | 73.00000 | 98.00000 | 28.00000 | -1.05963 | 0.289316 |
| *sul*1 | 91.00000 | 80.00000 | 35.00000 | 0.44151 | 0.658844 |
| *sul*2 | 82.00000 | 89.00000 | 37.00000 | -0.26491 | 0.791082 |
| *int*I1 | 91.00000 | 80.00000 | 35.00000 | 0.44151 | 0.658844 |
| *int*I2 | 85.00000 | 86.00000 | 40.00000 | 0.00000 | 1.000000 |


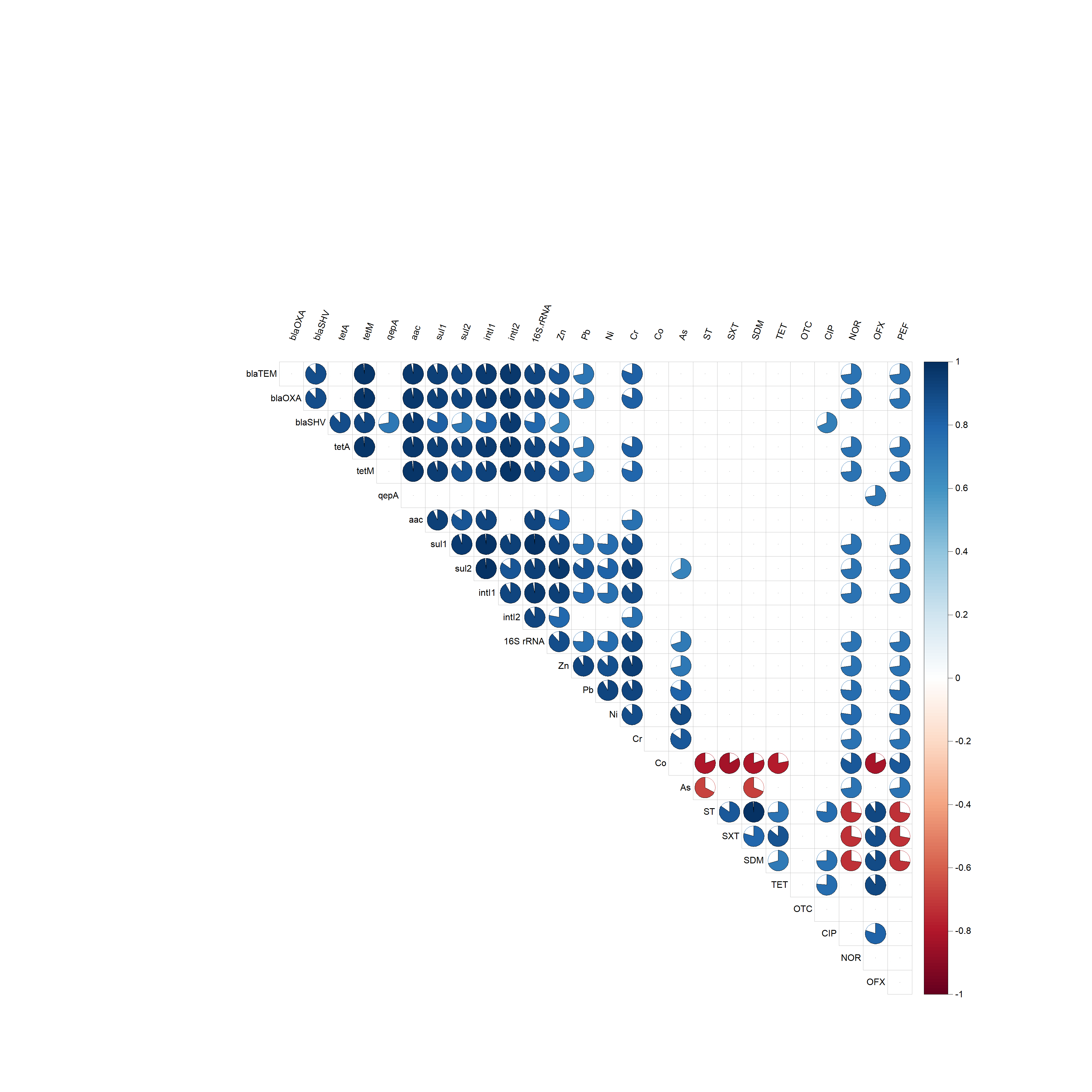


S12 Fig. Correlation between concentration of ARGs (copies/mL), integrase genes (copies/mL), heavy metals and antibiotics in samples from WM-WWTP. Positive correlations are displayed in blue and negative in red color. Color intensity and the pie chart are proportional to the correlation coefficients.


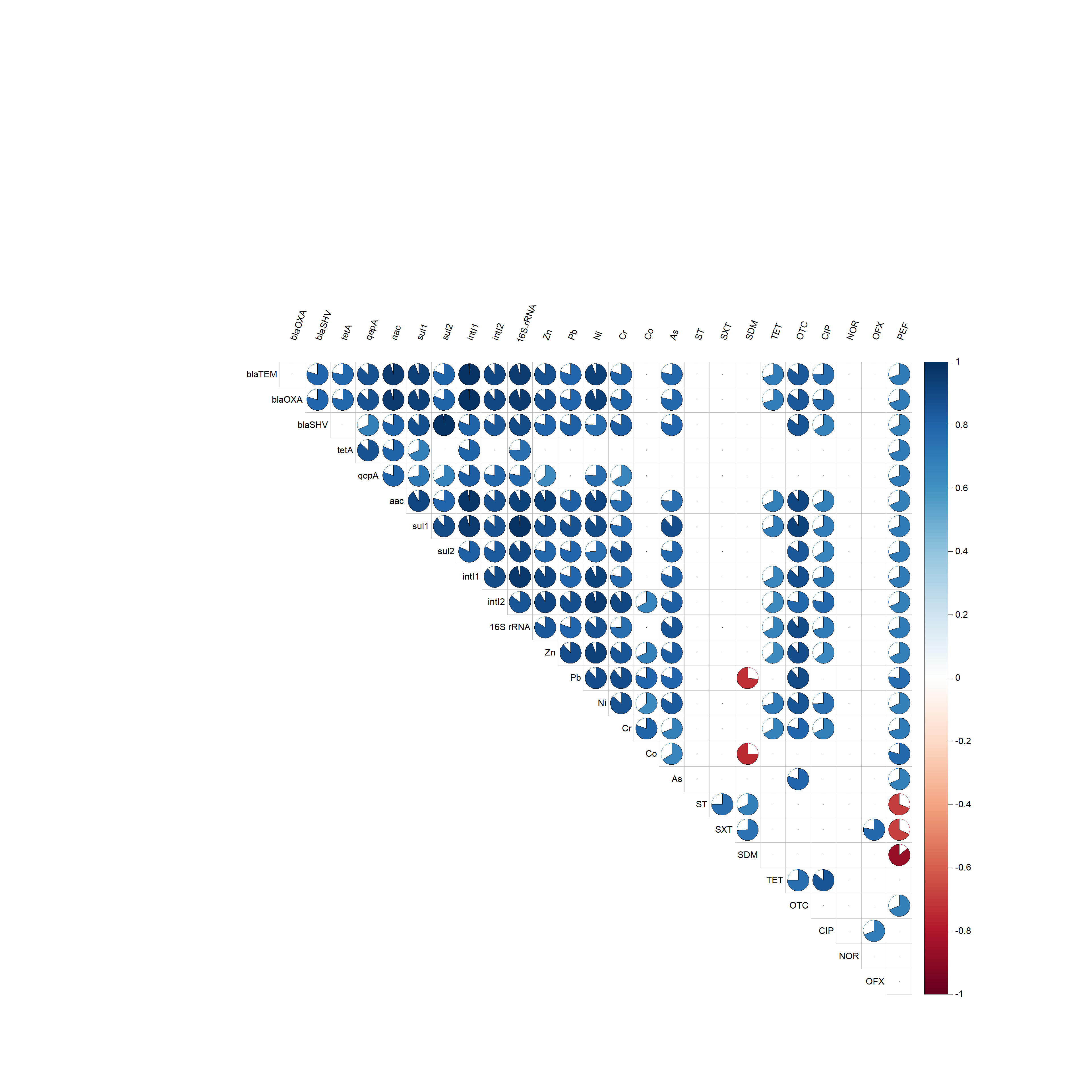


S13 Fig. Correlation between concentration of ARGs (copies/mL), integrase genes (copies/mL), heavy metals and antibiotics in samples from S-WWTP. Positive correlations are displayed in blue and negative in red color. Color intensity and the pie chart are proportional to the correlation coefficients.
