# Supplementary material for: Industrialization as a source of heavy metals and antibiotics which can enhance the antibiotic resistance in wastewater, sewage sludge and river water
Source: PLoS One. 2021 Jun 4;16(6):e0252691. doi: 10.1371/journal.pone.0252691 (PMC8177550; doi:10.1371/journal.pone.0252691)
Supplement: Attachment — Submitted filename: PONE-D-21-02977_reviewer_ME.pdf [file pone.0252691.s002.pdf]

PLOS ONE

Industrialization as a Factor Enhancing Antibiotic Resistance in Wastewater, Sewage Sludge and River Water

--Manuscript Draft--

**Manuscript Number:**	PONE-D-21-02977
**Article Type:**	Research Article
**Full Title:**	Industrialization as a Factor Enhancing Antibiotic Resistance in Wastewater, Sewage Sludge and River Water
**Short Title:**	Industrialization enhancing Antibiotic Resistance in Environment
**Corresponding Author:**	Monika Harnisz<br>University of Warmia and Mazury in Olsztyn: Uniwersytet Warminsko-Mazurski w Olsztynie<br>Olsztyn, POLAND
**Keywords:**	wastewater; wastewater treatment plant; selective pressure; antibiotic resistance genes; heavy metals; antibiotics
**Abstract:**	The spread of antibiotic resistance is closely related with selective pressure in the environment. Wastewater from industrialized regions is characterized by higher concentrations of these pollutants than sewage from less industrialized areas. The aim of this study was to compare the concentrations of contaminants such as antibiotics and heavy metals (HMs), and to evaluate their impact on the spread of genes encoding resistance to antimicrobial drugs in samples of wastewater, sewage sludge and river water in two regions with different levels of industrialization. The factors exerting selective pressure, which significantly contributed to the occurrence of the examined antibiotic resistance genes (ARGs), were identified. The concentrations of selected genes conferring resistance to four groups of antibiotics as well as class 1 and 2 integron-integrase genes were determined in the analyzed samples. The concentrations of six HMs and antibiotics from 3 groups were determined. Very few significant correlations were noted between the concentrations of antibiotics and ARGs in the examined samples. In turn, HM levels were correlated with ARG concentrations, which confirms the important role of HMs in promoting drug resistance. The samples from a wastewater treatment plant (WWTP) located in industrialized region were characterized by higher HM contamination and a higher number of significant correlations between the analyzed variables than the samples collected from a WWTP located in a weakly industrialized region. These results indicate that treated wastewater evacuated to the natural environment can pose a continuous threat to human health by transferring ARGs, antibiotics and HMs to the environment. The findings provides shed light on impact of industrialization on antibiotic resistance dissemination issue.
**Order of Authors:**	Jakub Hubeny
	Monika Harnisz
	Ewa Korzeniewska
	Martyna Buta
	Wiktor Zieliński
	Damian Rolbiecki
	Joanna Giebułtowicz
	Grzegorz Nałęcz-Jawecki
	Grażyna Płaza
**Additional Information:**	
**Question**	**Response**
**Financial Disclosure**	This study was supported by grant No. 2017/M/NZ9/00071 from the Polish National Science Centre. Jakub Hubeny has received a scholarship from the Interdisciplinary

Powered by Editorial Manager® and ProduXion Manager® from Aries Systems Corporation

Enter a financial disclosure statement that describes the sources of funding for the work included in this submission. Review the submission guidelines for detailed requirements. View published research articles from *PLOS ONE* for specific examples.<br><br>This statement is required for submission and **will appear in the published article** if the submission is accepted. Please make sure it is accurate.<br><br>**Unfunded studies**<br>Enter: *The author(s) received no specific funding for this work.*<br><br>**Funded studies**<br>Enter a statement with the following details:<br>• Initials of the authors who received each award<br>• Grant numbers awarded to each author<br>• The full name of each funder<br>• URL of each funder website<br>• Did the sponsors or funders play any role in the study design, data collection and analysis, decision to publish, or preparation of the manuscript?<br>• **NO** - Include this sentence at the end of your statement: *The funders had no role in study design, data collection and analysis, decision to publish, or preparation of the manuscript.*<br>• **YES** - Specify the role(s) played.<br><br>* typeset	Doctoral Program in Bioeconomy (POWR.03.02.00-00-I034/16 00) funded by the European Social Fund.
**Competing Interests**<br><br>Use the instructions below to enter a competing interest statement for this submission. On behalf of all authors, disclose any competing interests that could be perceived to bias this work—acknowledging all financial support and any other relevant financial or non-financial competing interests.<br><br>This statement is required for submission and **will appear in the published article** if the submission is accepted. Please make	The authors have declared that no competing interests exist.

Powered by Editorial Manager® and ProduXion Manager® from Aries Systems Corporation

sure it is accurate and that any funding sources listed in your Funding Information later in the submission form are also declared in your Financial Disclosure statement.<br><br>View published research articles from *PLOS ONE* for specific examples.<br><br>**NO authors have competing interests**<br><br>Enter: *The authors have declared that no competing interests exist.*<br><br>**Authors with competing interests**<br><br>Enter competing interest details beginning with this statement:<br><br>*I have read the journal's policy and the authors of this manuscript have the following competing interests: [insert competing interests here]*<br><br>* typeset	
**Ethics Statement**<br><br>Enter an ethics statement for this submission. This statement is required if the study involved:<br><br>• Human participants<br>• Human specimens or tissue<br>• Vertebrate animals or cephalopods<br>• Vertebrate embryos or tissues<br>• Field research<br><br>Write "N/A" if the submission does not require an ethics statement.<br><br>General guidance is provided below. Consult the submission guidelines for detailed instructions. **Make sure that all information entered here is included in the Methods section of the manuscript.**	N/A

**Format for specific study types**<br><br>**Human Subject Research (involving human participants and/or tissue)**<br>• Give the name of the institutional review board or ethics committee that approved the study<br>• Include the approval number and/or a statement indicating approval of this research<br>• Indicate the form of consent obtained (written/oral) or the reason that consent was not obtained (e.g. the data were analyzed anonymously)<br><br>**Animal Research (involving vertebrate animals, embryos or tissues)**<br>• Provide the name of the Institutional Animal Care and Use Committee (IACUC) or other relevant ethics board that reviewed the study protocol, and indicate whether they approved this research or granted a formal waiver of ethical approval<br>• Include an approval number if one was obtained<br>• If the study involved *non-human primates*, add *additional details* about animal welfare and steps taken to ameliorate suffering<br>• If anesthesia, euthanasia, or any kind of animal sacrifice is part of the study, include briefly which substances and/or methods were applied<br><br>**Field Research**<br><br>Include the following details if this study involves the collection of plant, animal, or other materials from a natural setting:<br>• Field permit number<br>• Name of the institution or relevant body that granted permission	
**Data Availability**<br><br>Authors are required to make all data underlying the findings described fully available, without restriction, and from the time of publication. PLOS allows rare exceptions to address legal and ethical concerns. See the PLOS Data Policy and FAQ for detailed information.	Yes - all data are fully available without restriction

A Data Availability Statement describing where the data can be found is required at submission. Your answers to this question constitute the Data Availability Statement and **will be published in the article**, if accepted.<br><br>**Important:** Stating 'data available on request from the author' is not sufficient. If your data are only available upon request, select 'No' for the first question and explain your exceptional situation in the text box.<br><br>Do the authors confirm that all data underlying the findings described in their manuscript are fully available without restriction?	
**Describe where the data may be found in full sentences. If you are copying our sample text, replace any instances of XXX with the appropriate details.**<br><br>• If the data are **held or will be held in a public repository**, include URLs, accession numbers or DOIs. If this information will only be available after acceptance, indicate this by ticking the box below. For example: *All XXX files are available from the XXX database (accession number(s) XXX, XXX.).*<br>• If the data are all contained **within the manuscript and/or Supporting Information files**, enter the following: *All relevant data are within the manuscript and its Supporting Information files.*<br>• If neither of these applies but you are able to provide **details of access elsewhere**, with or without limitations, please do so. For example:<br><br>*Data cannot be shared publicly because of [XXX]. Data are available from the XXX Institutional Data Access / Ethics Committee (contact via XXX) for researchers who meet the criteria for access to confidential data.*<br><br>*The data underlying the results presented in the study are available from (include the name of the third party*	All relevant data are within the manuscript and its Supporting Information files.

and contact information or URL).<br>• This text is appropriate if the data are owned by a third party and authors do not have permission to share the data.<br><br>* typeset	
Additional data availability information:	

Manuscript
Click here to access/download;Manuscript;Hubeny et al. PLOS ONE .docx

Industrialization as a Factor Enhancing Antibiotic Resistance in Wastewater, Sewage Sludge and River Water

Jakub Hubeny [1], Monika Harnisz [1*], Ewa Korzeniewska [1], Martyna Buta [1], Wiktor Zieliński [1], Damian Rolbiecki [1], Joanna Giebułtowicz [2], Grzegorz Nałęcz-Jawecki [3], Grażyna Płaza [4]

Author affiliations:

[1] Department of Water Protection Engineering and Environmental Microbiology, Faculty of Geoengineering, University of Warmia and Mazury in Olsztyn, Prawocheńskiego 1, 10-720 Olsztyn, Poland

[2] Department of Bioanalysis and Drug Analysis, Faculty of Pharmacy, Medical University of Warsaw, Banacha 1, 02-097 Warszawa, Poland

[3] Department of Environmental Health Sciences, Faculty of Pharmacy, Medical University of Warsaw, Banacha 1, 02-097 Warszawa, Poland

[4] Microbiology Unit, Institute for Ecology of Industrial Areas, Kossutha 6, 40-844 Katowice, Poland

* Corresponding author

E-mail: monika.harnisz@uwm.edu.pl; monikah@uwm.edu.pl

Abstract

The spread of antibiotic resistance is closely related with selective pressure in the environment. Wastewater from industrialized regions is characterized by higher concentrations of these pollutants than sewage from less industrialized areas. The aim of this study was to compare the concentrations of contaminants such as antibiotics and heavy metals (HMs), and to evaluate their impact on the spread of genes encoding resistance to antimicrobial drugs in samples of wastewater, sewage sludge and river water in two regions with different levels of industrialization. The factors exerting selective pressure, which significantly contributed to the occurrence of the examined antibiotic resistance genes (ARGs), were identified. The concentrations of selected genes conferring resistance to four groups of antibiotics as well as class 1 and 2 integron-integrase genes were determined in the analyzed samples. The concentrations of six HMs and antibiotics from 3 groups were determined. Very few significant correlations were noted between the concentrations of antibiotics and ARGs in the examined samples. In turn, HM levels were correlated with ARG concentrations, which confirms the important role of HMs in promoting drug resistance. The samples from a wastewater treatment plant (WWTP) located in industrialized region were characterized by higher HM contamination and a higher number of significant correlations between the analyzed variables than the samples collected from a WWTP located in a weakly industrialized region. These results indicate that treated wastewater evacuated to the natural environment can pose a continuous threat to human health by transferring ARGs, antibiotics and HMs to the environment. The findings ~~provides~~ shed light on impact of industrialization on antibiotic resistance dissemination issue.

Keywords

wastewater, wastewater treatment plant, selective pressure, antibiotic resistance genes, heavy metals, antibiotics

Introduction

Industrial development is the leading cause of environmental pollution with various substances, including heavy metals (HMs), antibiotics and pesticides [1,2]. Highly urbanized and industrialized regions are characterized by higher levels of industrial production and higher population density than regions with extensive forest cover where tourism and recreation are the predominant income-generating activities [3,4]. Land-use type can significantly influence the composition of wastewater reaching wastewater treatment plants (WWTPs). In highly industrialized areas, wastewater is composed of municipal, industrial and hospital wastewater, whereas in less industrialized regions, industrial wastewater has a smaller share of the sewage mix that reaches WWTPs. As a result, wastewater from industrialized regions is characterized by higher concentrations of HMs than sewage generated in less industrialized areas [5,6].

Treated wastewater released into the environment is less abundant in HMs. Heavy metals are strongly immobilized on solid particles, and they are accumulated in sewage sludge during wastewater treatment. According to the European Commission, 11.5 million tons of sludge dry solids were generated in the European Union in 2010 during wastewater treatment, and this value is expected to increase to 13 million tons in 2020 [7]. Wastewater treatment plants are not equipped with specialized systems for eliminating HMs; therefore, the removal of HMs through accumulation in sewage sludge can be regarded as a by-product of the treatment process. The use of sewage sludge in agriculture has become a cause for concern because the strong absorption of HMs to sewage sludge can pose a serious threat for the environment and public health [8]. The suitability of sewage sludge for agriculture is determined by its HM content which is analyzed and controlled based on the standards set forth by the European Council Directive 86/278/EEC [9]. The proportions of sewage sludge used in agricultural production vary across European countries. In Sweden, Poland and Germany, around 25% of

the generated sewage sludge is applied in the farming sector, whereas in Spain, France and Great Britain, this figure can be as high as 70% [7].

Antibiotics are widely used in both highly industrialized and developing countries, and they are detected in wastewater [10], soil [11] and surface waters [12]. The processes of industrialization and urbanization are followed by environmental contamination with emergence pollutants as antibiotics [13]. Further development of countries is linked with release many contaminants to the wastewater and the aquatic environment, due to industrial activities, population growth and agricultural [14]. According to the European Center for Disease Prevention and Control (ECDC), Poland belongs to the group of six European countries with the highest levels of antibiotic consumption per capita [15]. Excessive and inappropriate use of antibiotics is the main cause of antibiotic resistance around the world [16]. Antibiotics reach WWTPs with human and animal feces, incorrectly disposed unused drugs, and industrial wastewater. Their concentrations in wastewater influents and effluents range from ng/l to ug/l worldwide, which increases the risk of selective resistance [17–21]. Antibiotics are degraded by hydrolysis and photodegradation, and they are adsorbed on the surface of sludge particles, which decreases antimicrobial concentrations in wastewater [22]. Numerous research studies have demonstrated that the processing technologies deployed by WWTPs do not effectively remove antibiotics from untreated wastewater [23–25].

Wastewater treatment plants are hotspots of antibiotic resistance [26], and they are regarded as direct sources of the spread of antibiotic resistance in the environment [27]. Wastewater flowing into WWTPs contains antibiotics, HMs, antibiotic-resistant bacteria (ARB) and antibiotic resistance genes (ARGs) from hospitals, households and industrial plants. Wastewater treatment systems are designed to remove organic compounds and solid suspensions containing bacterial cells, but there are not effective technologies for eliminating genetic material, including mobile genetic elements (MGEs) [20,28].

There is a growing body of evidence to indicate that the copy numbers of ARGs can be high in wastewater with low levels of antibiotic pollution or that the concentrations of these micropollutants are not bound by significant correlations [29,30]. Research has demonstrated that in addition to antibiotics, HMs [31] and pesticides [32] can also promote the exchange of genetic elements responsible for antibiotic resistance. Unlike antibiotics, HMs are not easily degraded, and they can exert long-term selective pressure when accumulated in the environment [33]. Numerous studies have revealed positive correlations between ARG and HM levels in environments exposed to anthropogenic pressure [34,35]. High concentrations of HMs in the environment induce co-selection mechanisms which activate the expression of one or more genes encoding resistance to antibiotics and HMs under the influence of a single stressor [5]. The presence of HMs that induce selective pressure in the environment can increase resistance to antibiotics and HMs. Copper and zinc are widely used in industrial production, and they contribute to an increase in the concentrations of selected ARGs [36]. According to some researchers, HMs can induce resistance to antibiotics even in environments that are free of antimicrobials [37]. Selective pressure promotes the exchange of ARGs through horizontal gene transfer (HGT) between pathogenic and non-pathogenic microorganisms or even remotely related bacteria [38]. The dissemination of ARGs via HGT is most often linked with the presence of MGEs. Mobile genetic elements such as plasmids play an important role in the transfer of ARGs through conjugation [29], and according to many authors, integrons are also significantly implicated in the spread of antibiotic resistance [39]. Integrons contain a gene encoding the enzyme integrase which promotes the incorporation of genes responsible for resistance to antibiotics and HMs. These genes make up gene cassettes in the structure of integrons. Therefore, the transfer of integrons between microorganisms can lead to the acquisition of entire gene cassettes and, consequently, the spread of resistance in the environment [20].

As mentioned earlier, highly industrialized regions are a significant source of pollutants such as antibiotics and HMs. Industrial activity promotes the dissemination of antibiotic resistance which poses a considerable threat for public health and the ecological balance. Limited research papers determined the influence of industrialization factor to antibiotic resistance issue, comparing WWTPs placed in regions with different level of industrialization. Therefore, the aim of this study was to determine the correlations between the presence of pollutants such as antibiotics and HMs and the evolution of antibiotic resistance in two WWTPs situated in regions with different levels of industrial development. The factors exerting selective pressure, which significantly contributed to the occurrence of the examined antibiotic resistance genes (ARGs), were identified.

Materials and Methods

Research site

Samples for analysis were collected from two WWTPs located in regions with different levels of urban and industrial development and different population density. The first WWTP is situated in the Region of Warmia and Mazury (WM-WWTP), and the second WWTP – in the Region of Silesia (S-WWTP) in Poland (Europe). Silesia is the most industrialized Polish region where mining and metallurgy are the key areas of economic activity [40]. In turn, Warmia and Mazury is referred to as the "Green Lungs of Poland", and it is characterized by low levels of urbanization, high forest cover and the absence of heavy industry [41]. The percentage of the population living in urban areas is higher in Silesia (77.6%) than in Warmia and Mazury (59.3%). The compared regions also differ in population density which is estimated at 366 persons/ km^2 in Silesia and only 59 persons/ km^2 in Warmia and Mazury [42].

Both WWTPs deploy mechanical and biological wastewater treatment systems, but an additional technology for the chemical removal of phosphorus has been implemented in S-WWTP. WM-WWTP processes wastewater from the capital city of the Region of Warmia and Mazury and four surrounding municipalities, and it has an average daily processing capacity of 35,000 m^3. The plant has a population equivalent (PE) of 260,361, and treated wastewater is characterized by chemical oxygen demand (COD) of 49 mg/L, and biological oxygen demand (BOD) of 6 mg/L. Industrial sewage accounts for only 8% of the total wastewater processed by WM-WWTP [43,44]. The processing technology involves mechanical treatment systems with automated screens for separating large solids, two horizontal sand filters, and two sedimentation tanks. The biological treatment system is composed of phosphate and nitrogen removal chambers, and five multifunctional bioreactors equipped with four Passavant aerating rotors each and secondary sedimentation tanks. Sewage sludge is inactivated in closed and open

digestion chambers, and it is dehydrated by a mechanical filter press. Processed wastewater is discharged to a river [45].

Wastewater from local cities and factories is supplied to S-WWTP by four trunk lines. The plant receives 32,731 m^3 of wastewater each day and has a PE of 403,138. Industrial sewage accounts for up to 25% of the wastewater processed by S-WWTP. Wastewater entering the plant via trunk collectors is pumped to a pre-treatment line composed of hook screens, belt conveyors and a manual screen for the separation of large solids. Pretreated wastewater is then passed through horizontal sand filters. Filtered wastewater undergoes biological treatment in the C-TECH reactor. In the first stage, phosphorus is precipitated chemically with an iron coagulant. Wastewater is then directed to the reactor where it is treated in a repeated series of aeration, sedimentation, and decantation processes. The last stage of biological treatment involves phosphorus removal, denitrification and nitrification. The generated sewage sludge is neutralized in anaerobic digestion chambers, and it is dehydrated in a high-speed decanter centrifuge and a belt filter press [46]. Schematic diagrams of the analyzed WWTPs are presented in S1 and S2 Fig. in the supplementary materials.

Sample collection

Samples of wastewater, sewage sludge and river water were collected in June and November 2018. Wastewater and sewage sludge were sampled in different stages of treatment in both WWTPs. Samples of river water were collected upstream and downstream from the wastewater discharge point. The sampling sites are described in detail in Table 1 and S1 and S2 Fig. Samples of wastewater and river water were collected into sterile 500 ml bottles (SIMAX), and sewage sludge was sampled with the use of 100 ml plastic tubes. Wastewater was sampled in triplicate at hourly intervals for 24 h to obtain representative samples, and the collected hourly samples were pooled to a composite sample. Samples of river water and sewage sludge

were also collected in triplicate to create complex samples. The samples were transported to the laboratory at a temperature of 4°C.

Table 1. **Description of collected samples**

WM-WWTP		S-WWTP	
Symbol	Sample	Symbol	Sample
WM1	Raw wastewater	S1	Raw wastewater
WM2	Wastewater after primary clarifier	S2	Wastewater after primary clarifier
WM3	Wastewater treated in biological chamber	S3	Wastewater after secondary clarifier
WM4	Wastewater treated in multifunctional reactor	S4	Wastewater treated in C-TECH reactor
WM5	Treated wastewater	S5	Treated wastewater
WM6	River water – upstream form the effluent discharge point	S6	River water – upstream form the effluent discharge point
WM7	River water – downstream form the effluent discharge point	S7	River water – downstream form the effluent discharge point
WM8	Sewage after the open fermentation tank	S8	Sewage sludge dewatering leachates
WM9	Dewatered sewage sludge	S9	Mechanically compacted sewage sludge
		S10	Sewage sludge after gravity thickening

Determination of antibiotics

Chemicals

Ciprofloxacin (CIP), norfloxacin (NOR), ofloxacin (OFX), oxytetracycline (OTC), pefloxacin (PEF), sulfadimethoxine (SDM), sulfamethoxazole (SXT), sulfathiazole (ST) and tetracycline (TET) of high purity grade (>90%) were purchased from Sigma–Aldrich (Germany). Ciprofloxacin-D8 (CIP-D8), sulfamethoxazole-D4 (SXT-D4), ofloxacin-D8 (OFX-D8) and tetracycline-D6 (TET-D6) were obtained from Toronto Research Chemicals

(Toronto, Canada). The solvents, HPLC gradient grade methanol, acetonitrile (LiChrosolv) and formic acid (98%) were supplied by Merck (Germany). Ultrapure water was obtained from a Millipore water purification system (Milli-Q water).

Sample preparation

Samples were prepared according to a previously described procedure [21]. Briefly, wastewater samples were filtered through glass fiber (GF/C, Whatman) and membrane filters (0.2 µm, Sartorius). Samples of pretreated wastewater in the amount of 100 mL (diluted with water, v/v, 1:1, P1 or P8) or 200 mL, containing 200 mg of ethylenediaminetetraacetic acid and a mixture of internal standards (0.5 mL, 1 µg/mL of CIP-D8, SXT-D4, OFX-D8 and TET-D6 in methanol) were loaded onto Oasis HLB solid phase extraction columns (3 mL, 400 mg; Waters Corp., Milford, MA). The elution was performed with methanol (3×2 mL). The eluents were evaporated under a stream of nitrogen at 40°C, reconstituted in a methanol–water mixture (10:90 v/v) (1 mL) and injected onto an HPLC column (10 µL).

Sewage sludge samples were centrifuged at 5000 g for 5 min, and the supernatant were discarded. Specimens of 5 g (WM9) or 10 g were placed in 50 mL polypropylene tubes, combined with 50 µL of the internal standard mixture, 9 mL of 30 mM KH_2PO_4 and 1 ml of methanol, and extracted for 20 min using 10 mL of acetonitrile with 1% formic acid and modified QUECHERS salts (4 g of $MgSO_4$, 1 g of NaCl, 1 g of Na_3Citrate and 0.5 g of Na_2Citrate·H2O). Next, the organic layer was washed with an octadecyl sorbent and $MgSO_4$ and centrifuged at 5000 g for 5 min. The extract was evaporated under a nitrogen stream at 40°C, reconstituted in 0.5 mL of methanol-water mixture (10:90) and injected onto an HPLC column (10 µL).

Analysis of heavy metal concentrations

The content of Zn, Pb, Ni, Cr, Co and As was determined in samples of wastewater, sewage sludge and river water. The analyses were performed by an accredited laboratory of the Institute for the Ecology of Industrial Areas in Katowice, Poland. The concentration of As in environmental samples was determined by hydride generation atomic absorption spectroscopy (HGAAS) (accreditation No. PB-05/4), and the content of the remaining HMs was determined by inductively coupled plasma atomic emission spectroscopy (ICP-OES) (PN-EN ISO 11885:2009) [47].

Isolation of environmental DNA

Samples of wastewater and river water were passed through polycarbonate membrane filters (porosity – 0.2 µm, diameter – 47 mm) with the use of a vacuum pump (Millipore, Merck). The filters were placed in sterile 10 mL Falcon tubes, and 5 mL of 1X PBS solution was added. Filtrate samples were shaken for 5 h at room temperature. The resulting suspension was transferred to sterile 2 mL Eppendorf tubes and centrifuged for 5 minutes at 8,000 rpm at a temperature of 4°C. The supernatant was discarded, and the pellet obtained from wastewater and river water samples was used for the isolation of genomic DNA with the DNeasy Power Water Kit (Qiagen). Genomic DNA was directly isolated from sewage sludge samples of 0.5 g each with the DNeasy Power Soil Kit (Qiagen). DNA was isolated in accordance with the manufacturer's instructions. The purity of the obtained genetic material was determined with a spectrophotometer (MultiSky, ThermoScientific).

Real-Time quantitative PCR (qPCR)

The copy numbers of genes encoding resistance to beta-lactams (*bla*$_{TEM}$, *bla*$_{OXA}$, *bla*$_{SHV}$), tetracyclines (*tet*(A), *tet*(M)), fluoroquinolones (*qep*A, *aac*(6`)-*Ib-cr*), sulfonamides (*sul*1, *sul*2), integrase genes (*int*I1, *int*I2), and the 16S rRNA gene were determined by quantitative PCR with the SYBR Green fluorescent dye. Primer sequences, qPCR conditions and amplification efficiency are presented in S1 Table. All qPCR assays were conducted in the Roche LightCycler® 480 thermocycler (Roche Applied Science, Indianapolis, IN, USA). The reaction mix with a total volume of 15 µL was composed of 0.75 µL of the DNA matrix, 0.375 µL of each primer (10 µM), 3.75 µL of the SYBR GREEN master mix (Roche), and 9.75 µL of sterile water (HighPure H_2O, Roche). All samples were analyzed in triplicate. Every qPCR assay had a positive control (linearized plasmid with a specific gene insert) and a negative control (HighPure H_2O, Roche). Standard curves were generated by cloning the amplicon from the control to the pCR2.1-TOPO vector (Invitrogen, USA). Standard curves for qPCR were obtained in a range of 10^8 to 10^2 of gene copies/µl [48]. Standard curves were generated according to Applied Biosystems guidelines ("Creating Standard Curves with Genomic DNA or Plasmid DNA Templates for Use in Quantitative PCR").

Data analysis

Data were processed statistically in the Statistica 13.3 program (StatSoft Inc.) with the use of the following non-parametric tests: Kruskal-Wallis test, Mann-Whitney U test, and Wilcoxon signed-rank test. The correlations between gene copy numbers in the analyzed samples were determined by the mean-ranks post-hoc test for multiple comparisons. The correlations between the concentrations of the examined genes and the content of HMs and antibiotics were determined by Spearman's rank-order correlation test [49]. Statistical

significance was set at $p<0.05$. A correlation matrix was developed in RStudio v. 1.2.5033 using the "corrplot" package. A heatmap was generated and Ward's cluster analysis of gene copy numbers in the evaluated sites was performed in RStudio using "heatmap.2" and "gplots" packages. A network analysis of the correlations between the analyzed variables was conducted in Gephi v. 0.9.2 [50]. A statistical analysis of antibiotic data was performed in MetaboAnalyst 4.0 [51]. The variables were log transformed to meet ANOVA assumptions. The ANOVA was followed by Tukey's post-hoc test. To better visualize antibiotic concentrations, data were normalized by dividing the results by the mean concentrations of all variables and cases (separately for S-WWTP and WM-WWTP).

Results and Discussion

Antibiotic concentrations

The concentrations of antibiotics in influents and effluents have been widely analyzed in the literature, and antibiotic levels in different stages of wastewater treatment, including in sewage sludge, have been compared in recent studies [17–19,52]. In the presented study, antibiotic concentrations were determined in eight (S-WWTP) and seven sites (WM-WWTP) in the analyzed WWTPs, including in sludge collectors (WM8, WM9, S9, S10). Samples of river water collected downstream and upstream from the wastewater discharge point were also analyzed. Antibiotic average concentrations in the analyzed samples are presented in Fig. 1 and 2, and in S2 Table and S3-S11 Fig. in the supplementary materials. Antibiotic levels were determined in the range of <LOD - 1.2x10^{3} ng/g. The samples from both WWTPs had the highest concentrations of fluoroquinolones, including CIP (0.0128-1179.9 ng/g), NOR (<LOD-319.7 ng/g), OFX (0.0047-66.1 ng/g) and PEF (<LOD-29 ng/g), and the lowest concentrations of sulfonamides, including SDM (<LOD-1.59 ng/g), SXT (<LOD-2.98 ng/g) and ST (<LOD-1.67 ng/g). Similar dependencies between antibiotic concentrations were reported by Dong et

al. [53] in wastewater samples. The following abundance pattern was proposed based on antibiotic concentrations in the samples from both WWTPs: CIP>NOR>OFX>PEF>OTC>TET>SXT>ST>SDM (S2 Table).

Fig. 1. Heatmap of normalized concentrations of antibiotics in different sampling points of WM-WWTP. Red colour indicates high concentration, whereas blue colour low concentration.

In general, the concentrations of most antibiotics were significantly higher in sludge than in wastewater discharged by both WWTP and in river water (S2 Table, Fig. 1 and 2). The mean antibiotic concentrations were 3-4 orders of magnitude higher for NOR and OTC, and 2-3 orders of magnitude higher for CIP, OFX, SDM, TET and PEF. No significant differences in SXT levels were noted between sludge and the aqueous phase. Sulfathiazole was not detected in sludge, but conclusive results could not be obtained because the limit of detection was significantly higher in the analysis of sludge than the aqueous phase. The obtained results confirmed that fluoroquinolones and tetracyclines are eliminated mainly by sorption to sludge, whereas SXT are less effectively removed by this mechanism [54,55].

Fig. 2. Heatmap of normalized concentrations of antibiotics in different sampling points of S-WWTP. Red colour indicates high concentration, whereas blue colour low concentration.

Wastewater treatment significantly decreased the concentrations of CIP, OFX, NOR and ST in the aqueous phase (significant differences were observed between S1 and S5 and between WM1 and WM5) (S2 Table, S3-S5 Fig., S10 Fig.). On average, CIP concentrations were reduced 14-fold (18-fold in WM-WWTP; 10-fold in S-WWTP) (S2 Table, S3 Fig.), OFX concentrations were reduced 7-fold (6-fold in WM-WWTP; 8-fold in S-WWTP), NOR concentrations were reduced 8-fold (in S-WWTP; NOR was detected only in sludge in WM-

WWTP) (S2 Table, S4 Fig.), and ST concentrations were reduced 5-fold (in both plants) (S2 Table, S10 Fig.). A significant decrease in TET concentration (100 fold) was observed only in S-WWTP (S2 Table, S11 Fig.). No changes or a minor decrease in the concentrations of SDM, OTC (7-fold decrease in S-WWTP – not significant; the results noted in WM-WWTP were below the LOD) and SXT (3-fold, significant only in WM-WWTP) were noted (S2 Table, S6 Fig., S8 Fig., S9 Fig.).

In the Mann-Whitney U test, OTC and NOR were the only antibiotics whose concentrations differed significantly ($p<0.05$) between the examined WWTPs, where S-WWTP located in industrial region was characterized with higher abundance of those pollutants (S3 Table). Literature pointed out the concentration increase of oxytetracycline and norfloxacin in wastewater and even river sediments due to industrial activities [56–58].

Treated wastewater is discharged to rivers. Significantly higher concentrations of SXT, TET (WM-WWTP) and ST (S-WWTP) were observed in river water sampled downstream (WM7/S7) than upstream (WM6/S6) from wastewater discharge point (S2 Table, S9-S11 Fig.). The concentrations of the remaining antibiotics were low in river water. Wastewater treatment plants have been identified as hot spots of aquatic contamination with pharmaceuticals around the world [21,59,60].

Heavy metal concentrations

In this subsection, the terms of liquid samples (river water, wastewater and sewage sludge (WM8) samples) and solid samples (sewage sludge (WM9/S9/S10) samples) were used because of the method used to extract heavy metals from researched samples and the differences of concentration units. Heavy metal concentrations were determined in a range of <LOD - 551 mg/L in liquid samples, and <LOD - 1090.8 mg/kg DM (dry matter) in solid samples collected from both WWTPs. Heavy metal average concentrations in all samples are presented in Tables

2 and 3. Liquid and solid samples from both WWTPs were most abundant in zinc in a concentration range of <LOD-551 mg/L and 706.4-1090.8 mg/kg DM, respectively (Tables 2 and 3). Arsenic was the least abundant HM in both liquid (<LOD-1.27 mg/L DM) and solid (1.15-2.75 mg/kg DM) samples (Tables 2 and 3). Tella et al. [61] found similar dependencies in samples of sewage sludge, whereas in the work of Olujimi et al. (2012), HM concentrations were one order of magnitude higher than in the present study.

In current research, we observed slightly higher HMs concentration in S-WWTP influent wastewater (S1:WM1) comparing to WM-WWTP (Zn 0.254:0.248; Pb 0.0183:<LOD; Ni 0.014:<LOD; Cr 0.0115:0.01 mg/L, respectively). Due to lack of similar comparisons in research papers of HMs concentration in WWTPs during treatment process in industrial and non-industrial regions, we found this issue hard to discuss.

The concentrations of Pb in sewage sludge differed between the analyzed WWTPs. The samples from WM-WWTP were less abundant in Pb, but the observed difference was not statistically significant (15.72-16.7 mg/kg d.m. in WM-WWTP and 19-40.05 mg/kg d.m. in S-WWTP) (Tables 2 and 3, S7 Table). The above could be attributed to the fact that S-WWTP is situated in an industrial region and processes more industrial wastewater. Other authors also reported higher HMs levels in industrial wastewater and mixed municipal and industrial wastewater [6,61].

An analysis of HM concentrations in the samples collected from both WWTPs (Tables 2 and 3) revealed that treatment process affects decreasingly on HMs concentration, but without statistical significance (M-W $p>0.05$) in site WM1/S1 relative to site WM5/S5. Literature presented reduction of HMs concentration in industrial and non-industrial polluted wastewater, due to treatment process [63,64]. The evacuation of treated wastewater did not increase HM concentrations in river water in site WM6/S6 relative to site WM7/S7 (M-W $p>0.05$).The sewage sludge was characterized by the highest HMs concentrations which differed

significantly (M-W $p>0.05$) from those noted in wastewater samples (S5 and S6 Table). The Mann-Whitney U test did not reveal significant differences ($p>0.05$) in the HM content of samples from both WWTPs (S7 Table). The examined WWTPs are not equipped with specialized systems for removing HMs. Heavy metals are strongly adsorbed on solid particles, and they accumulate in the sludge. According to the literature, HMs are eliminated from untreated wastewater already in settling tanks. Heavy metal removal can be regarded as a by-product of the wastewater treatment process [8,62,65].

Table 2. Average concentration of analysed heavy metals (mg/L and mg/kg d.m.) in samples from WM-WWTP

WM-WWTP															
June								November							
Sampling sites		Zn	Pb	Ni	Cr	Co	As	Sampling sites		Zn	Pb	Ni	Cr	Co	As
WM1	mg/L	0.247	<0.015	<0.009	0.013	<0.006	0.004	WM1	mg/L	0.249	<0.015	<0.009	0.008	<0.006	0.002
WM2		0.155	<0.015	<0.009	0.010	<0.006	0.003	WM2		0.138	0.022	<0.009	<0.005	<0.006	0.001
WM3		3.018	0.047	0.039	0.090	<0.006	0.018	WM3		2.420	0.032	0.068	0.104	<0.006	0.005
WM4		3.034	0.045	0.037	0.084	<0.006	0.015	WM4		2.640	0.037	0.068	0.112	<0.006	0.005
WM5		0.065	<0.015	<0.009	0.005	<0.006	0.002	WM5		0.050	<0.015	<0.009	<0.005	<0.006	<0.0005
WM6		0.011	<0.015	<0.009	0.008	0.056	0.008	WM6		<0.005	<0.015	<0.009	<0.005	<0.006	<0.0005
WM7		0.010	<0.015	<0.009	0.008	<0.006	0.011	WM7		<0.005	<0.015	<0.009	<0.005	<0.006	<0.0005
WM8		6.582	0.252	0.158	0.493	0.053	0.057	WM8		551	7.6	38.3	31	13.5	1.27
WM9	mg/kg d.m.	1090.8	16.7	25.8	53.3	<2	2.745	WM9	mg/kg d.m.	934.73	15.72	8.38	51.58	8.27	2.69

Table 3. Average concentration of analysed heavy metals (mg/L and mg/kg d.m.) in samples from S-WWTP

S-WWTP															
June								November							
Sampling sites		Zn	Pb	Ni	Cr	Co	As	Sampling sites		Zn	Pb	Ni	Cr	Co	As
S1	mg/L	0.299	0.021	0.017	0.017	<0.006	0.004	S1	mg/L	0.209	0.0156	0.011	0.006	<0.006	0.001
S2		0.132	<0.015	0.014	0.009	<0.006	0.002	S2		0.077	<0.015	0.012	0.010	<0.006	0.004
S3		0.027	<0.015	0.015	0.007	<0.006	<0.0005	S3		0.017	<0.015	<0.009	<0.005	<0.006	<0.0005
S4		0.049	<0.015	0.012	0.006	<0.006	<0.0005	S4		0.051	<0.015	0.006	<0.005	<0.006	<0.0005
S5		0.031	<0.015	0.014	0.006	<0.006	0.009	S5		0.018	<0.015	0.006	<0.005	<0.006	<0.0005
S6		0.034	<0.015	0.012	0.008	0.021	<0.0005	S6		0.011	<0.015	0.003	<0.005	<0.006	<0.0005
S7		0.022	<0.015	<0.009	0.006	<0.006	<0.0005	S7		0.014	<0.015	0.006	<0.005	<0.006	<0.0005
S8		3.077	0.088	0.132	0.119	0.064	0.026	S8		0.519	<0.015	0.041	0.075	0.0160	0.003
S9	mg/kg d.m.	772.3	23.37	33.74	39.4	4.6	1.707	S9	mg/kg d.m.	706.4	19.0	25.08	72.96	4.94	1.15
S10		841.8	40.05	38.17	61.0	2.1	2.429	S10		767.8	24.96	24.18	45.09	0.83	1.70

Quantification of antibiotic resistance genes

Quantitative PCR supported the identification of nine ARGs, two integrase genes and the 16S rRNA gene in the analyzed samples. The concentrations of the identified genes, excluding the 16S rRNA gene, ranged from 10^{1} - 10^{9} gene copies/mL in wastewater to 10^{3} - 10^{11} gene copies/g in sewage sludge. The average concentrations of the analyzed genes are presented in Fig. 3 and 4 and in S8 and S9 Table in the supplementary materials.

Genes conferring resistance to sulfonamides were most abundant in WM-WWTP. The content of *sul*1 and *sul*2 genes ranged from <LOD-$2.77x10^{9}$ gene copies/mL of wastewater (*sul* genes were detected in 86% of wastewater samples) to $1.22x10^{10}$-$5.23x10^{11}$ gene copies/g of sewage sludge. In turn, *qep*A and *aac*(6')-*Ib-cr* genes encoding resistance to fluoroquinolones were least abundant at <LOD-$7.7x10^{7}$ gene copies/mL and <LOD-$8.66x10^{7}$ gene copies/g (*qep*A and *aac*(6')-*Ib-cr* were identified in 86% of wastewater samples and in 63% of sludge samples) (S8 Table). In the group of fluoroquinolone-resistance genes, *qep*A was less abundant than *aac*(6')-*Ib-cr* (S8 Table). The distribution of the analyzed ARGs followed highly similar patterns in S-WWTP and WM-WWTP. Samples from S-WWTP were characterized by the highest concentrations of *sul*1 and *sul*2 genes in a range of <LOD-$1.83x10^{9}$ gene copies/mL (*sul* genes were detected in 81% of wastewater samples) and $3.05x10^{10}$-$5.02x10^{11}$ gene copies/g. The least prevalent genes were *qep*A and *aac*(6')-*Ib-cr* which were determined in a concentration range of <LOD-$3.58x10^{8}$ gene copies/mL (*qep*A and *aac*(6')-*Ib-cr* were identified in 66% of wastewater samples) and $2.7x10^{3}$-$1.65x10^{10}$ gene copies/g (S9 Table).

Fig. 3. Distribution of log gene copies in WM-WWTP. Black spectrum colours indicate the highest average concentration, while white spectrum colours indicate the lowest concentration of genes copies. Dendrograms point out similarity clusters.

Chen et al. (2015) examined the prevalence of 22 ARGs in wastewater and surface water samples and found that sulfonamide resistance genes were the most abundant and the only group of genes that have been identified in all samples. Le et al. (2016) reported a higher concentration of the *sul*1 gene (10^8 copies/mL) than the *sul*2 gene (10^7 copies/mL) in wastewater samples, which corroborates the results of the present study. The high abundance of *sul*1 and *sul*2 genes could be attributed to their location in MGEs such as class 1 and 2 integrons [68]. Genes conferring resistance to fluoroquinolones were less abundant, which could be explained by the fact that these antibiotics are less frequently used in hospitals and the community [69]. The Mann-Whitney U test did not reveal significant differences ($p>0.05$) in gene concentrations between the examined WWTPs (S14 Table).

Fig. 4. Distribution of log gene copies in S-WWTP. Black spectrum colours indicate the highest average concentration, while white spectrum colours indicate the lowest concentration of genes copies. Dendrograms point out similarity clusters.

In addition to the high content of sulfonamide resistance genes, the samples collected from both WWTPs were also highly abundant in *int*I1 and *int*I2 genes whose concentrations were determined at $7.4x10^3$-$1.7x10^8$ gene copies/mL of wastewater and $1.24x10^7$-$3.81x10^{10}$ gene copies/g of sewage sludge (S8 and S9 Table). The *int*I1 gene is frequently detected in hospital wastewater [70], whereas *int*I2 is characteristic of bacteria isolated from human and animal feces that are a potential source of this gene in wastewater [71,72]. According to the literature, *sul* and *int*I are closely linked, and the *sul*1 gene is frequently identified in class 1 integrons [73,74]. Du et al. [75] pointed out that *sul*1 and *int*I1 were most abundant genes in wastewater samples contains 56% of industrial wastewater.

In the group of tetracycline resistance genes, *tet*(A) was more abundant that *tet*(M) in the samples collected from WM-WWTP, whereas *tet*(M) was not identified in the samples from

S-WWTP (S8 and S9 Table). A similar relationship was described by Chen and Zhang (2013) in whose study, the concentration of *tet*(M) in municipal wastewater was three orders of magnitude lower than the content of *tet*(A). Zhang and Zhang (2011) examined the prevalence of 14 genes encoding resistance to tetracyclines in samples collected from 15 WWTPs in China, and they found that *tet*(A) was the most abundant *tet* gene.

Wastewater (WM1/S1) is treated during a series of technological processes, and treated wastewater is evacuated to rivers (WM5/S5). An analysis of the samples collected from both WWTPs revealed that the concentrations of the examined genes decreased by 2 to 3 orders of magnitude between sites WM1/S1 and WM5/S5 (S8 and S9 Table). According to many authors, the concentrations of resistance genes are reduced by 3 orders of magnitude on average in WWTPs that deploy the activated sludge process [78,79]. The Kruskal-Wallis non-parametric test demonstrated a significant reduction in gene levels between samples of untreated and treated wastewater (K-W $p=0.00$) (S12 and S13 Table). The percentage reduction of ARGs, integrase genes and the 16S rRNA gene ranged from 95.5% to 99.6% in both WWTPs (S8 and S9 Table).

The evaluated WWTPs evacuate treated wastewater to rivers. Effective WWTPs should not contribute to the contamination of rivers that act as receptacles of treated wastewater. An analysis of the concentrations of ARGs, integrase genes and the 16S rRNA gene in river water sampled upstream (WM6/S6) and downstream (WM7/S7) from the wastewater discharge point revealed that the content of the studied genes increased by one order of magnitude after treatment (S8 and S9 Table). Despite the higher abundance of genes in river water sampled downstream from the wastewater discharge point, the observed differences were not significant (K-W $p=1.00$) (S12 and S13 Table). An increase in pollution levels in rivers receiving treated wastewater was also frequently reported in the literature [60,74]. The steady release of ARGs

from WWTPs to rivers constitutes an important external source of antibiotic resistance in the natural environment [10,80].

The analyzed genes were most abundant in sewage sludge samples collected from both WWTPs, and their mean concentrations were determined at 4.56×10^{11}-1.92×10^{12} gene copies/g in WM-WWTP and 9.65×10^{11}-7.59×10^{12} gene copies/g in S-WWTP (S8 and S9 Table). The copy numbers of ARGs and integrase genes were 2 to 3 orders of magnitude higher in sewage sludge than in all wastewater samples (S8 and S9 Table). Equally high concentrations of ARGs and integrase genes were noted in sewage sludge by Birošová et al. (2014) and Luo et al. (2010). A statistical analysis of gene copy numbers in wastewater samples (regardless of sampling site) and sludge samples revealed significant differences, excluding the samples from sites WM1, WM2 and S1 (K-W $p>0.25$) (S12 and S13 Table). The volume of sewage sludge generated in municipal WWTPs in Poland increased by 38% between 2000 and 2018. In 2018, municipal WWTPs produced 583,000 tons of sludge dry solids, 20% of which was used in agriculture and 19.1% was thermally processed [83]. This study confirmed that sewage sludge is heavily contaminated with genetic determinants of resistance. Sewage sludge is abundant in organic matter and biogenic elements, and it is widely used as agricultural fertilizer. Research has demonstrated that long-term soil fertilization with sewage sludge increases ARG levels and promotes the spread of antibiotic resistance in soil [84].

A comparison of the relative concentrations of the studied genes and the 16S rRNA gene confirmed that *sul*1, *sul*2 and *int*I1 were most ubiquitous, whereas *qep*(A) was the least abundant gene (Fig. 5 and 6). On average, gene concentrations increased by one order of magnitude between sampling sites WM1/S1 and WM5/S5 (S10 and S11 Table). Osińska et al. (2019) also reported an increase of one order of magnitude between relative gene concentrations in untreated and treated wastewater. The results of the present study suggest that ARGs and integrase genes can be more abundant in treated wastewater than in untreated wastewater even

in WWTPs equipped with effective treatment systems. These observations can be attributed to HGT which promotes the spread of ARGs to bacteria during wastewater treatment. Proia et al. (2016) also observed an increase in the copy numbers of genetic elements that confer antibiotic resistance downstream from the wastewater discharge point in WWTPs with effective treatment process. Antibiotic resistance genes are evacuated with incompletely treated municipal and hospital wastewater, and they pose an environmental threat and contribute to the emergence of multidrug-resistant bacteria [87]. Despite the implementation of new wastewater processing technologies, there is a general scarcity of methods that effectively remove ARGs from wastewater, which poses a significant risk for humans and ecosystems [26]. In the current study, the average concentrations of the analyzed genes increased in samples of river water collected downstream from the wastewater discharge point in both WWTPs, but the observed increase was not statistically significant (Figures 5 and 6) (S12 and S13 Table).

Fig. 5. Relative concentration (log gene copies/16S rRNA) of researched genes in WM-WWTP, both seasons.

Fig. 6. Relative concentration (log gene copies/16S rRNA) of researched genes in S-WWTP, both seasons.

Impact of industrialization on antibiotic resistance – correlation analysis

High concentrations of antibiotics and HMs in municipal and industrial wastewater induce the spread of antibiotic resistance around the world, which is why the correlations between these variables should be analyzed [10,29].

The correlations between the studied genes, antibiotics and HMs in the samples collected from both WWTPs are presented in Fig. 7 and 8 and S12 and S13 Fig. The results of the statistical analysis confirm the presence of strong correlations between the copy numbers of integrase genes and sulfonamide resistance genes (*sul*1 and *sul*2) in samples from both WWTPs. Di Cesare et al. (2016b) also reported strong correlations between integrase genes in wastewater. However, in the present experiment, no significant correlations were noted between sulfonamide concentrations and the abundance of sulfonamide resistance genes in any of the WWTPs. An absence of significant correlations between *sul*1 and *sul*2 genes and sulfonamide levels was also reported by Xu et al. (2015). Giebułtowicz et al. (2020) analyzed the relationship between antibiotic concentrations in wastewater and antibiotic resistance found that sulfonamides were low or average risk factors for the spread [illegible]tibiotic resistance. In the present study, no significant correlations were observed between sulfonamide levels and the copy numbers of integrase genes (*int*I1 and *int*I2). In the samples collected from both WWTPs, the abundance of *tet*(A) and *tet*(M) genes was not significantly correlated with tetracycline concentrations. An absence of significant correlations between the copy numbers of *tet* genes vs. TC and OTC concentrations was reported in samples of wastewater and river water in China [10,88] (S12 and S13 Fig.).

The abundance of *qep*A and *aac*(6')-*Ib-cr* genes in the studied samples was correlated with fluoroquinolone concentrations. In the samples from WM-WWTP, a significant correlation was noted between *qep*A copy numbers and PEF levels, whereas in the samples

from S-WWTP, the concentrations of PEF, CIP and PEF were significantly correlated with the abundance of *qep*A and *aac*(6')-*Ib-cr* genes, respectively (S12 Fig.). Similar results were reported for fluoroquinolones by Giebułtowicz et al. (2020) who found that ciprofloxacin was a high risk factor for the dissemination of antibiotic resistance.

Fig. 7. Network analysis of correlated variables in WM-WWTP. All correlations in diagram are statistically significant. Edges: green - positive correlations, red - negative correlations; Nodes: green – heavy metals, purple – genes, orange – antibiotics.

According to the literature [89], the prevalence of ARGs in the environment should be closely correlated with the concentrations of antibiotics that exert selective pressure on microorganisms. However, Lu et al. (2015) found that antibiotics did not significantly contribute to the dissemination of ARGs. In the present study, no significant correlations were observed between sulfonamide and tetracycline concentrations and the most prevalent genes encoding resistance to these antibiotics. The above could be attributed to the fact that tetracyclines and sulfonamides were detected at low concentrations in the analyzed samples. Despite low tetracycline and sulfonamide concentrations, the abundance of the associated ARGs was high, which could point to the presence of other selective factors in the environment, including HMs. Selective pressure could be the most problematic factor in the process of assessing the evolution of resistance. Numerous environmental factors drive variation among microorganisms, including antibiotics whose concentrations significantly change over time due to degradation. As a result, the magnitude of selective pressure is difficult to determine outside the laboratory [90]. Antibiotics are degraded much more rapidly than HMs which are retained in wastewater and promote the spread of antibiotic resistance. This observation leads to doubt

as to whether high concentrations of ARGs and integrase genes should be correlated with the concentrations of antibiotics [5] or rather HMs.

Fig. 8. Network analysis of correlated variables in S-WWTP. All correlations in diagram are statistically significant. Edges: green - positive correlations, red - negative correlations; Nodes: green – heavy metals, purple – genes, orange – antibiotics.

Correlations between gene abundance and HM levels were more frequently noted in the samples collected from S-WWTP than WM-WWTP (Figs. 7 and 8). The above could be attributed to higher mean HM concentrations in the samples from S-WWTP which is situated in a highly industrialized region. In both WWTPs, Zn, Pb, Ni and Cr were the most abundant HMs, which increased the frequency of the correlations between HMs and the analyzed genes. Chihomvu et al. [91] imply that gene encoding antibiotic resistance are located in the same position of chromosome or mobile genetic elements with heavy metal resistance genes, which highlights the position of HMs as co-selective factor for antibiotic resistance dissemination. Wastewater with industrial effluents can contain greater concentrations of emergence contaminants (as heavy metals or pharmaceuticals) by up to several orders of magnitude in comparison to municipal wastewater [58,92,93]. Strong correlations were noted between Cr, Ni and Zn levels and the copy numbers of *sul*1, *sul*2, *int*I1 and *int*I2 genes ($r > 0.7$ in all cases, $p < 0.05$) (S12 and S13 Fig.). Equally high correlations between ARGs and HMs were reported by Zhang et al. (2016) in a study of samples from a municipal WWTP. According to the literature, the prevalence of *sul* genes is significantly correlated with HM contamination [95,96].

The presence of correlations between the analyzed genes and antibiotics was more frequently noted in the samples collected from S-WWTP than WM-WWTP. These results could be attributed to higher concentrations of HMs and a higher number of significant correlations

between the examined genes and HMs in S-WWTP. Further research is needed to confirm the above observations. Xu et al. [97] indicate strong significant correlation between antibiotic resistance genes and heavy metals in wastewater contains industrial sewage.

In the current study, the network analysis revealed high correlations between the examined genes and HMs in the samples from WWTP located in industrial region, whereas far fewer correlations were noted between gene abundance and antibiotic concentrations (Figs. 7 and 8). Regions characterized with higher level of industrialization, release to environment high concentration of pollutants (HMs in particular), what enhance the antibiotic resistance issue through selective pressure phenomenon [32,98–102]. Heavy metals exert a permanent influence on the spread of ARGs and integrase genes in the environment, which can pose a serious threat for ecosystems and public health [94]. The present findings indicate that antibiotics are not always the only factor responsible for the presence of ARGs in the environment. Sulfonamides have a long half-life, and their widespread use in human and veterinary medicine affects the concentration and accumulation of these antibiotics in the environment. The continuous presence of subclinical concentrations of sulfonamides in the environment can decrease the significance of these drugs as selective factors that induce antibiotic resistance [103]. As a result, antibiotic resistance can be promoted mainly by HMs. Literature presented evidence that, plasmid indicated antibiotic resistance can be induced by the selective pressure of heavy metals in the environment. Thus, beside overuse of antibiotics, environmental heavy metal pollution can lead to serious problems [102].

Conclusions

This study investigated the correlations between the prevalence of ARGs, integrase genes and the factors that exert selective pressure and promote the spread of antibiotic resistance in wastewater, such as HMs and antibiotics. The present findings indicate that even WWTPs with effective treatment systems act as hotspots of antibiotic resistance in the environment. Sewage sludge was characterized by the highest concentrations of the analyzed chemical compounds and biological pollutants. The presence of correlations between integrase genes (*int*I1 and *int*I2), ARGs and HMs indicates that integrons (as MGEs) play a very important role in the spread of antibiotic resistance. Samples from S-WWTP which is located in a highly industrialized region were characterized by higher number of significant correlations between the investigated micropollutants and a higher concentrations of HMs. The results of the correlation analysis indicate that HMs induce antibiotic resistance in the environment. Correlation networks from both regions, shown a straight forward display that industrialization factor is a driving force for dissemination and maybe even diversification of antibiotic resistance genes in environment.

References

1. Zouiten A, Beltifa A, Van Loco J, Mansour H Ben, Reyns T. Ecotoxicological potential of antibiotic pollution–industrial wastewater: bioavailability, biomarkers, and occurrence in Mytilus galloprovincialis. Environ Sci Pollut Res. 2016;23: 15343–15350. doi:10.1007/s11356-016-6713-2

2. Vignaroli C, Pasquaroli S, Citterio B, Di Cesare A, Mangiaterra G, Fattorini D, et al. Antibiotic and heavy metal resistance in enterococci from coastal marine sediment. Environ Pollut. 2018;237: 406–413. doi:10.1016/j.envpol.2018.02.073

3. Kumar M, Jaiswal S, Sodhi KK, Shree P, Singh DK, Agrawal PK, et al. Antibiotics bioremediation: Perspectives on its ecotoxicity and resistance. Environ Int. 2019;124: 448–461. doi:10.1016/j.envint.2018.12.065

4. Sanchez LF, Stern DI. Drivers of industrial and non-industrial greenhouse gas emissions. Ecol Econ. 2016;124: 17–24. doi:10.1016/j.ecolecon.2016.01.008

5. Di Cesare A, Eckert EM, Corno G. Co-selection of antibiotic and heavy metal resistance in freshwater bacteria. J Limnol. 2016;75: 59–66. doi:10.4081/jlimnol.2016.1198

6. Cheng M, Wu L, Huang Y, Luo Y, Christie P. Total concentrations of heavy metals and occurrence of antibiotics in sewage sludges from cities throughout China. J Soils Sediments. 2014;14: 1123–1135. doi:10.1007/s11368-014-0850-3

7. Rorat A, Courtois P, Vandenbulcke F, Lemiere S. Sanitary and environmental aspects of sewage sludge management. Industrial and Municipal Sludge. Elsevier; 2019. pp. 155–180. doi:10.1016/B978-0-12-815907-1.00008-8

8. Fijalkowski K, Rorat A, Grobelak A, Kacprzak MJ. The presence of contaminations in

sewage sludge – The current situation. J Environ Manage. 2017;203: 1126–1136. doi:10.1016/j.jenvman.2017.05.068

9. Europejskich DUW. Dyrektywa Rady w sprawie ochrony środowiska, w szczególności gleby, w przypadku wykorzystywania osadów ściekowych w rolnictwie. 1986;265.

10. Xu J, Xu Y, Wang H, Guo C, Qiu H, He Y, et al. Occurrence of antibiotics and antibiotic resistance genes in a sewage treatment plant and its effluent-receiving river. Chemosphere. 2015;119: 1379–1385. doi:10.1016/j.chemosphere.2014.02.040

11. Yi X, Lin C, Ong EJL, Wang M, Zhou Z. Occurrence and distribution of trace levels of antibiotics in surface waters and soils driven by non-point source pollution and anthropogenic pressure. Chemosphere. 2019;216: 213–223. doi:10.1016/j.chemosphere.2018.10.087

12. Burke V, Richter D, Greskowiak J, Mehrtens A, Schulz L, Massmann G. Occurrence of Antibiotics in Surface and Groundwater of a Drinking Water Catchment Area in Germany. Water Environ Res. 2016;88: 652–659. doi:10.2175/106143016x14609975746604

13. Castiglioni S, Davoli E, Riva F, Palmiotto M, Camporini P, Manenti A, et al. Mass balance of emerging contaminants in the water cycle of a highly urbanized and industrialized area of Italy. Water Res. 2018;131: 287–298. doi:10.1016/j.watres.2017.12.047

14. Deblonde T, Cossu-Leguille C, Hartemann P. Emerging pollutants in wastewater: A review of the literature. Int J Hyg Environ Health. 2011;214: 442–448. doi:10.1016/j.ijheh.2011.08.002

15. ECDC. Consumption of Antibacterials for systemic use (ATC group J01) in the

community (primary care sector) in Europe, reporting year 2018. 2018 pp. 19–20. Available: https://www.ecdc.europa.eu/en/antimicrobial-consumption/database/rates-country

16. Berglund B. Environmental dissemination of antibiotic resistance genes and correlation to anthropogenic contamination with antibiotics. Infect Ecol Epidemiol. 2015;5: 28564. doi:10.3402/iee.v5.28564

17. Marx C, Günther N, Schubert S, Oertel R, Ahnert M, Krebs P, et al. Mass flow of antibiotics in a wastewater treatment plant focusing on removal variations due to operational parameters. Sci Total Environ. 2015;538: 779–788. doi:10.1016/j.scitotenv.2015.08.112

18. Park J, Kim C, Hong Y, Lee W, Chung H, Jeong DH, et al. Distribution and removal of pharmaceuticals in liquid and solid phases in the unit processes of sewage treatment plants. Int J Environ Res Public Health. 2020;17. doi:10.3390/ijerph17030687

19. Östman M, Lindberg RH, Fick J, Björn E, Tysklind M. Screening of biocides, metals and antibiotics in Swedish sewage sludge and wastewater. Water Res. 2017;115: 318–328. doi:10.1016/j.watres.2017.03.011

20. Wang M, Shen W, Yan L, Wang XH, Xu H. Stepwise impact of urban wastewater treatment on the bacterial community structure, antibiotic contents, and prevalence of antimicrobial resistance. Environ Pollut. 2017;231: 1578–1585. doi:10.1016/j.envpol.2017.09.055

21. Giebułtowicz J, Nałęcz-Jawecki G, Harnisz M, Kucharski D, Korzeniewska E, Płaza G. Environmental risk and risk of resistance selection due to antimicrobials' occurrence in two Polish wastewater treatment plants and receiving surface water. Molecules. 2020;25. doi:10.3390/molecules25061470

22. Tran NH, Reinhard M, Gin KYH. Occurrence and fate of emerging contaminants in municipal wastewater treatment plants from different geographical regions-a review. Water Res. 2018;133: 182–207. doi:10.1016/j.watres.2017.12.029

23. Núñez-Delgado A, Pousada-Ferradás Y, Álvarez-Rodríguez E, Fernández-Sanjurjo MJ, Conde-Cid M, Nóvoa-Muñoz JC, et al. Effects of microbiological and non-microbiological treatments of sewage sludge on antibiotics as emerging pollutants present in wastewater: A review. Microb Wastewater Treat. 2019; 1–17. doi:10.1016/B978-0-12-816809-7.00001-4

24. Gogoi A, Mazumder P, Tyagi VK, Tushara Chaminda GG, An AK, Kumar M. Occurrence and fate of emerging contaminants in water environment: A review. Groundw Sustain Dev. 2018;6: 169–180. doi:10.1016/j.gsd.2017.12.009

25. Yang Y, Ok YS, Kim KH, Kwon EE, Tsang YF. Occurrences and removal of pharmaceuticals and personal care products (PPCPs) in drinking water and water/sewage treatment plants: A review. Sci Total Environ. 2017;596–597: 303–320. doi:10.1016/j.scitotenv.2017.04.102

26. Berendonk TU, Manaia CM, Merlin C, Fatta-Kassinos D, Cytryn E, Walsh F, et al. Tackling antibiotic resistance: The environmental framework. Nat Rev Microbiol. 2015;13: 310–317. doi:10.1038/nrmicro3439

27. Di Cesare A, Eckert EM, D'Urso S, Bertoni R, Gillan DC, Wattiez R, et al. Co-occurrence of integrase 1, antibiotic and heavy metal resistance genes in municipal wastewater treatment plants. Water Res. 2016;94: 208–214. doi:10.1016/j.watres.2016.02.049

28. Knopp G, Prasse C, Ternes TA, Cornel P. Elimination of micropollutants and transformation products from a wastewater treatment plant effluent through pilot scale

ozonation followed by various activated carbon and biological filters. Water Res. 2016;100: 580–592. doi:10.1016/j.watres.2016.04.069

29. Wang Q, Liu L, Hou Z, Wang L, Ma D, Yang G, et al. Heavy metal copper accelerates the conjugative transfer of antibiotic resistance genes in freshwater microcosms. Sci Total Environ. 2020;717: 137055. doi:10.1016/j.scitotenv.2020.137055

30. Zarei-Baygi A, Harb M, Wang P, Stadler LB, Smith AL. Evaluating Antibiotic Resistance Gene Correlations with Antibiotic Exposure Conditions in Anaerobic Membrane Bioreactors. Environ Sci Technol. 2019;53: 3599–3609. doi:10.1021/acs.est.9b00798

31. Li LG, Xia Y, Zhang T. Co-occurrence of antibiotic and metal resistance genes revealed in complete genome collection. ISME J. 2017;11: 651–662. doi:10.1038/ismej.2016.155

32. Seiler C, Berendonk TU. Heavy metal driven co-selection of antibiotic resistance in soil and water bodies impacted by agriculture and aquaculture. Front Microbiol. 2012;3: 1–10. doi:10.3389/fmicb.2012.00399

33. Niu H, Cheng W, Pian W, Hu W. The physiochemical properties of submicron particles from emissions of industrial furnace. World J Eng. 2016;13: 218–224. doi:10.1108/WJE-06-2016-029

34. Pal C, Bengtsson-Palme J, Kristiansson E, Larsson DGJ. Co-occurrence of resistance genes to antibiotics, biocides and metals reveals novel insights into their co-selection potential. BMC Genomics. 2015;16: 1–14. doi:10.1186/s12864-015-2153-5

35. Xu Y, Xu J, Mao D, Luo Y. Effect of the selective pressure of sub-lethal level of heavy metals on the fate and distribution of ARGs in the catchment scale. Environ Pollut.

2017;220: 900–908. doi:10.1016/j.envpol.2016.10.074

36. Li H, Duan M, Gu J, Zhang Y, Qian X, Ma J, et al. Effects of bamboo charcoal on antibiotic resistance genes during chicken manure composting. Ecotoxicol Environ Saf. 2017;140: 1–6. doi:10.1016/j.ecoenv.2017.01.007

37. Barancheshme F, Munir M. Strategies to combat antibiotic resistance in the wastewater treatment plants. Front Microbiol. 2018;8. doi:10.3389/fmicb.2017.02603

38. Qian X, Sun W, Gu J, Wang XJ, Sun JJ, Yin YN, et al. Variable effects of oxytetracycline on antibiotic resistance gene abundance and the bacterial community during aerobic composting of cow manure. J Hazard Mater. 2016;315: 61–69. doi:10.1016/j.jhazmat.2016.05.002

39. Deng Y, Bao X, Ji L, Chen L, Liu J, Miao J, et al. Resistance integrons: Class 1, 2 and 3 integrons. Ann Clin Microbiol Antimicrob. 2015;14: 1–11. doi:10.1186/s12941-015-0100-6

40. Pelc W, Pawlas N, Dobrakowski M, Kasperczyk S. Environmental and socioeconomic factors contributing to elevated blood lead levels in children from an industrial area of Upper Silesia. Environ Toxicol Chem. 2016;35: 2597–2603. doi:10.1002/etc.3429

41. Mirończuk-Chodakowska I, Socha K, Zujko ME, Terlikowska KM, Borawska MH, Witkowska AM. Copper, manganese, selenium and zinc in wild-growing edible mushrooms from the eastern territory of “green lungs of Poland”: Nutritional and toxicological implications. Int J Environ Res Public Health. 2019;16: 1–22. doi:10.3390/ijerph16193614

42. Główny Urząd Statystyczny. Ludność. Stan i struktura oraz ruch naturalny w przekroju terytorialnym w 2019r. 2020;1. doi:10.1017/CBO9781107415324.004

43. Zielińska M, Rusanowska P, Jarząbek J, Nielsen JL. Community dynamics of denitrifying bacteria in full-scale wastewater treatment plants. Environ Technol (United Kingdom). 2016;37: 2358–2367. doi:10.1080/09593330.2016.1150350

44. Korzeniewska E, Harnisz M. Extended-spectrum beta-lactamase (ESBL)-positive Enterobacteriaceae in municipal sewage and their emission to the environment. J Environ Manage. 2013;128: 904–911. doi:10.1016/j.jenvman.2013.06.051

45. Kowalska E, Paturej E, Zielińska M. Use of Lecane rotifers for limiting Thiothrix filamentous bacteria in bulking activated sludge in a dairy wastewater treatment plant. Arch Biol Sci. 2014;66: 1371–1378. doi:10.2298/ABS1404371K

46. RCGW. Regional Center for Water and Sewage Management. 2020.

47. Journal of Laws. Rozporządzenie Ministra środowiska w sprawie sposobu prowadzenia oceny zanieczyszczenia powierzchni ziemi. Dz Ustaw Rzeczyposp Pol. 2016; 1–86. Available: http://prawo.sejm.gov.pl/isap.nsf/download.xsp/WDU20160001395/O/D20161395.pdf

48. Osińska A, Korzeniewska E, Harnisz M, Felis E, Bajkacz S, Jachimowicz P, et al. Small-scale wastewater treatment plants as a source of the dissemination of antibiotic resistance genes in the aquatic environment. J Hazard Mater. 2020;381: 121221. doi:10.1016/j.jhazmat.2019.121221

49. Osińska A, Harnisz M, Korzeniewska E. Prevalence of plasmid-mediated multidrug resistance determinants in fluoroquinolone-resistant bacteria isolated from sewage and surface water. Environ Sci Pollut Res. 2016;23: 10818–10831. doi:10.1007/s11356-016-6221-4

50. Bastian M, Heymann S, Jacomy M. Gephi: An Open Source Software for Exploring

and Manipulating Networks. Third Int AAAI Conf Weblogs Soc Media. 2009; 361–362. doi:10.1136/qshc.2004.010033

51. Chong J, Soufan O, Li C, Caraus I, Li S, Bourque G, et al. MetaboAnalyst 4.0: Towards more transparent and integrative metabolomics analysis. Nucleic Acids Res. 2018;46: W486–W494. doi:10.1093/nar/gky310

52. Wang S, Cui Y, Li A, Zhang W, Wang D, Ma J. Fate of antibiotics in three distinct sludge treatment wetlands under different operating conditions. Sci Total Environ. 2019;671: 443–451. doi:10.1016/j.scitotenv.2019.03.147

53. Dong H, Yuan X, Wang W, Qiang Z. Occurrence and removal of antibiotics in ecological and conventional wastewater treatment processes: A field study. J Environ Manage. 2016;178: 11–19. doi:10.1016/j.jenvman.2016.04.037

54. Polesel F, Andersen HR, Trapp S, Plósz BG. Removal of Antibiotics in Biological Wastewater Treatment Systems - A Critical Assessment Using the Activated Sludge Modeling Framework for Xenobiotics (ASM-X). Environ Sci Technol. 2016;50: 10316–10334. doi:10.1021/acs.est.6b01899

55. Kaeseberg T, Zhang J, Schubert S, Oertel R, Siedel H, Krebs P. Sewer sediment-bound antibiotics as a potential environmental risk: Adsorption and desorption affinity of 14 antibiotics and one metabolite. Environ Pollut. 2018;239: 638–647. doi:10.1016/j.envpol.2018.04.075

56. Muñoz M, Autenrieth R, Mendoza-Sanchez I. Environmental occurrence of Oxytetracycline and the potential selection of antibiotic resistance in bacteria. XVI World Water Congr. 2017;1953: 1–15.

57. Ashfaq M, Li Y, Rehman MSU, Zubair M, Mustafa G, Nazar MF, et al. Occurrence,

spatial variation and risk assessment of pharmaceuticals and personal care products in urban wastewater, canal surface water, and their sediments: A case study of Lahore, Pakistan. Sci Total Environ. 2019;688: 653–663. doi:10.1016/j.scitotenv.2019.06.285

58. Bielen A, Šimatović A, Kosić-Vukšić J, Senta I, Ahel M, Babić S, et al. Negative environmental impacts of antibiotic-contaminated effluents from pharmaceutical industries. Water Res. 2017;126: 79–87. doi:10.1016/j.watres.2017.09.019

59. Chunhui Z, Liangliang W, Xiangyu G, Xudan H. Antibiotics in WWTP discharge into the Chaobai River, Beijing. Arch Environ Prot. 2016;42: 48–57. doi:10.1515/aep-2016-0036

60. Rodriguez-Mozaz S, Chamorro S, Marti E, Huerta B, Gros M, Sànchez-Melsió A, et al. Occurrence of antibiotics and antibiotic resistance genes in hospital and urban wastewaters and their impact on the receiving river. Water Res. 2015;69: 234–242. doi:10.1016/j.watres.2014.11.021

61. Tella M, Doelsch E, Letourmy P, Chataing S, Cuoq F, Bravin MN, et al. Investigation of potentially toxic heavy metals in different organic wastes used to fertilize market garden crops. Waste Manag. 2013;33: 184–192. doi:10.1016/j.wasman.2012.07.021

62. Olujimi OO, Fatoki OS, Odendaal J, Daso AP, Oputu O. Preliminary investigation into occurrence and removal of arsenic, cadmium, mercury, and zinc in wastewater treatment plants in cape town and stellenbosch. Polish J Environ Stud. 2012;21: 1755–1765.

63. Edokpayi JN, Odiyo JO, Msagati TAM, Popoola EO. Removal Efficiency of Faecal Indicator Organisms, Nutrients and Heavy Metals from a Peri-Urban Wastewater Treatment Plant in Thohoyandou, Limpopo Province, South Africa. Int J Environ Res Public Health. 2015;12: 7300–7320. doi:10.3390/ijerph120707300

64. Gao P, He S, Huang S, Li K, Liu Z, Xue G, et al. Impacts of coexisting antibiotics, antibacterial residues, and heavy metals on the occurrence of erythromycin resistance genes in urban wastewater. Appl Microbiol Biotechnol. 2015;99: 3971–3980. doi:10.1007/s00253-015-6404-9

65. Jones L, Sullivan T, Kinsella B, Furey A, Regan F. Occurrence of Selected Metals in Wastewater Effluent and Surface Water in Ireland. Anal Lett. 2017;50: 724–737. doi:10.1080/00032719.2016.1194854

66. Chen B, Hao L, Guo X, Wang N, Ye B. Prevalence of antibiotic resistance genes of wastewater and surface water in livestock farms of Jiangsu Province, China. Environ Sci Pollut Res. 2015;22: 13950–13959. doi:10.1007/s11356-015-4636-y

67. Le TH, Ng C, Chen H, Yi XZ, Koh TH, Barkham TMS, et al. Occurrences and characterization of antibiotic-resistant bacteria and genetic determinants of hospital wastewater in a tropical country. Antimicrob Agents Chemother. 2016;60: 7449–7456. doi:10.1128/AAC.01556-16

68. Xiong W, Sun Y, Zhang T, Ding X, Li Y, Wang M, et al. Antibiotics, Antibiotic Resistance Genes, and Bacterial Community Composition in Fresh Water Aquaculture Environment in China. Microb Ecol. 2015;70: 425–432. doi:10.1007/s00248-015-0583-x

69. ECDC. Consumption of Antibacterials for systemic use (ATC group J01) in the community and hospital sector in Europe, reporting year 2018. 2018 pp. 2–3. Available: https://www.ecdc.europa.eu/en/antimicrobial-consumption/database/rates-country

70. Calhau V, Mendes C, Pena A, Mendonça N, Da Silva GJ. Virulence and plasmidic resistance determinants of Escherichia coli isolated from municipal and hospital

wastewater treatment plants. J Water Health. 2015;13: 311–318. doi:10.2166/wh.2014.327

71. Uyaguari MI, Scott GI, Norman RS. Abundance of class 1-3 integrons in South Carolina estuarine ecosystems under high and low levels of anthropogenic influence. Mar Pollut Bull. 2013;76: 77–84. doi:10.1016/j.marpolbul.2013.09.027

72. Buta M, Hubeny J, Zieliński W, Korzeniewska E, Harnisz M, Nowrotek M, et al. The occurrence of integrase genes in different stages of wastewater treatment. J Ecol Eng. 2019;20: 39–45. doi:10.12911/22998993/112797

73. Lu Z, Na G, Gao H, Wang L, Bao C, Yao Z. Fate of sulfonamide resistance genes in estuary environment and effect of anthropogenic activities. Sci Total Environ. 2015;527–528: 429–438. doi:10.1016/j.scitotenv.2015.04.101

74. Makowska N, Koczura R, Mokracka J. Class 1 integrase, sulfonamide and tetracycline resistance genes in wastewater treatment plant and surface water. Chemosphere. 2016;144: 1665–1673. doi:10.1016/j.chemosphere.2015.10.044

75. Du J, Geng J, Ren H, Ding L, Xu K, Zhang Y. Variation of antibiotic resistance genes in municipal wastewater treatment plant with A2O-MBR system. Environ Sci Pollut Res. 2015;22: 3715–3726. doi:10.1007/s11356-014-3552-x

76. Chen H, Zhang M. Occurrence and removal of antibiotic resistance genes in municipal wastewater and rural domestic sewage treatment systems in eastern China. Environ Int. 2013;55: 9–14. doi:10.1016/j.envint.2013.01.019

77. Zhang XX, Zhang T. Occurrence, abundance, and diversity of tetracycline resistance genes in 15 sewage treatment plants across china and other global locations. Environ Sci Technol. 2011;45: 2598–2604. doi:10.1021/es103672x

78. Li J, Cheng W, Xu L, Jiao Y, Baig SA, Chen H. Occurrence and removal of antibiotics and the corresponding resistance genes in wastewater treatment plants: effluents' influence to downstream water environment. Environ Sci Pollut Res. 2016;23: 6826–6835. doi:10.1007/s11356-015-5916-2

79. Mao D, Yu S, Rysz M, Luo Y, Yang F, Li F, et al. Prevalence and proliferation of antibiotic resistance genes in two municipal wastewater treatment plants. Water Res. 2015;85: 458–466. doi:10.1016/j.watres.2015.09.010

80. Kümmerer K. Antibiotics in the aquatic environment - A review - Part I. Chemosphere. 2009;75: 417–434. doi:10.1016/j.chemosphere.2008.11.086

81. Birošová L, Mackuľak T, Bodík I, Ryba J, Škubák J, Grabic R. Pilot study of seasonal occurrence and distribution of antibiotics and drug resistant bacteria in wastewater treatment plants in Slovakia. Sci Total Environ. 2014;490: 440–444. doi:10.1016/j.scitotenv.2014.05.030

82. Luo Y, Mao D, Rysz M, Zhou Q, Zhang H, Xu L, et al. Trends in antibiotic resistance genes occurrence in the Haihe River, China. Environ Sci Technol. 2010;44: 7220–7225. doi:10.1021/es100233w

83. Domańska W. Ochrona Środowiska 2019. 2019; 186–188.

84. Chen Q, An X, Li H, Su J, Ma Y, Zhu YG. Long-term field application of sewage sludge increases the abundance of antibiotic resistance genes in soil. Environ Int. 2016;92–93: 1–10. doi:10.1016/j.envint.2016.03.026

85. Osińska A, Korzeniewska E, Harnisz M, Niestępski S. Quantitative occurrence of antibiotic resistance genes among bacterial populations from wastewater treatment plants using activated sludge. Appl Sci. 2019;9. doi:10.3390/app9030387

86. Proia L, Von Schiller D, Sànchez-Melsió A, Sabater S, Borrego CM, Rodríguez-Mozaz S, et al. Occurrence and persistence of antibiotic resistance genes in river biofilms after wastewater inputs in small rivers. Environ Pollut. 2016;210: 121–128. doi:10.1016/j.envpol.2015.11.035

87. Pruden A, Arabi M, Storteboom HN. Correlation between upstream human activities and riverine antibiotic resistance genes. Environ Sci Technol. 2012;46: 11541–11549. doi:10.1021/es302657r

88. Huang X, Liu C, Li K, Su J, Zhu G, Liu L. Performance of vertical up-flow constructed wetlands on swine wastewater containing tetracyclines and tet genes. Water Res. 2015;70: 109–117. doi:10.1016/j.watres.2014.11.048

89. Karkman A, Do TT, Walsh F, Virta MPJ. Antibiotic-Resistance Genes in Waste Water. Trends Microbiol. 2018;26: 220–228. doi:10.1016/j.tim.2017.09.005

90. Hughes D, Andersson DI. Evolutionary Trajectories to Antibiotic Resistance. Annu Rev Microbiol. 2017;71: 579–596. doi:10.1146/annurev-micro-090816-093813

91. Chihomvu P, Stegmann P, Pillay M. Characterization and Structure Prediction of Partial Length Protein Sequences of pcoA, pcoR and chrB Genes from Heavy Metal Resistant Bacteria from the Klip River, South Africa. Int J Mol Sci. 2015;16: 7352–7374. doi:10.3390/ijms16047352

92. Larsson DGJ. Pollution from drug manufacturing: Review and perspectives. Philos Trans R Soc B Biol Sci. 2014;369. doi:10.1098/rstb.2013.0571

93. Cardoso O, Porcher JM, Sanchez W. Factory-discharged pharmaceuticals could be a relevant source of aquatic environment contamination: Review of evidence and need for knowledge. Chemosphere. 2014;115: 20–30.

doi:10.1016/j.chemosphere.2014.02.004

94. Zhang XH, Xu Y Bin, He XL, Huang L, Ling JY, Zheng L, et al. Occurrence of antibiotic resistance genes in landfill leachate treatment plant and its effluent-receiving soil and surface water. Environ Pollut. 2016;218: 1255–1261. doi:10.1016/j.envpol.2016.08.081

95. He LY, Liu YS, Su HC, Zhao JL, Liu SS, Chen J, et al. Dissemination of antibiotic resistance genes in representative broiler feedlots environments: Identification of indicator ARGs and correlations with environmental variables. Environ Sci Technol. 2014;48: 13120–13129. doi:10.1021/es5041267

96. Xu Y, Guo C, Luo Y, Lv J, Zhang Y, Lin H, et al. Occurrence and distribution of antibiotics, antibiotic resistance genes in the urban rivers in Beijing, China. Environ Pollut. 2016;213: 833–840. doi:10.1016/j.envpol.2016.03.054

97. Xu Y Bin, Hou MY, Li YF, Huang L, Ruan JJ, Zheng L, et al. Distribution of tetracycline resistance genes and AmpC β-lactamase genes in representative non-urban sewage plants and correlations with treatment processes and heavy metals. Chemosphere. 2017;170: 274–281. doi:10.1016/j.chemosphere.2016.12.027

98. Kang C-H, Shin Y, Yu H, Kim S, So J-S. Antibiotic and heavy-metal resistance of Vibrio parahaemolyticus isolated from oysters in Korea. Mar Pollut Bull. 2018;135: 69–74. doi:10.1016/j.marpolbul.2018.07.007

99. Dickinson AW, Power A, Hansen MG, Brandt KK, Piliposian G, Appleby P, et al. Heavy metal pollution and co-selection for antibiotic resistance: A microbial palaeontology approach. Environ Int. 2019;132: 105117. doi:10.1016/j.envint.2019.105117

100. Paul D. Research on heavy metal pollution of river Ganga: A review. Ann Agrar Sci. 2017;15: 278–286. doi:10.1016/j.aasci.2017.04.001

101. Nguyen CC, Hugie CN, Kile ML, Navab-Daneshmand T. Association between heavy metals and antibiotic-resistant human pathogens in environmental reservoirs: A review. Front Environ Sci Eng. 2019;13: 46. doi:10.1007/s11783-019-1129-0

102. Martins VV, Zanetti MOB, Pitondo-Silva A, Stehling EG. Aquatic environments polluted with antibiotics and heavy metals: A human health hazard. Environ Sci Pollut Res. 2014;21: 5873–5878. doi:10.1007/s11356-014-2509-4

103. Liu X, Lv K, Deng C, Yu Z, Shi J, Johnson AC. Persistence and migration of tetracycline, sulfonamide, fluoroquinolone, and macrolide antibiotics in streams using a simulated hydrodynamic system. Environ Pollut. 2019;252: 1532–1538. doi:10.1016/j.envpol.2019.06.095

Fig. 1

Click here to access/download;Figure;Fig. 1.tiff.tif

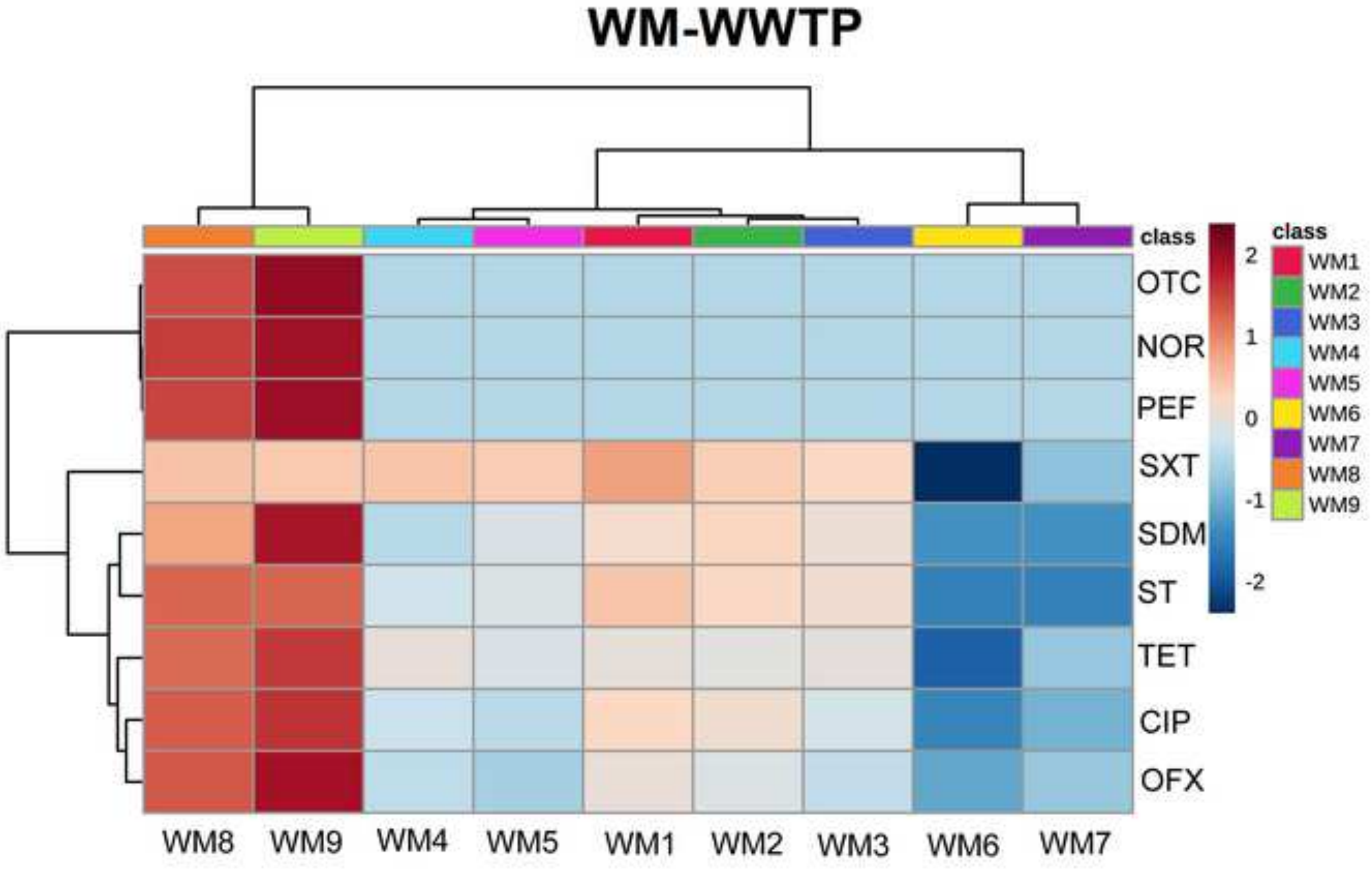

Fig. 2

Click here to access/download;Figure;Fig. 2.tiff.tif

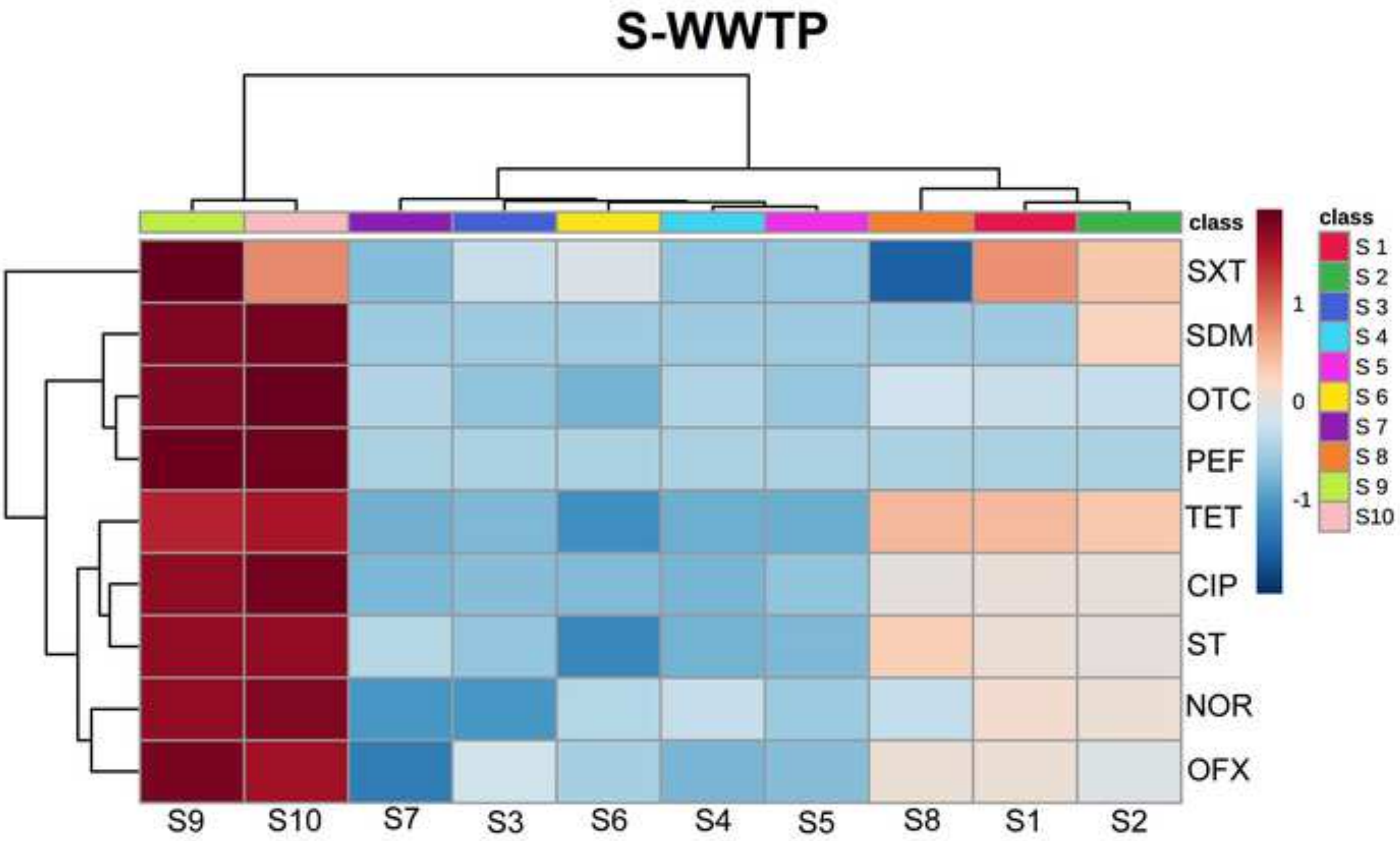

Fig. 3 Click here to access/download;Figure;Fig. 3.tiff.tif

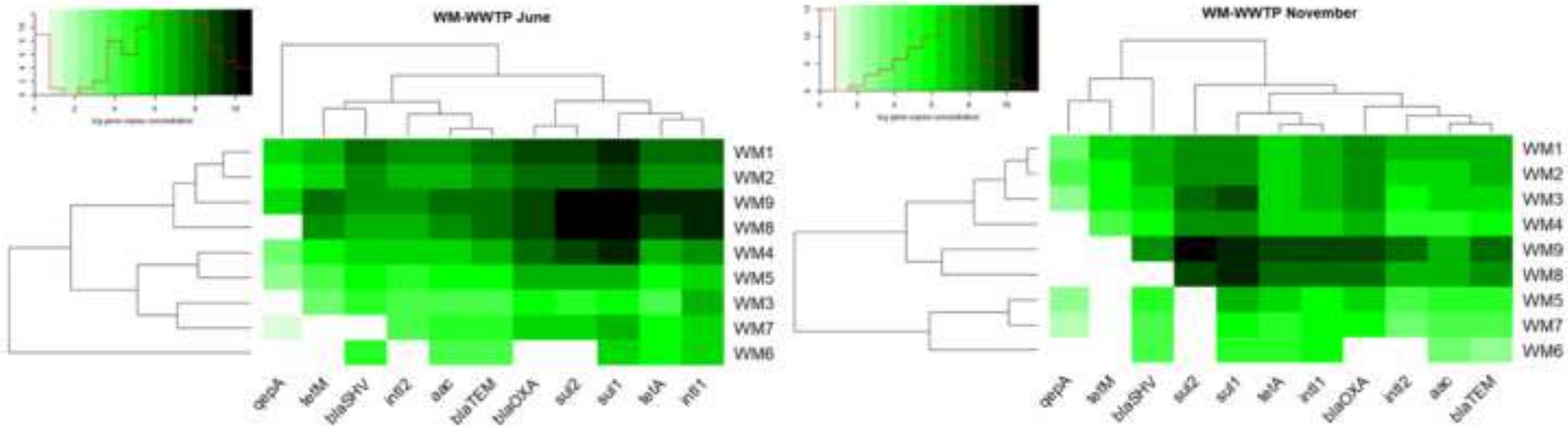

Fig. 4

Click here to access/download;Figure;Fig. 4.tiff.tif

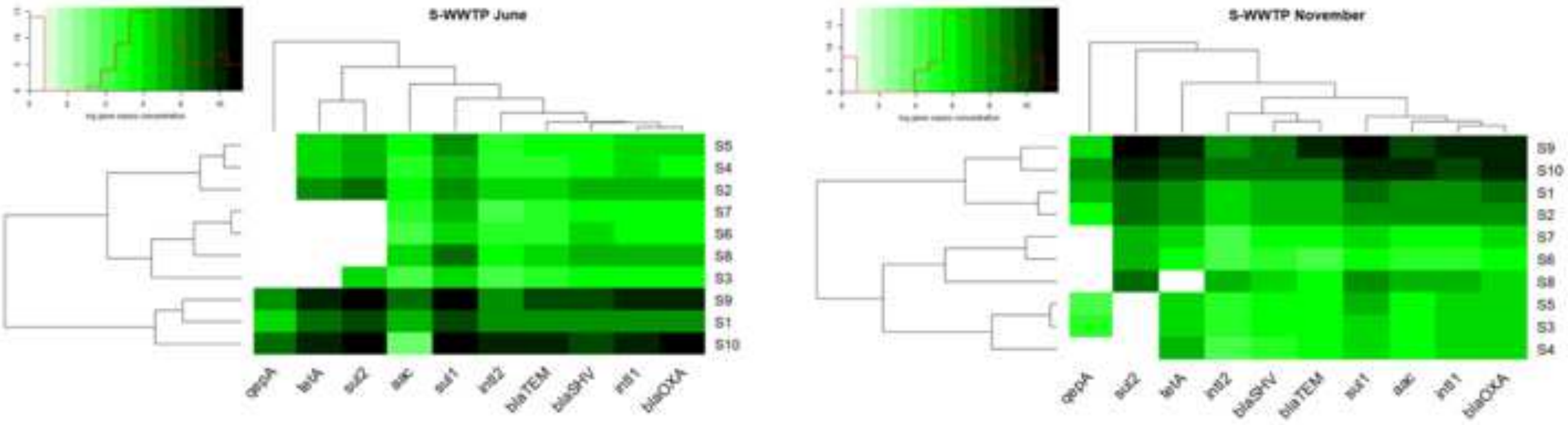

Fig. 5

Click here to access/download;Figure;Fig. 5.tiff.tif

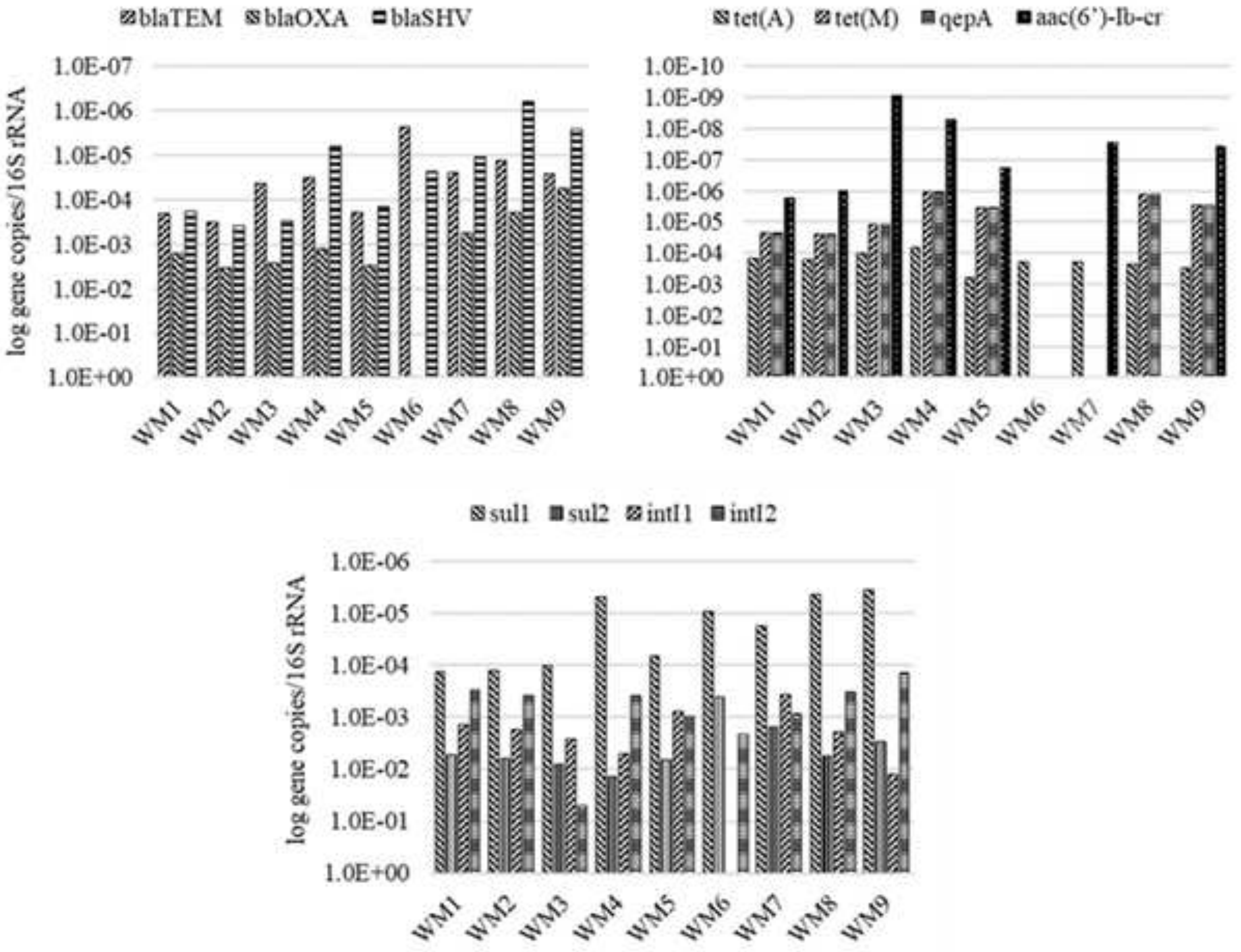

Fig. 6

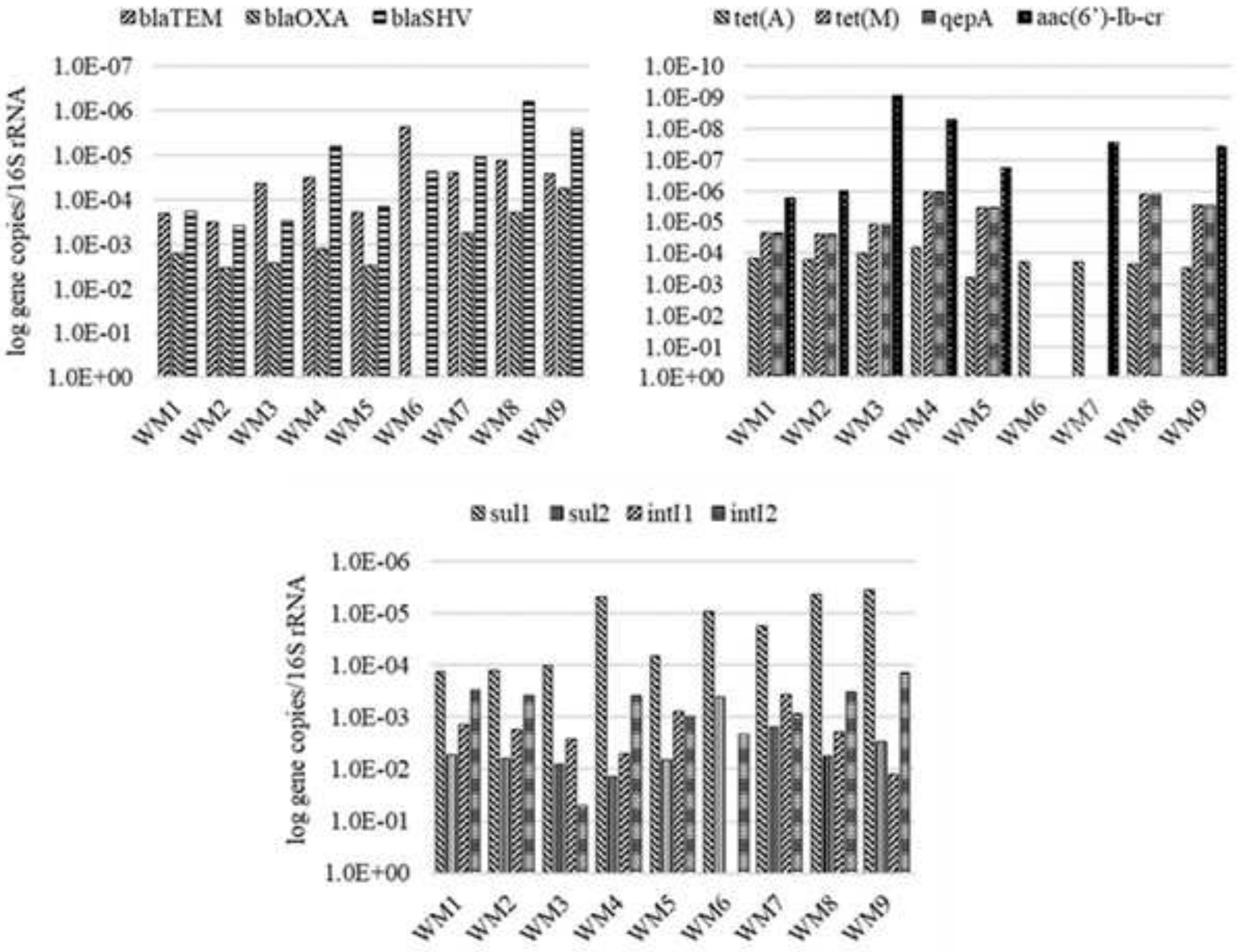
blaTEM
blaOXA
blaSHV
log gene copies/16S rRNA
1.0E-07
1.0E-06
1.0E-05
1.0E-04
1.0E-03
1.0E-02
1.0E-01
1.0E+00
WM1
WM2
WM3
WM4
WM5
WM6
WM7
WM8
WM9
tet(A)
tet(M)
qepA
aac(6')-Ib-cr
1.0E-10
1.0E-09
1.0E-08
1.0E-07
1.0E-06
1.0E-05
1.0E-04
1.0E-03
1.0E-02
1.0E-01
1.0E+00
WM1
WM2
WM3
WM4
WM5
WM6
WM7
WM8
WM9
sul1
sul2
intI1
intI2
log gene copies/16S rRNA
1.0E-06
1.0E-05
1.0E-04
1.0E-03
1.0E-02
1.0E-01
1.0E+00
WM1
WM2
WM3
WM4
WM5
WM6
WM7
WM8
WM9

Fig. 7

Click here to access/download;Figure;Fig. 7.tiff.tif

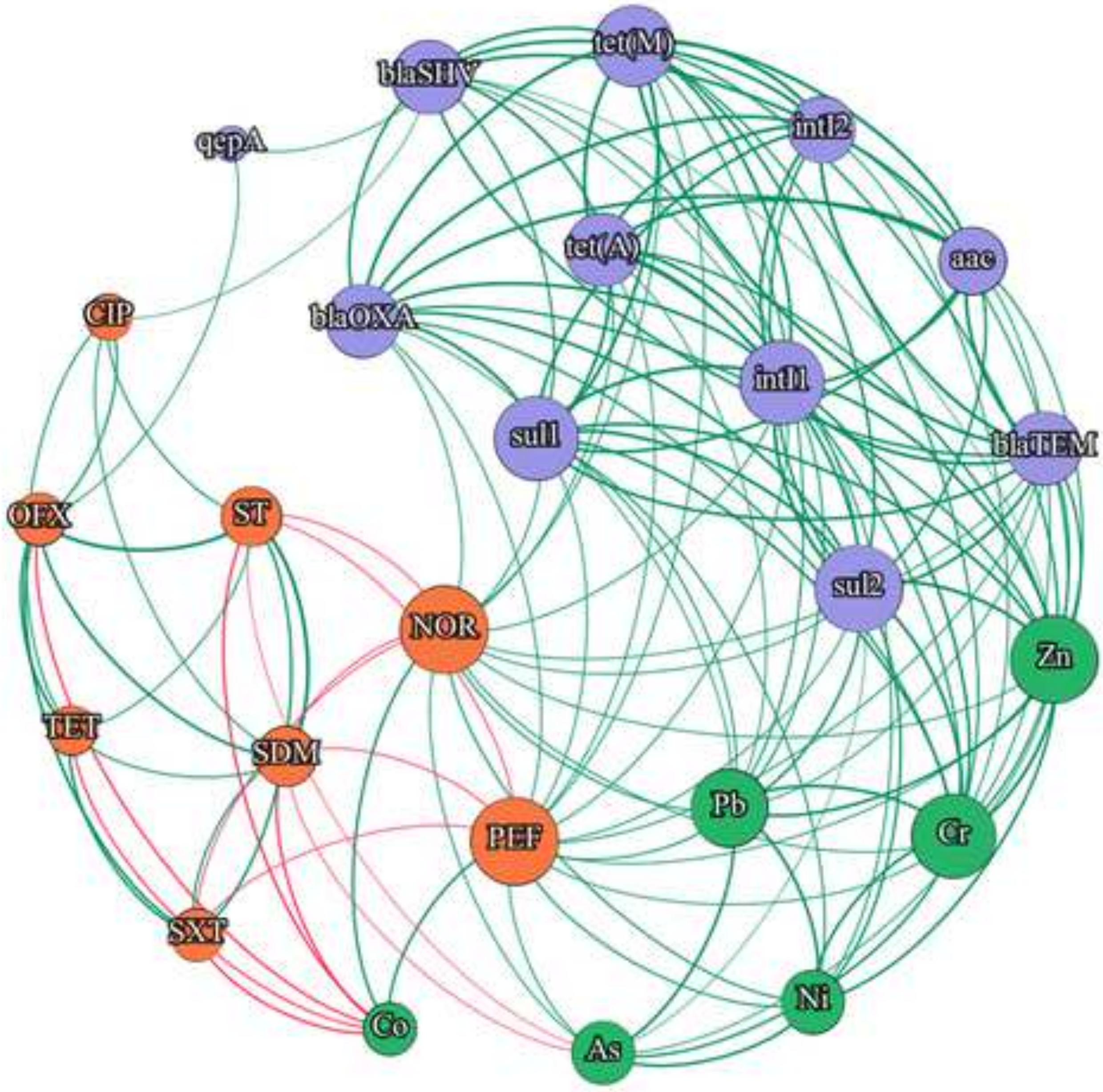

Fig. 8

Click here to access/download;Figure;Fig. 8.tiff.tif

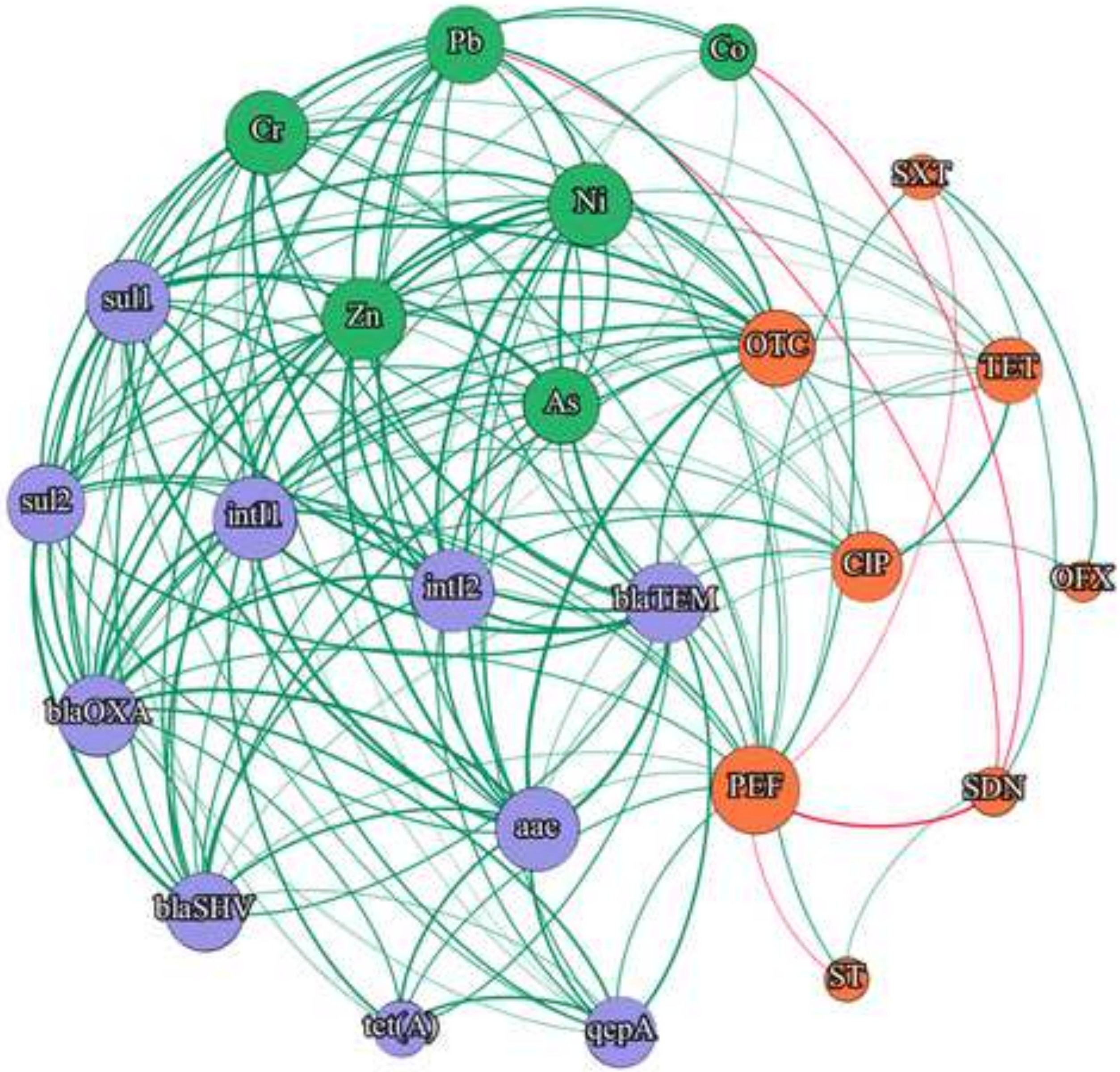

Click here to access/download
Supporting Information
Supporting information.docx
