# Supplementary material for: Industrialization as a source of heavy metals and antibiotics which can enhance the antibiotic resistance in wastewater, sewage sludge and river water
Source: PLoS One. 2021 Jun 4;16(6):e0252691. doi: 10.1371/journal.pone.0252691 (PMC8177550; doi:10.1371/journal.pone.0252691)
Supplement: Attachment — Submitted filename: Response to Reviewers.docx [file pone.0252691.s003.docx]

**We would like to thank the Editor and Reviewer for detailed and helpful suggestions which have been taken into consideration in the revision process. Our responses to specific comments are presented below. The introduced changes and grammatical revisions have been highlighted in yellow in the manuscript.**

**Response to Editor suggestions**

**Comment: “There are a number of areas for improvement identified by the reviewer. In addition, it is very important to differentiate correlation and causation and some of the observed correlation should not be explained as the cause of the observed resistance unless the mechanisms were directly examined. The revised manuscript must be thoroughly revised and all presumptive conclusions (e.g. heavy metals exert a permanent influence or HMs promoting drug resistance) need to be based on experimental data and detailed mechanistic discussions should be included.”**

We are glad of the comment, we have revised the manuscript and have made corrections. We have changed the sentences to avoid drawing straight conclusions based on the presented correlations, in “Impact of industrialization on antibiotic resistance - correlation analysis” subsection and “Conclusions” section, lines 615, 617-619, 643 and 647.

**Response to Reviewer suggestions**

**General revision**

We provided improvements through the manuscript body as reviewer requested:

- we have included suppliers information and details about reagents and consumables,

- we have included model and manufacturer information of all equipment,

- as suggested we have added significant literature,

- as suggested we have included grammatical revision

All introduced to the manuscript changes are highlighted in yellow.

**L2: Not all words in capital letters; Title does not include heavy metals**

We have changed the title to “Industrialization as a source of heavy metals and antibiotics which can enhance the antibiotic resistance in wastewater, sewage sludge and river water”.

**L27: Revise the aim to prevent repetition**

Thank you very much for your suggestion, however, it is difficult for us to understand it and implement significant changes. We do not find in this section the repetitions indicated in the comment.

**L33: Copy numbers?**

Yes. We added “copy numbers” to clarify this sentence.

**L36: Please clarify - was the concentration of the different antibiotics determined as well as the presence of specific resistance genes against these antibiotics which were then correlated?**

To clarify the issue we have added to the sentence “that corresponded to genes encoding resistance” (L35).

**L35: Clarify? or Classes?**

We have changed to “classes” (L36).

**L35: Please clarify and provide an exact value. L37: Please clarify - based on what is this assumption made?**

Regarding to two comments, we have created changes in two sentences, explaining on what is this assumption made (L36-38).

**L86: Provide values**

As suggested we have added the values based on the references (L87-89).

**L97: What about antibiotics? Reduce bacterial load but not resistance genes as such.**

We have added the information about removal of pharmaceuticals from wastewater during treatment process (L99-102).

**L130: Revise not the correct word**

We have revised the mistaken word and changed it (L134).

**L201: Include a complete descriptive heading for the table**

We have changed the table title to be more descriptive and added an expansion for abbreviations (L200-202).

**L354: Please include how this compare with acceptable levels indicated in guidelines**

As suggested we have added the comparison to acceptable levels regulated by Minister of Environment in Poland (L385-391).
