# Supplementary material for: Industrialization as a source of heavy metals and antibiotics which can enhance the antibiotic resistance in wastewater, sewage sludge and river water
Source: PLoS One. 2021 Jun 4;16(6):e0252691. doi: 10.1371/journal.pone.0252691 (PMC8177550; doi:10.1371/journal.pone.0252691)
Supplement: Attachment — Submitted filename: Response to Reviewers.docx [file pone.0252691.s005.docx]

**We would like to thank the Editor and Reviewer for detailed and helpful suggestions which have been taken into consideration in the revision process. Our responses to specific comments are presented below. The introduced changes and grammatical revisions have been highlighted in yellow in the manuscript.**

**Response to Editor suggestions**

**Comment: “Please review your reference list to ensure that it is complete and correct. If you have cited papers that have been retracted, please include the rationale for doing so in the manuscript text, or remove these references and replace them with relevant current references. Any changes to the reference list should be mentioned in the rebuttal letter that accompanies your revised manuscript. If you need to cite a retracted article, indicate the article's retracted status in the References list and also include a citation and full reference for the retraction notice.”**

We are thankful for your suggestion. After a meticulous examination of our paper, we found it complete.

**Response to Reviewer suggestions**

**General revision**

We provided improvements through the manuscript body as reviewer requested:

- we have included addition suppliers information

- as suggested we have included grammatical revision

- as suggested we have checked in references the correctness of bacterial and family names

All introduced to the manuscript changes are highlighted in yellow.
